# Immunoproteasome-specific subunit PSMB9 induction is required to regulate cellular proteostasis upon mitochondrial dysfunction

**DOI:** 10.1038/s41467-023-39642-8

**Published:** 2023-07-11

**Authors:** Minji Kim, Remigiusz A. Serwa, Lukasz Samluk, Ida Suppanz, Agata Kodroń, Tomasz M. Stępkowski, Praveenraj Elancheliyan, Biniyam Tsegaye, Silke Oeljeklaus, Michal Wasilewski, Bettina Warscheid, Agnieszka Chacinska

**Affiliations:** 1 IMol Polish Academy of Sciences, Warsaw, Poland; 2 ReMedy International Research Agenda Unit, IMol Polish Academy of Sciences, Warsaw, Poland; 3 Centre of New Technologies, University of Warsaw, Warsaw, Poland; 4 CIBSS Centre for Integrative Biological Signalling Studies, University of Freiburg, Freiburg, Germany; 5 Department of Biochemistry, Theodor Boveri-Institute, Biocenter, University of Würzburg, Würzburg, Germany; 6 Present Address: Max Planck Institute of Immunobiology and Epigenetics, Freiburg, Germany

**Keywords:** Protein aggregation, Chaperones, Mitochondria, Proteasome

## Abstract

Perturbed cellular protein homeostasis (proteostasis) and mitochondrial dysfunction play an important role in neurodegenerative diseases, however, the interplay between these two phenomena remains unclear. Mitochondrial dysfunction leads to a delay in mitochondrial protein import, causing accumulation of non-imported mitochondrial proteins in the cytosol and challenging proteostasis. Cells respond by increasing proteasome activity and molecular chaperones in yeast and *C. elegans*. Here, we demonstrate that in human cells mitochondrial dysfunction leads to the upregulation of a chaperone HSPB1 and, interestingly, an immunoproteasome-specific subunit PSMB9. Moreover, PSMB9 expression is dependent on the translation elongation factor EEF1A2. These mechanisms constitute a defense response to preserve cellular proteostasis under mitochondrial stress. Our findings define a mode of proteasomal activation through the change in proteasome composition driven by EEF1A2 and its spatial regulation, and are useful to formulate therapies to prevent neurodegenerative diseases.

## Abstract (web summary)

Mitochondrial dysfunction results in the accumulation of mitochondrial proteins in the cytosol. Here, the authors show that the immunoproteasome subunit PSMB9 promotes protein degradation to maintain cellular protein homeostasis.

## Introduction

Mitochondria are multifunctional organelles in eukaryotic cells that participate in energy production, metabolism, apoptosis, and cell signaling, and for that reason, their dysfunction can cause various diseases^1–3^. Although mitochondria have their own DNA, the large majority of mitochondrial proteins are encoded in the nucleus, synthesized as precursor forms in the cytosol and imported into mitochondria by specific transport pathways^4–6^. Mitochondrial dysfunction leads to failure in the import of mitochondrial proteins, thereby accumulating mitochondrial precursor proteins in the cytosol with a potential to challenge cellular protein homeostasis (proteostasis).

To deal with the proteotoxic stress caused by mitochondrial dysfunction, cells have evolved a number of mechanisms that modulate proteostasis network, both inside and outside of mitochondria^7,8^. The mitochondrial unfolded protein response (UPRmt), a stress response mainly studied in *Caenorhabditis elegans* (*C. elegans*), is activated upon the accumulation of unfolded proteins inside mitochondria leading to the induction of the transcription of genes that encode proteases and heat shock proteins (HSPs) located in the mitochondrial matrix through mitochondria-to-nucleus communication^9–11^. In yeast, cytosolic accumulation of mitochondrial precursor proteins initiates the transcription of genes that encode cytosolic HSPs and proteasome subunits^12^. More specifically, the cytosolic appearance of aggregate-prone mitochondrial proteins leads to an overall increase in aggregation of metastable proteins and triggers the overproduction of aggregate-specific HSPs to restore cellular proteostasis^13^.

Cells are also equipped with surveillance systems that monitor mitochondrial protein import at the mitochondrial surface to prevent clogging of the translocase. Upon mitochondrial import failure, the precursor proteins are ubiquitinated and removed by the proteasome^14–16^. The proteasome is one of the main protein degradation machineries that remove damaged, unfolded or misfolded proteins in eukaryotes. It is composed of two multisubunit subcomplexes: the 20 S proteasome which is also called catalytic core particle (CP), and the 19 S regulatory particle (RP)^17–19^.

It is evident that the proteasome has a crucial role in the quality control of mitochondrial proteins^7,8^. In yeast, mitochondrial proteins are degraded by the proteasome not only under mitochondrial protein import failure but also under physiological conditions, indicating that the proteasome continuously monitors and controls mitochondrial proteins in the cytosol^20^. The slowdown of mitochondrial protein import induces a post-transcriptional response, termed unfolded protein response activated by mistargeting of proteins (UPRam), that increases the assembly of proteasome complexes resulting in enhanced proteasome activity^21^. Similarly, mitochondrial defects increase proteasome activity without increasing the production of new proteasome subunits, and extend life span in *C. elegans*^22^. Yet, the mechanisms of proteasomal activation in response to mitochondrial dysfunction remain unclear.

In the present study, we explored stress responses that are activated upon mitochondrial dysfunction in human cells and act to protect the cell against proteotoxicity. We observed selective upregulation of the small HSP family member HSPB1 and the inducible immunoproteasome β subunit PSMB9. We demonstrated that PSMB9 is a key factor responsible for proteasome activity enhancement upon mitochondrial dysfunction. Moreover, we found that, under mitochondrial stress, translation elongation factor EEF1A2 is upregulated which is indispensable for the observed PSMB9 induction. Importantly, depleting PSMB9 or EEF1A2 accelerated protein aggregation, reflecting the roles of those proteins that play to safeguard the cells against mitochondrial dysfunction-mediated proteotoxicity.

## Results

### Mitochondrial complex I deficiency upregulates specific HSPs that are associated with protein aggregation

**Fig. 1:**
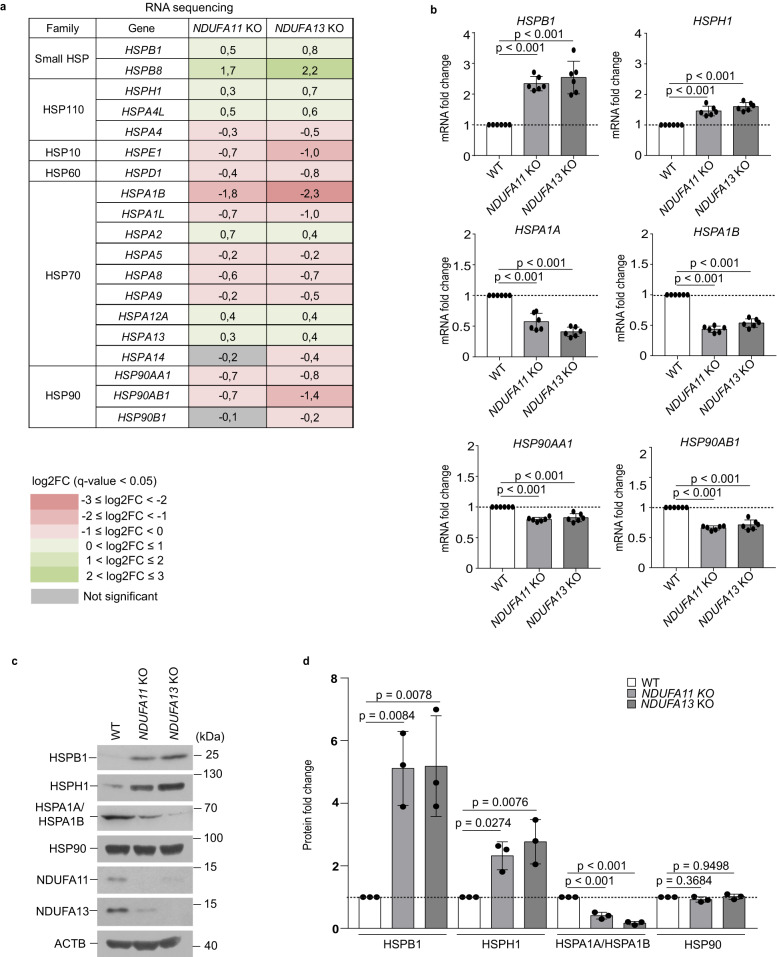
Small HSP and HSP110 families have a tendency to be increased under mitochondrial stress. **a** RNA-seq analysis of HSPs gene expression log2 fold changes (log2FC) in *NDUFA11* KO and *NDUFA13* KO compared to WT HEK293T cells (*n* = 4). Up- and down-regulated genes (*q*-value < 0.05) are shown in green and pink, respectively. The intensity of the color shades depends on the level of expression change. Gray indicates genes with not statistically significant expression changes. **b** mRNA expression patterns of selected transcripts validated by RT-qPCR. The mRNA levels are presented as fold changes relative to WT. Data shown are mean ± SD (*n* = 3 biological replicates with two technical replicates). *p*-value from an ordinary one-way ANOVA with Dunnett’s multiple comparisons test using GraphPad Prism. **c** Western blot analysis of HSPs expression performed in whole cell lysates of *NDUFA11* KO, *NDUFA13* KO and WT HEK293T cells. ACTB was used as a loading control. Data shown are representative of three independent experiments. **d** Quantification of HSPs in western blot analysis normalized to ACTB using ImageJ. The protein levels are presented as fold changes relative to WT. Data shown are mean ± SD (*n* = 3). *p*-value from two-sided, unpaired *t*-test using GraphPad Prism. Source data are provided as a Source Data file.

To study cellular stress responses activated upon mitochondrial dysfunction in humans, we took advantage of two different mitochondrial complex I-deficient human cells, TALEN-based *NDUFA11* knockout (KO) and *NDUFA13* KO human embryonic kidney 293 T (HEK293T) cells^23^. Mitochondrial complex I is the first and the largest enzyme of the mitochondrial respiratory chain. It is composed of 45 subunits in humans, and its deficiency is the most common pathology underlying mitochondrial disorders^24^. NDUFA11 and NDUFA13 are accessory subunits of mitochondrial complex I, and their deficiencies in HEK293T cells cause a significant loss of complex I assembly^23^. We previously found that *NDUFA11* KO has higher reactive oxygen species production and lower ATP generation compared with wild-type (WT) HEK293T cells^25^. In this study, we first investigated the global transcriptomic changes that were triggered by NDUFA11 or NDUFA13 deficiency. In total 13,502 genes were detected, with 651 genes being significantly upregulated (log_2_ fold change > 1, *q*-value < 0.05), and 318 genes being significantly downregulated (log_2_ fold change < −0.75, *q*-value < 0.05) in both *NDUFA11* KO and *NDUFA13* KO compared to WT HEK293T cells (Supplementary Data 1). With these genes, we performed the Reactome pathway enrichment analysis (Supplementary Fig. 1). The analysis showed that the expression of genes involved in translation was significantly downregulated in mitochondrial complex I-deficient HEK293T cells compared with WT HEK293T cells, indicating a cellular attempt to limit the protein burden upon mitochondrial dysfunction. To examine the effect of translation shutdown on mitochondrial and non-mitochondrial proteins in these conditions, we performed mass spectrometry (MS)-based proteomic analysis with total cell extracts of *NDUFA11* KO and WT HEK293T cells. Steady-state levels of most proteins did not reveal profound decreases in *NDUFA11* KO compared to WT HEK293T cells except a few mitochondrial complex I subunits of OXPHOS system in agreement with previous findings^23^ (Supplementary Fig. 2a, b). We then assessed differences in the expression of HSP genes upon mitochondrial dysfunction. The mRNAs encoding small HSP and HSP110 family proteins had a tendency to be increased, while those encoding HSP10, HSP60, HSP70 and HSP90 family proteins had a tendency to be decreased in mitochondrial complex I-deficient HEK293T cells compared with WT in RNA sequencing (RNA-seq) analysis (Fig. 1a). qRT-PCR validation experiments for 6 selected HSPs were consistent with the RNA-seq results (Fig. 1b), so were western blotting (Fig. 1c, d) and MS-based proteomics data validations (Supplementary Fig. 2c). These data showed that HSPB1 and HSPH1 were markedly upregulated at the mRNA and protein levels, whereas levels of HSPA1A/HSPA1B and HSP90 were downregulated or unchanged upon mitochondrial dysfunction. In *NDUFA11* KO, we observed a decrease in NDUFA13 protein expression, and vice versa, as previously reported^23^. These results suggest that selectively upregulated HSPs such as HSPB1 play an important role in protecting cells from protein aggregation appearing under mitochondrial stress conditions.

Small HSP and HSP110 family members were reported to stabilize intermediately folded proteins to prevent their further misfolding and/or aggregation^26–29^. To examine whether the upregulation of HSPB1 and HSPH1 is associated with protein aggregation, we enriched insoluble proteins from total lysates of *NDUFA11* KO and WT HEK293T cells by two subsequent centrifugation steps following previously established protocols^13,30,31^ (Supplementary Fig. 3a), followed by an MS-based proteomic analysis. We found that both HSPB1 and HSPH1 were indeed significantly more abundant in the aggregates isolated from *NDUFA11* KO compared with those from WT HEK293T cells. Among the proteins overrepresented in *NDUFA11* KO aggregates, we also identified 20 S proteasome subunits (red dots), whereas 19 S proteasome subunits (orange dots) were not overrepresented in these aggregates (Supplementary Fig. 3b). In parallel experiments, we confirmed that following the 2-step centrifugation protocol, the bulk of proteasomes remained soluble, as assessed by western blot analysis of its essential component, PSMA1 (Supplementary Fig. 3c). We then compared the protein expression levels of 20 S proteasome subunits in total cell extracts of *NDUFA11* KO and WT HEK293T cells in order to establish whether *NDUFA11* KO cells contain more proteasome subunits than WT cells. However, no apparent difference in levels of these proteins in *NDUFA11* KO compared to WT total cell extracts was observed, it is therefore likely that the overrepresented 20 S proteasomes in aggregates isolated from *NDUFA11* KO cells were specifically associated with proteins aggregating in these cells. We also tested whether *HSPB1* mRNA expression is regulated by heat shock factor protein 1 (HSF1), a well-known master regulator of the heat shock response^32^. There was no change in *HSPB1* mRNA expression, rather it showed a slight tendency to be increased in both mitochondrial complex I-deficient and WT HEK293T cells after *HSF1* knockdown (Supplementary Fig. 3d). Therefore, we infer that the *HSPB1* upregulation is independent of HSF1 upon mitochondrial dysfunction. To conclude, our results indicate that HSPB1, HSPH1 and the proteasome are associated with protein aggregation.

### Mitochondrial complex I deficiency causes a defect in mitochondrial protein import

**Fig. 2:**
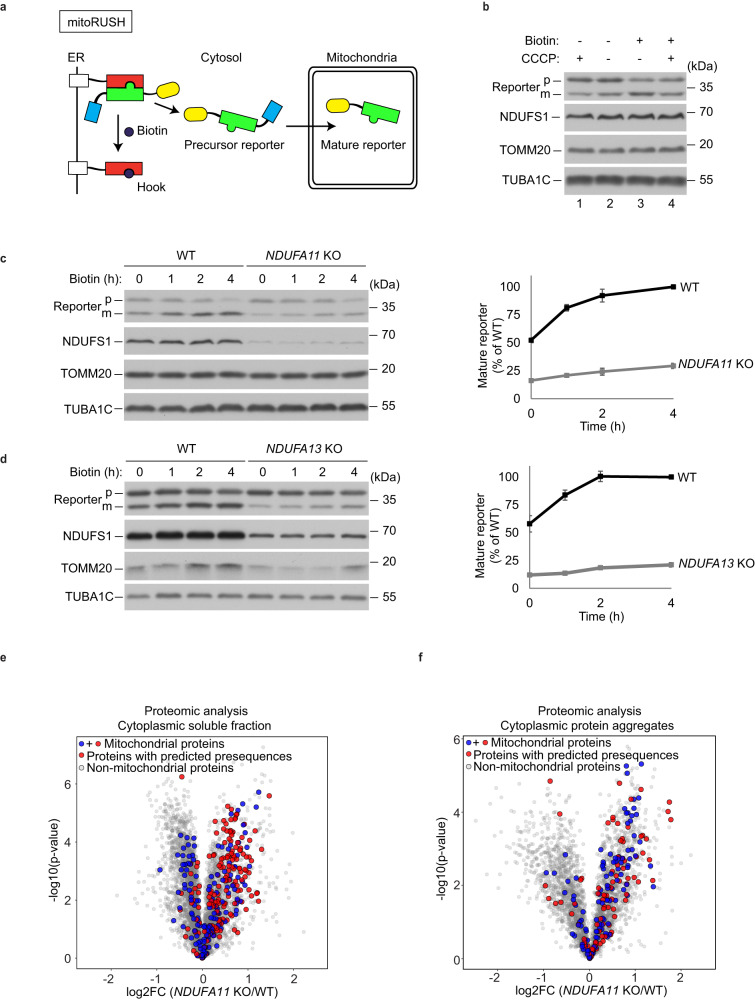
Mitochondrial complex I deficiency leads to an impairment of mitochondrial protein import. **a** Principle of mitoRUSH system used in this study. Red - streptavidin, green - streptavidin-binding peptide, yellow - eYFP, blue - COX8A presequence. **b** Western blot analysis of the reporter protein expression performed in whole cell lysates of WT HEK293T cells treated with or without 40 uM biotin and/or 10 uM CCCP for 4 h. Western blot analysis of the reporter protein expression performed in whole cell lysates of *NDUFA11* KO and WT HEK293T cells (**c**) or *NDUFA13* KO and WT HEK293T cells (**d**) treated with 40 uM biotin up to for 4 h. NDUFS1 was used to show complex I deficiency. TUBA1C was used as a loading control. Data shown are representative of three independent experiments (left panel). Quantified data shown are mean ± SEM (right panel, *n* = 3). p, precursor; m, mature. Volcano plot displaying the log2 fold change (log2FC, x axis) against the *t*-test-derived −log10 statistical *p*-value (y axis) for all protein groups detected in cytoplasmic soluble fractions (**e**) and in cytoplasmic protein aggregates fractions (**f**) of *NDUFA11* KO and WT HEK293T cells by LC-MS/MS analysis (*n* = 3). Student’s *t*-test (two-sided, unpaired) was performed for the statistical analysis. Mitochondrial proteins with or without predicted presequences, and non-mitochondrial proteins are indicated as red, blue, and gray dots, respectively.

As mitochondrial protein import impairment was previously observed when complex I was inhibited^33^, we hypothesized that non-imported mitochondrial proteins into mitochondria from the cytosol may collapse cellular proteostasis and activate stress responses such as HSPB1 induction in mitochondrial complex I-deficient cells. To assess whether complex I deficiency causes mitochondrial protein import defects, we performed an *in organello* mitochondrial protein import assay. As a model protein, we used ornithine transcarbamylase (OTC), which is a presequence-containing protein imported to the mitochondrial matrix by the TIM23 translocase and PAM complex. OTC import requires the presence of mitochondrial inner membrane potential and matrix ATP. An import defect was not observed either in *NDUFA11* KO or in *NDUFA13* KO cells (Supplementary Fig. 4a). This could be attributed to the method of mitochondria energization during import *in organello*. Succinate and ATP presenting in the import buffer support mitochondrial inner membrane potential through complex II and prevent depletion of matrix ATP, respectively. In order to test the efficiency of mitochondrial protein import in an alternative way, we developed *in cellulo* assay called Mitochondrial Retention Using Selective Hooks (mitoRUSH). This method is based on RUSH system that utilizes streptavidin-based interactions to stabilize precursor proteins targeted to mitochondria in the cytosol (Fig. 2a)^34,35^. Cells are transfected with constructs encoding a reporter protein and a hook protein. The reporter protein contains a mitochondria targeting presequence, streptavidin binding domain (SBP) and enhanced yellow fluorescence protein (EYFP). The hook protein contains streptavidin and is anchored in the ER membrane by N-terminal arginine-based motif of human invariant chain of the major histocompatibility complex. Streptavidin of the hook exposed to the cytosol binds the SBP of the reporter with high affinity, thus competing for the precursor proteins with the mitochondrial import machinery. In these conditions, cells accumulated a precursor form of the reporter protein that contains the presequence, and thus could be discriminated from the mature form on the western blot (Fig. 2b, lane 2). The reporter could then be released by biotin, which has a higher affinity towards streptavidin compared with the SBP^36,37^. Within hours after biotin treatment, the mature form of the reporter accumulated (Fig. 2b, lane 3). Import of the reporter could be prevented by dissipating the mitochondrial inner membrane potential with CCCP (Fig. 2b, lane 4). We used the mitoRUSH assay to compare the efficiency of protein import into mitochondria in *NDUFA11* KO, *NDUFA13* KO and WT HEK293T cells. In both mitochondrial complex I-defective cell lines, we observed drastically lower amounts of the mature form of the reporter even if the precursor form was expressed to a similar level as in WT cells (Fig. 2c, d, respectively). Of note, TOMM20 as a mitochondrial marker indicated a lower mitochondrial content in the case of *NDUFA13* KO HEK293T cells, suggesting mitochondrial biogenesis defects. All together, we conclude that although *NDUFA11* KO and *NDUFA13* KO mitochondria contain a fully functional TIM23 and PAM import machinery (Supplementary Fig. 4a), the defect in the respiratory chain leads to a bioenergetic deficit that decreases the efficiency of protein import into mitochondria.

Next, we aimed to examine whether non-imported mitochondrial proteins, which accumulated in the cytosol, are enriched in the aggregates of *NDUFA11* KO HEK293T cells. We modified the protocol for the isolation of aggregates to enable an assessment of the presence of mitochondrial precursors accumulated in the cytosol and in aggregates. This time we first removed mitochondria (and thus eliminated mitochondria-localized and associated proteins from the analysis) and then obtained soluble and insoluble fractions of the cytoplasm following a subsequent centrifugation (Supplementary Fig. 4b). We performed comprehensive proteomics measurements of these fractions and focused our analysis on all detected mitochondrial proteins with predicted presequences^38^ in cytoplasmic soluble fractions and aggregates. Indeed, we found these proteins vastly overrepresented in both the cytoplasmic soluble fraction and in the aggregates of *NDUFA11* KO compared to the corresponding fractions of WT HEK293T cells (Fig. 2e, f). In the cytoplasmic soluble fraction, 307 mitochondrial proteins (blue + red dots) were identified, 164 of them were predicted to contain mitochondrial presequences (red dots). As many as 96 (58%) proteins from the latter category showed significantly increased levels in *NDUFA11* KO compared to WT HEK293T cells (Fig. 2e, Supplementary Data 3). In contrast, we observed that the proteins with predicted presequences identified in the mitochondrial fractions (516 counts, red dots) were not overrepresented in the samples isolated from *NDUFA11* KO compared to WT HEK293T cells, instead they were nearly evenly distributed on both sides of the log2 fold change axis in the volcano plot (Supplementary Fig. 4c). We further compared the relative levels of the proteins with predicted presequences and elevated in *NDUFA11* KO versus WT cytoplasmic soluble fractions to their relative levels in mitochondria isolated from *NDUFA11* KO and WT HEK293T cells. The majority of the proteins most enriched in the cytoplasmic soluble fraction were not elevated in the mitochondrial fractions of *NDUFA11* KO HEK293T cells (Supplementary Fig. 4d). Finally, we performed relative quantification of mitochondrial proteins in the cytoplasmic protein aggregates isolated from *NDUFA11* KO versus WT HEK293T cells. We identified 202 mitochondrial proteins (blue + red dots), 90 of them were predicted to contain mitochondrial presequences (red). 39 proteins (43%) including 18 mitochondrial ribosomal proteins from the latter category showed significantly increased levels while 45 proteins (50%) showed comparable levels in the aggregates isolated from *NDUFA11* KO versus WT HEK293T cells (Fig. 2f, Supplementary Data 3). The enrichment of HSPB1 and HSPH1 in both cytoplasmic and aggregate fractions, and some proteasome subunits in the aggregates originated from *NDUFA11* KO cells supported their association with protein aggregation (Supplementary Fig. 4e, f).

Taken together, these results indicate a clear overrepresentation of presequence-containing mitochondrial proteins in the cytoplasmic soluble fraction and in aggregates but not in the mitochondrial fraction of *NDUFA11* KO cell lysates. Our observations suggest that mitochondrial complex I deficiency leads to a mitochondrial protein import failure, which results in the accumulation of non-imported mitochondrial proteins in the cytosol where they are aggregated.

### Mitochondrial complex I deficiency increases *PSMB9* mRNA levels and proteasome activity

**Fig. 3:**
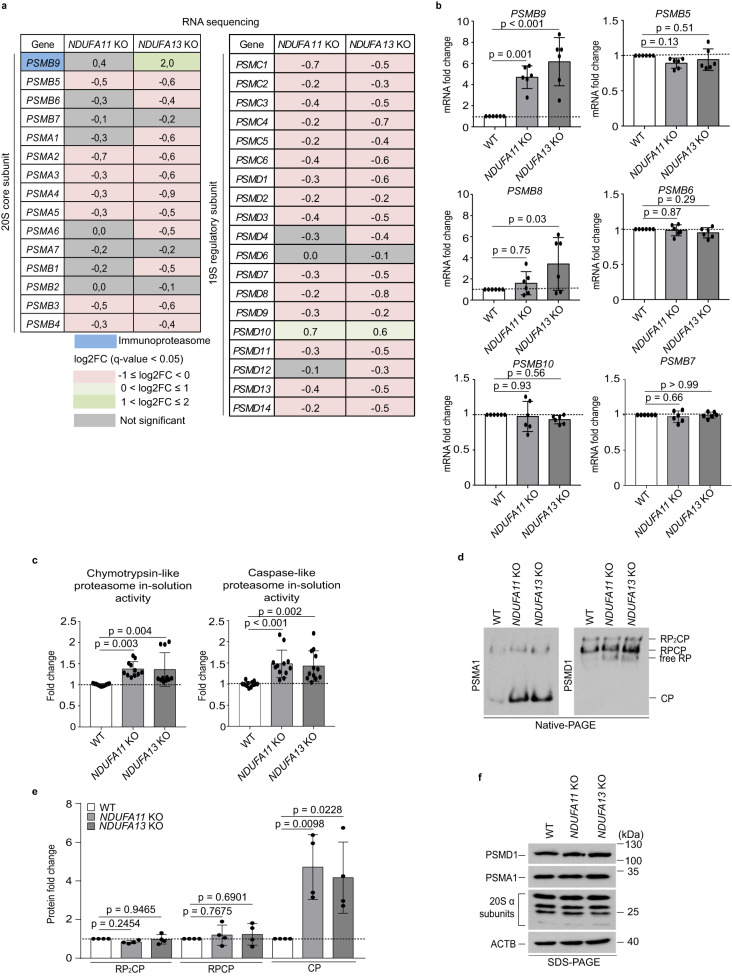
Mitochondrial complex I deficiency increases *PSMB9* mRNA levels and proteasome activity. **a** RNA-seq analysis of 20 S (left panel) and 19 S (right panel) proteasome components gene expression log2 fold changes (log2FC) in mitochondrial complex I-deficient HEK293T cells compared to WT HEK293T cells (*n* = 4). Up- and down-regulated genes (*q*-value < 0.05) are shown in green and pink, respectively. An immunoproteasome subunit is shown in blue. The intensity of the color shades depends on the level of expression change. Gray indicates genes with not statistically significant expression changes. **b** mRNA expression patterns of selected transcripts validated by RT-qPCR. The mRNA levels are presented as fold changes relative to WT. Data shown are mean ± SD (*n* = 3 biological replicates with two technical replicates). *p*-value from an ordinary one-way ANOVA with Dunnett’s multiple comparisons test using GraphPad Prism. **c** Chymotrypsin-like and caspase-like proteasome activities in cell lysates presented as fold changes relative to WT. Data shown are mean ± SD (*n* = 5 biological replicates with one~three technical replicates). **p < 0.01, ***p < 0.001 from an ordinary one-way ANOVA with Dunnett’s multiple comparisons test using GraphPad Prism. **d** Proteasome species in *NDUFA11* KO, *NDUFA13* KO and WT HEK293T cell extracts resolved by electrophoresis in 4.5% native gel followed by western blot analysis detecting a 20 S proteasome subunit PSMA1 and a 19 S proteasome subunit PSMD1 to characterize 26 S (RP_2_CP, doubly capped 26 S; RP_1_CP, singly capped 26 S) and 20 S (CP, core particle) proteasomes. Data shown are representative of four independent experiments. **e** Quantification of proteasomes in **d** using ImageJ. PSMA1 was used to quantify CP, PSMD1 was used to quantify RP_1_CP and RP_2_CP. The protein levels are presented as fold changes relative to WT. Data shown are mean ± SD (*n* = 4). *p*-value from an ordinary one-way ANOVA with Dunnett’s multiple comparisons test using GraphPad Prism. **f** Western blot analysis of proteasome subunit expression performed in whole cell lysates of mitochondrial complex I-deficient and WT HEK293T cells. ACTB was used as a loading control. Data shown are representative of three independent experiments. Source data are provided as a Source Data file.

Given that the 20 S proteasome was more abundant in aggregates of *NDUFA11* KO compared to WT (Supplementary Fig. 3b), we assessed changes in the gene expression of proteasome subunits upon mitochondrial dysfunction. Intriguingly, most genes encoding proteasome subunits were downregulated except from a 20 S proteasome subunit, *PSMB9*, and a 19 S proteasome subunit, *PSMD10* which were upregulated in *NDUFA11* KO and *NDUFA13* KO compared to WT HEK293T cells as shown by RNA-seq analysis (Fig. 3a). The catalytic β subunits PSMB8, PSMB9 and PSMB10 were identified as interferon γ-inducible subunits in higher eukaryotes that are incorporated into a specialized form of the 20 S proteasome, called the immunoproteasome, by replacement of the constitutive 20 S proteasome catalytic β subunits PSMB5, PSMB6 and PSMB7, respectively^39,40^. qRT-PCR validation for *PSMB5*, *PSMB6*, *PSMB7* and *PSMB9* showed expression patterns similar to RNA-seq results. Although *PSMB8* and *PSMB10* were not detected in RNA-seq analysis, they were detected in qRT-PCR analysis. Nevertheless, *PSMB8* was upregulated only in *NDUFA13* KO, and *PSMB10* remained unchanged, while *PSMB9* was upregulated in *NDUFA11* KO and *NDUFA13* KO compared to WT HEK293T cells (Fig. 3b). To investigate the impact of mitochondrial dysfunction on proteasome activity, chymotrypsin-like and caspase-like proteasome activities were measured using fluorogenic peptide substrates. Both activities were significantly higher in *NDUFA11* KO and *NDUFA13* KO compared to WT HEK293T cells, similar to previous observations in yeast and *C. elegans* upon mitochondrial dysfunction^21,22^, demonstrating that mitochondrial defects trigger cellular stress responses such as UPRam also in humans (Fig. 3c). We next examined whether mitochondrial stress alters proteasome assembly using native polyacrylamide gel electrophoresis (PAGE). The 20 S proteasome was more abundant in *NDUFA11* KO and *NDUFA13* KO compared to WT HEK293T cells, whereas the 26 S proteasome remained unchanged (Fig. 3d, e). On the other hand, the protein expression levels of α subunits of the 20 S proteasome and a 19 S proteasome subunit were not changed between mitochondrial complex I-deficient and WT HEK293T cells (Fig. 3f). Since the 20 S proteasome participates in the ubiquitin-independent degradation pathway^19,41,42^, we examined whether global ubiquitination is affected by mitochondrial dysfunction in total cell extracts and aggregate fractions of WT and mitochondrial complex I-deficient HEK293T cells. The expression of ubiquitinated proteins was lower in total cell extracts of *NDUFA11* KO and *NDUFA13* KO compared to those of WT HEK293T cells. Proteasome inhibition by MG132 treatment elevated global protein ubiquitination to similar extent in mitochondrial complex I-deficient and WT HEK293T cells in both total cell extracts and aggregates, suggesting that the proteasome of mitochondrial complex I-deficient cells has higher capacity to degrade polyubiquitinated proteins compared with the proteasome in WT HEK293T cells (Supplementary Fig. 5a, b). In sum, our data indicate that mitochondrial complex I deficiency increases proteasome activity.

### Mitochondrial stress-induced PSMB9 is responsible for proteasome activity enhancement

**Fig. 4:**
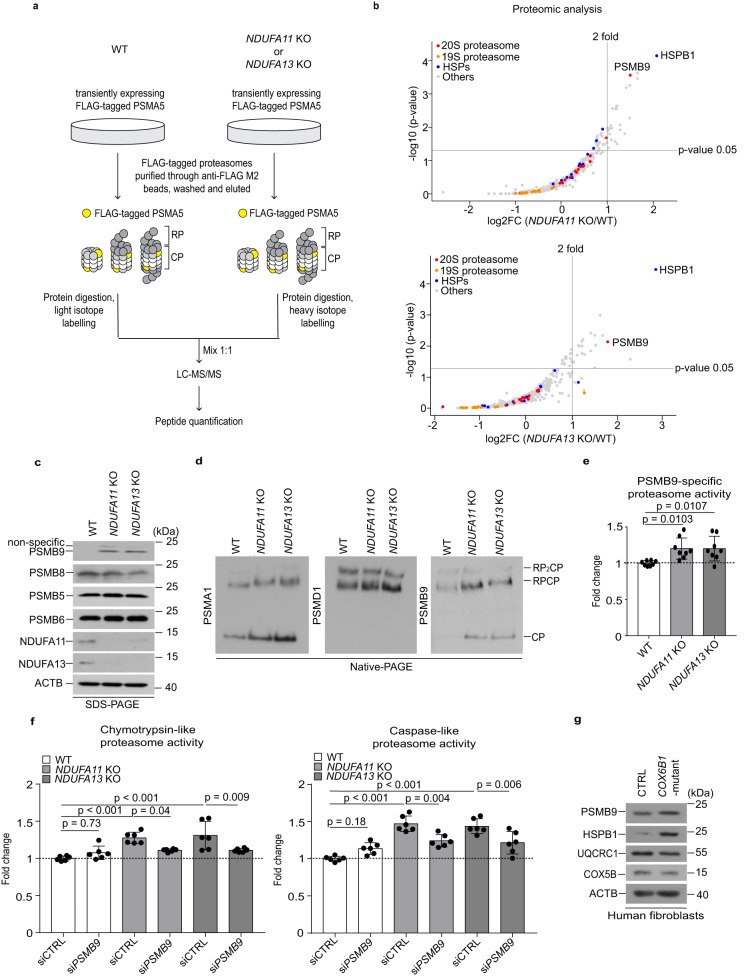
Mitochondrial stress-induced PSMB9 enhances proteasome activity. **a** Workflow of the affinity purification of FLAG-tagged proteasomes by expressing PSMA5-FLAG using anti-FLAG affinity gels for LC-MS/MS analysis. **b** Volcano plots displaying the log2 fold change (log2FC, x axis) against the −log10 statistical *p*-value determined following the rank sum method^90,91^ (y axis) for all proteins quantified in 3/3 replicates of purified proteasomes of *NDUFA11* KO versus WT (upper panel) or *NDUFA13* KO versus WT (lower panel) HEK293T cells by LC-MS/MS analysis (*n* = 3). Using this test, *p*-values were calculated as described by Heskes et al.^92^. 20 S proteasome subunits, 19 S proteasome subunits, HSPs and others are indicated as red, orange, blue, and gray dots, respectively. **c** Western blot analysis of the expression of proteasome β subunits performed in whole cell lysates of mitochondrial complex I-deficient and WT HEK293T cells. ACTB was used as a loading control. Data shown are representative of three independent experiments. **d** Proteasome species in mitochondrial complex I-deficient and WT HEK293T cell extracts resolved by electrophoresis in 4.5% native gel followed by western blot analysis using a 20 S proteasome subunit PSMA1, 20 S immunoproteasome subunit PSMB9 and a 19 S proteasome subunit PSMD1 to characterize 20 S (CP, core particle) and 26 S (RP_2_CP, doubly capped 26 S; RP_1_CP, singly capped 26 S) proteasomes. Data shown are representative of three independent experiments. (**e**) PSMB9-specific proteasome activity in cell lysates presented as fold change relative to WT. Data shown are mean ± SD (n = 4 biological replicates with two technical replicates). *p*-value from an ordinary one-way ANOVA with Dunnett’s multiple comparisons test using GraphPad Prism. **f** Chymotrypsin-like and caspase-like proteasome activities in cell lysates presented as fold changes relative to WT 72 h after transfection with *PSMB9* (si*PSMB9*) or control (siCTRL) siRNA. Data shown are mean ± SD (*n* = 3 biological replicates with two technical replicates). *p*-value from an ordinary one-way ANOVA with Tukey’s multiple comparisons test using GraphPad Prism. **g** Western blot analysis performed in whole cell lysates of *COX6B1*-mutant and control fibroblasts. ACTB was used as a loading control. Data shown are representative of two independent experiments. Source data are provided as a Source Data file.

To characterize the proteasome composition in detail, plasmids expressing FLAG-tagged proteasome subunit alpha type-5 (PSMA5) were generated and expressed in *NDUFA11* KO, *NDUFA13* KO and WT HEK293T cells, and proteasomes were purified by anti-FLAG immunoprecipitation and analyzed using a quantitative MS-based approach (Fig. 4a). As a result, PSMB9 was found to be more abundant in proteasomes purified from both *NDUFA11* KO and *NDUFA13* KO cells compared with those from WT HEK293T cells (Fig. 4b). Of note, this was also observed for HSPB1: it showed increased abundance in proteasome fractions purified from both *NDUFA11* KO and *NDUFA13* KO compared with those from WT HEK293T cells (Fig. 4b). In line with the results of MS-based proteomic analysis, western blot results indicated an induction of PSMB9 protein expression in *NDUFA11* KO and *NDUFA13* KO HEK293T cells. In contrast, there was no difference in the levels of PSMB5, PSMB6 and PSMB8 between mitochondrial complex I-deficient and WT HEK293T cells (Fig. 4c, Supplementary Fig. 6a). We further demonstrated the incorporation of PSMB9 into proteasomes in *NDUFA11* KO and *NDUFA13* KO HEK293T cells by native PAGE (Fig. 4d, Supplementary Fig. 6b), along with an increase in PSMB9-specific proteasome activity in *NDUFA11* KO and *NDUFA13* KO compared to WT HEK293T cells as measured using fluorogenic peptide substrate specific for PSMB9 (Fig. 4e). Moreover, *PSMB9* depletion by small interfering RNA (siRNA) decreased chymotrypsin-like and caspase-like proteasome activities in *NDUFA11* KO and *NDUFA13* KO HEK293T cells (Fig. 4f, Supplementary Fig. 6c), demonstrating the importance of PSMB9 on the proteasome activity enhancement upon mitochondrial dysfunction. We further compared the relative PSMA/PSMB levels in *NDUFA11* KO versus WT HEK293T cells in the proteomics data obtained from aggregates and from isolated proteasomes (Fig. 4b, Supplementary Figs. 3b, 6d). Highly enriched PSMB9 in the isolated proteasomes of *NDUFA11* KO HEK293T cells was not detected in aggregates of *NDUFA11* KO HEK293T cells, suggesting that the PSMB9-containing proteasome may have higher capacity to degrade their substrates than the standard proteasome, so that it maintains its solubility. In a rescue experiment in which we have overexpressed a FLAG-tagged *NDUFA11* in *NDUFA11* KO HEK293T cells, we observed a decrease in PSMB9 and HSPB1, confirming that their induction was elicited by the absence of *NDUFA11* (Supplementary Fig. 6e, f). We then examined whether overexpressed PSMB9 can promote proteasome assembly. Indeed, the overexpressed PSMB9 was incorporated into the proteasome in both WT and mitochondrial complex I-deficient cells. On the other hand, the total amount of the proteasome was unchanged (Supplementary Fig. 6g). It implies that the presence of other subunits and/or assembly factors was a limiting factor for de novo assembly of immunoproteasomes.

To investigate whether our observations from mitochondrial complex I-deficient HEK293T cells are relevant to other mitochondrial disease models, we performed additional experiments using human patient fibroblasts characterized by a different type of a respiratory defect. Those fibroblasts were derived from a patient with a mutation in a nucleus-encoded mitochondrial respiratory chain complex IV subunit COX6B1. This mutation manifests itself with an early-onset encephalopathy with leukodystrophy, myopathy and growth retardation associated with cytochrome c oxidase (COX) deficiency^43^. *COX6B1*-mutant fibroblasts had lower protein expression of UQCRC1, a subunit of mitochondrial complex III, and COX5B, a subunit of mitochondrial complex IV, indicating mitochondrial defects. Importantly, elevated protein expression of PSMB9 and HSPB1 was observed in *COX6B1*-mutant fibroblasts, and these cells showed mitochondrial protein import failure, suggesting that a similar stress response is activated by COX deficiency in fibroblasts (Fig. 4g, Supplementary Fig. 6h).

We reanalyzed published transcriptomic and proteomics data of HeLa cells treated by doxycycline (mitochondrial translation inhibitor), actinonin (mitochondrial protein synthesis inhibitor), carbonylcyanide-4-(trifluoromethoxy)phenylhydrazone (FCCP; mitochondrial depolarizer), and MitoBloCK-6 (MIA40/ALR pathway inhibitor)^44,45^ to see how those compounds affect PSMB9 and/or HSPB1 induction. Interestingly, PSMB9 gene and protein expressions were upregulated when the MIA40-dependent mitochondrial protein import pathway was inhibited in HeLa cells (Supplementary Fig. 6i).

The immunoproteasome has been previously described as an important player in maintaining proteostasis during oxidative stress^46^. We investigated whether HEK293T cells with mitochondrial complex I deficiency and *COX6B1*-mutant fibroblasts generate more reactive oxygen species (ROS) than control cells. ROS levels were higher in mitochondrial complex I-deficient HEK293T cells than in WT HEK293T cells, whereas *COX6B1*-mutant fibroblasts did not generate higher ROS levels than control fibroblasts (Supplementary Fig. 7a). WT HEK293T cells treated for 24 hours (h) with menadione, the mitochondrial ROS inducer, generated similar levels of ROS as HEK293T cells with mitochondrial complex I deficiency, and the treatment with the ROS scavenger N-acetyl-L-cysteine (NAC) effectively reduced the ROS levels increased by menadione (Supplementary Fig. 7b). Therefore, we examined whether the treatment with the NAC can attenuate the induction of PSMB9 and HSPB1. The protein expressions of PSMB9 and HSPB1 were not affected by NAC treatment, suggesting that their induction in *NDUFA11* KO and *NDUFA13* KO is ROS-independent (Supplementary Fig. 7c, d).

Next, we tested whether mitochondrial stress triggered by chemical treatments in WT cells induces PSMB9 and HSPB1 expression similarly as in mitochondrial complex I-deficient HEK293T cells. To this end, WT HEK293T cells were treated with rotenone (mitochondrial complex I inhibitor), menadione, and carbonyl cyanide m-chlorophenylhydrazone (CCCP; mitochondrial depolarizer) for 2 and 24 h followed by western blot analysis. None of the treatments induced PSMB9 and HSPB1 protein expression (Supplementary Fig. 7e, f). To sum up, our data show that PSMB9 in mitochondrial complex I-deficient cells is responsible for enhancement of proteasome activity upon mitochondrial stress and its induction is ROS-independent.

### Mitochondrial stress-induced EEF1A2 regulates PSMB9 expression

**Fig. 5:**
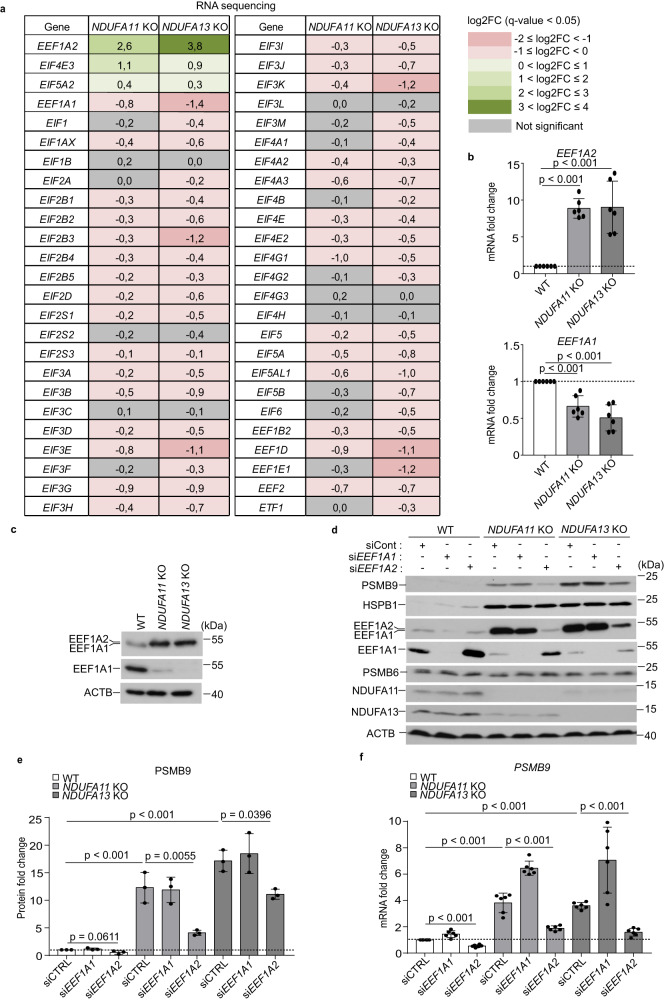
EEF1A2 are induced under mitochondrial stress. **a** RNA-seq analysis of translation factor gene expression log2 fold changes (log2FC) in mitochondrial complex I-deficient cells compared to WT HEK293T cells (*n* = 4). Up- and down-regulated genes (*q*-value < 0.05) are shown in green and pink, respectively. The intensity of the color shades depends on the level of expression change. Gray indicates genes with not statistically significant expression changes. **b** mRNA expression patterns of *EEF1A2* and *EEF1A1* by RT-qPCR. The mRNA levels are presented as fold changes relative to WT. Data shown are mean ± SD (*n* = 3 biological replicates with two technical replicates). *p*-value from an ordinary one-way ANOVA with Dunnett’s multiple comparisons test using GraphPad Prism. **c** Western blot analysis of EEF1A1 and EEF1A2 performed in whole cell lysates of WT and mitochondrial complex I-deficient HEK293T cells. Anti-EEF1A2 antibody was used in the first blot, anti-EEF1A1 antibody was used in the second blot from the top. Mitochondrial complex I-deficient HEK293T cells and WT HEK293T cells were transfected with *EEF1A1* (si*EEF1A1*), *EEF1A2* (si*EEF1A2*) or control (siCTRL) siRNA for 72 h. **d** Western blot analysis performed in whole cell lysates of mitochondrial complex I-deficient HEK293T cells and WT HEK293T cells. ACTB was used as a loading control. Data shown are representative of three independent experiments. Anti-EEF1A2 antibody was used in the third blot, anti-EEF1A1 antibody was used in the fourth blot from the top. **e** Quantification of PSMB9 in **d** normalize**d** to ACTB using ImageJ. The protein levels are presented as fold changes relative to WT. Data shown are mean ± SD (*n* = 3). *p*-value from an ordinary one-way ANOVA with Dunnett’s multiple comparisons test using GraphPad Prism. **f** *PSMB9* mRNA expression pattern examined by RT-qPCR in WT and mitochondrial complex I-deficient HEK293T cells. The mRNA levels are presented as fold changes relative to WT transfected with control siRNA. Data shown are mean ± SD (*n* = 3 biological replicates with two technical replicates). *p*-value from two-sided, unpaired *t*-test using GraphPad Prism. Source data are provided as a Source Data file.

Translation occurs in three steps: initiation, elongation, and termination. Aminoacylated-tRNAs are delivered to the ribosome by the translation elongation factor EEF1A. The role of EEF1A is guanosine triphosphate (GTP)-dependent, and this process is facilitated by a GTP-exchange factor called EEF1B. In mammals, there are two isoforms of EEF1A, EEF1A1 and EEF1A2, with EEF1A1 being a house-keeping factor in most cells^47,48^. Unexpectedly, we observed that EEF1A2 is strongly enriched not only in the cytoplasmic soluble fraction but also in aggregates of *NDUFA11* KO compared to WT HEK293T cells (Supplementary Figs. 3b, and 4e, f). Thus, we assessed changes in gene expression of translation factors including EEF1A2 upon mitochondrial dysfunction. Our RNA-seq analysis revealed that the majority of genes encoding translation factors were downregulated whereas only three genes were upregulated, with *EEF1A2* being the most upregulated gene among those in *NDUFA11* KO and *NDUFA13* KO compared to WT HEK293T cells in RNA-seq analysis (Fig. 5a). The upregulation of *EEF1A2* and downregulation of its isoform *EEF1A1* were confirmed by qRT-PCR, western blot (Fig. 5b, c) as well as MS-based proteomics data (Supplementary Fig. 2c). It was previously shown that EEF1A1 migrates slightly faster than EEF1A2 as deduced by western blotting with a specific antibody against EEF1A2^49^, and we also observed the EEF1A2 band of WT HEK293T cells are slightly lower than those of mitochondrial complex I-deficient cells (Fig. 5c). We then checked the specificity of the bands by single and double knockdowns of *EEF1A1* and *EEF1A2*. The EEF1A2 band of WT HEK293T cells detected by anti-EEF1A2 antibody was still present after *EEF1A2* depletion, and this band disappeared when *EEF1A1* was also depleted (Supplementary Fig. 8a). Thus, the slightly lower band of WT HEK293T cells detected by anti-EEF1A2 antibody was revealed as EEF1A1. On the other hand, the band of EEF1A2 detected by anti-EEF1A2 antibody disappeared after *EEF1A2* depletion but not after *EEF1A1* depletion in mitochondrial complex I-deficient cells, showing that EEF1A2 is the major EEF1A isoform in these conditions (Supplementary Fig. 8a). Anti-EEF1A1 antibody specifically detected EEF1A1 in both mitochondrial complex I-deficient and WT HEK293T cells, allowing us to compare the protein expression of EEF1A1 and EEF1A2 (Supplementary Fig. 8a). To better understand the interplay between EEF1A1 and EEF1A2 in the translation, protein synthesis was measured in EEF1A1 or EEF1A2-depleted WT HEK293T cells. EEF1A2 depletion promoted protein translation while EEF1A1 depletion did not show a significant change, suggesting that EEF1A2 has an inhibitory role of protein synthesis (Supplementary Fig. 8b, c).

To investigate the role of EEF1A2 in PSMB9 and HSPB1 protein expression, we used siRNAs to deplete *EEF1A2* in mitochondrial complex I-deficient and WT HEK293T cells. *EEF1A1* was also depleted to compare the effects of the two different translation elongation factors on PSMB9 and HSPB1 protein expression. Surprisingly, *EEF1A2* knockdown significantly reduced not only the PSMB9 protein but also mRNA expression of *PSMB9* (Fig. 5d–f). *EEF1A1* knockdown increased *PSMB9* mRNA expression, however, PSMB9 protein expression was not much affected (Fig. 5d–f). Neither *EEF1A1* nor *EEF1A2* knockdown changed the mRNA and protein expression of *HSPB1 and PSMB6* (Fig. 5d, Supplementary Fig. 8d). Furthermore, we found evidence for compensatory mechanisms underlying mutual negative feedback regulation between EEF1A1 and EEF1A2. *EEF1A1* knockdown markedly increased *EEF1A2* mRNA expression, whereas its protein expression was unaffected in both mitochondrial complex I-deficient and WT HEK293T cells. *EEF1A2* knockdown did not change mRNA expression of *EEF1A1*, whereas EEF1A1 protein expression was increased in both mitochondrial complex I-deficient and WT HEK293T cells, indicating that cells have complicated regulatory mechanisms underlying the process linking gene expression to protein synthesis of EEF1A1 and EEF1A2 (Fig. 5d, Supplementary Fig. 8d).

Beyond cytoplasmic functions of EEF1A2 as an translation elongation factor, it may also have nuclear functions, because it was localized not only in the cytoplasm but also in the nucleus (Supplementary Fig. 8e) as reported in previous studies^50^. To test the possibility that induced EEF1A2 stabilizes *PSMB9* mRNA, *PSMB9* mRNA stability was examined after a transcription inhibitor actinomycin D (ActD) treatment up to 24 h. *PSMB9* mRNA stability was not higher in mitochondrial complex I-deficient HEK293T cells compared to WT HEK293T cells (Supplementary Fig. 8f), therefore, EEF1A2 regulates the transcription and translation of PSMB9 rather than its mRNA stability although its exact function in the nucleus is yet to be investigated.

### PSMB9 and HSPB1 are required to maintain cellular proteostasis upon mitochondrial dysfunction

**Fig. 6:**
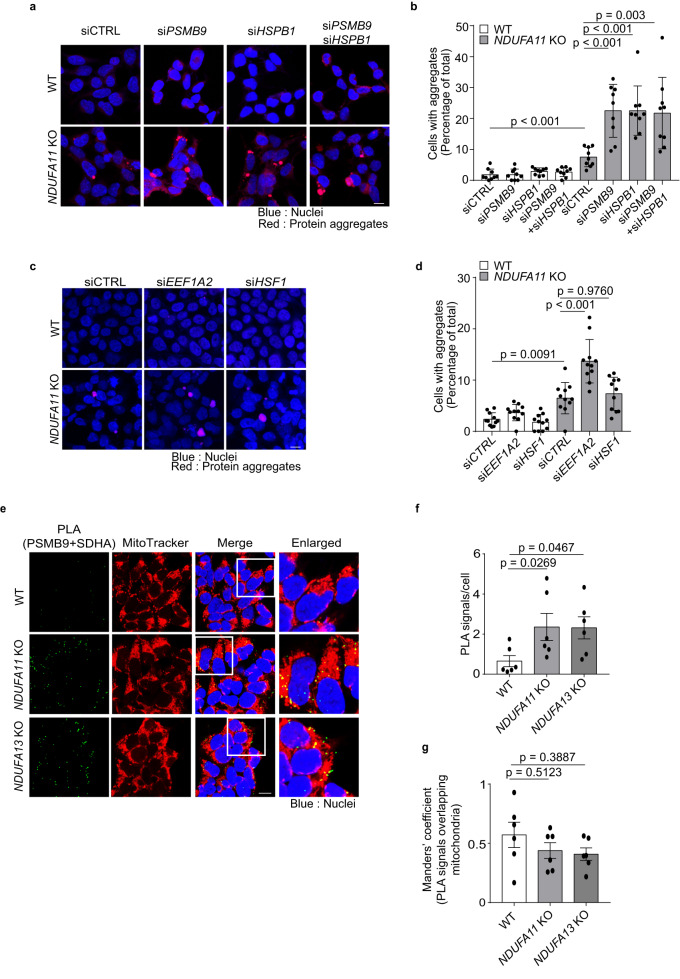
PSMB9 and HSPB1 are required to maintain proteostasis upon mitochondrial dysfunction. **a**, **b** HEK293T cells were transfected with *PSMB9* (si*PSMB9*), *HSPB1* (si*HSPB1*) or control (siCTRL) siRNA for 72 h. **a** Images of protein aggregates stained with PROTEOSTAT® dye. Data shown are representative of three independent experiments. The scale bar represents 10 μm. **b** Quantification of the percentage of cells containing aggregates in **a**. Counted cell numbers of WT are 1152, 754, 662, 1099; *NDUFA11 KO* are 787, 534, 677, 794 from left to right. Data shown are mean ± SD (nine sight fields from three independent experiments). *p*-value from two-sided, unpaired *t*-test or Mann Whitney test using GraphPad Prism. **c**, **d** HEK293T cells were transfected with *EEF1A2* (si*EEF1A2*), *HSF1* (si*HSF1*) or control (siCTRL) siRNA for 72 h. **c** Images of protein aggregates stained with PROTEOSTAT® dye. Data shown are representative of three independent experiments. The scale bar represents 10 μm. **d** Quantification of the percentage of cells containing aggregates in **c**. Counted cell numbers of WT are 3776, 3164, 3659; *NDUFA11 KO* are 3161, 2462, 3256 from left to right. Data shown are mean ± SD (eleven sight fields from three independent experiments). *p*-value from an ordinary one-way ANOVA with Tukey’s multiple comparisons test using GraphPad Prism. **e** Images of PLA performed between SDHA and PSMB9 with co-staining of mitochondria by MitoTracker Deep Red in WT and mitochondrial complex I-deficient cells. Data shown are representative of three independent experiments. The scale bar represents 10 μm. **f** Quantification of PLA signals per cell in **e** of WT (*n* = 102), *NDUFA11 KO* (*n* = 110) and *NDUFA13 KO* (*n* = 89) HEK293T cells. Data shown are mean ± SD (six sight fields from three independent experiments). *p*-value from an ordinary one-way ANOVA with Dunnett’s multiple comparisons test using GraphPad Prism. **g** Quantification of the Manders’ coefficient in **e** of WT (*n* = 102), *NDUFA11 KO* (*n* = 110) and *NDUFA13 KO* (*n* = 89) HEK293T cells. Data shown are mean ± SD (six sight fields from three independent experiments). *p*-value from two-sided, unpaired *t*-test or Kruskal-Wallis test with Dunn’s multiple comparisons test using GraphPad Prism. Source data are provided as a Source Data file.

Mitochondrial dysfunction has been reported to promote protein aggregate formation in yeast, *C. elegans* and human cells^13,51^. Hence, we investigated whether mitochondrial complex I deficiency triggers protein aggregation, and whether PSMB9 and HSPB1 have physiological functions on the accumulation of protein aggregates. To elucidate the effect of PSMB9 and HSPB1 in protein aggregation, *PSMB9* or *HSPB1* was depleted using siRNAs (Supplementary Fig. 9a), and protein aggregates in *NDUFA11* KO and WT HEK293T cells were stained with PROTEOSTAT® dye, and monitored by confocal microscopy. *NDUFA11* KO displayed more protein aggregates than WT HEK293T cells, and *PSMB9* or *HSPB1* single knockdown significantly increased protein aggregation in *NDUFA11* KO HEK293T cells (Fig. 6a, b). *PSMB9* and *HSPB1* double knockdown showed a similar deleterious effect as single depletion of either *PSMB9* or *HSPB1*, implying that those proteins act interdependently to protect cells from protein aggregation under mitochondrial stress. Conversely, no increase in protein aggregates was observed in WT HEK293T cells (Fig. 6a, b). We also examined the effect of *PSMB6* silencing on proteasome activities and protein aggregation. *PSMB6* knockdown significantly decreased chymotrypsin-like and caspase-like proteasome activities in both mitochondrial complex I-deficient and WT HEK293T cells (Supplementary Fig. 9b, c). However, unlike *PSMB9* knockdown, *PSMB6* knockdown did not increase protein aggregation in both *NDUFA11* KO and WT HEK293T cells, emphasizing the important role of PSMB9 playing to prevent protein aggregation upon mitochondrial dysfunction (Supplementary Fig. 9d, e). We extended the studies on observation of protein aggregates to cells after *EEF1A2* or *HSF1* knockdown. *EEF1A2* knockdown remarkably promoted protein aggregate formation in *NDUFA11* KO HEK293T cells whereas *HSF1* knockdown did not in *NDUFA11* KO HEK293T cells. Neither *EEF1A2* nor *HSF1* knockdown affected protein aggregate formation in WT HEK293T cells (Fig. 6c, d). These findings demonstrate that PSMB9 and HSPB1 prevent protein aggregation upon mitochondrial dysfunction, and support the idea that EEF1A2, the mediator of PSMB9 expression, is an important factor to maintain a balanced proteome under mitochondrial stress.

We examined the location of PSMB9-containing proteasomes in a close proximity to mitochondria using proximity ligation assay (PLA), a method to identify physical closeness of two proteins where a signal will only be produced if they are closer than 40 nm. For this assay, PSMB9 and a mitochondrial complex II subunit SDHA were stained to detect PSMB9-containing proteasomes and mitochondria, respectively. The PLA signal was significantly increased in *NDUFA11* KO and *NDUFA13* KO compared with WT HEK293T cells, indicating that higher expression of PSMB9 led to enhance colocalization of PSMB9 with mitochondria (Fig. 6e, f). To validate the mitochondrial location of PLA signals, cells were co-stained with MitoTracker Deep Red, a well-known mitochondrial fluorescent probe, and the PLA signals were moderately colocalized with mitochondria in both mitochondrial complex I-deficient and WT HEK293T cells although the PLA signals were much less in WT compared with those in the mitochondrial complex I-deficient HEK293T cells as expected (Fig. 6e, g). MitoTracker Deep Red is mitochondrial inner membrane potential-dependent^52^, therefore, it may not be able to fully stain mitochondria. So we additionally validated the location of SDHA in mitochondria by staining with another mitochondrial protein TOMM20. Indeed, most SDHA were colocalized with TOMM20 (Supplementary Fig. 9f, g). The constitutive proteasome subunit PSMB6 was also stained with SDHA for PLA analysis, however, no PLA signal was detected in both mitochondrial complex I-deficient and WT HEK293T cells although PLA signals were detected when PSMB6 was stained with a-subunits (Supplementary Fig. 10a, b). Lastly, to examine the association of mitochondria with protein aggregates, we detected protein aggregates and mitochondria. We observed an aggregates-mitochondria association in line with a previous study^53^, and mitochondria colocalized to a greater extent with protein aggregates in *NDUFA11* KO than in WT HEK293T cells (Supplementary Fig. 10c, d). To sum up, EEF1A2 upregulated by mitochondrial stress induces PSMB9 expression, and the induced PSMB9-containing proteasomes in concert with HSPB1 provide an important defense against proteotoxicity in the vicinity of mitochondria.

## Discussion

Cellular proteostasis is challenged by mitochondrial dysfunction characterized by the deficiency in protein import, and appropriate stress responses are required to sustain proteome balance. In this study, we found that a mitochondrial protein import defect caused by *NDUFA11* and *NDUFA13* knockouts results in the accumulation of mitochondrial proteins in the cytosol, and this stress triggers PSMB9 and HSPB1 upregulation. We found that PSMB9, the immunoproteasome-specific subunit acting in addition to the HSPB1 chaperone, is responsible for the maintenance of cellular proteostasis disrupted by mitochondrial dysfunction. PSMB9 induction was mediated by EEF1A2, and the induced PSMB9-containing proteasomes are likely to be localized near mitochondria, suggesting their distinct role in protein degradation associated with mitochondrial dysfunction.

Cells activate an adaptive pathway termed integrated stress response (ISR) to restore cellular homeostasis under stress conditions. Eukaryotic translation initiation factor 2 alpha (EIF2A) is a core component of the ISR, and its phosphorylation increases the expression of the transcription factors ATF4, CHOP and ATF5, which in turn activate the expression of cytoprotective genes^54,55^. Among these three transcription factors, ATF4 was reported as the main player in the mitochondrial stress response in mammalian cells^45^. In both yeast and human cells, mitochondrial defects cause a decrease in global translation that may act to reduce the burden of newly synthesized proteins, otherwise prone to aggregate^12,21,25,56,57^. Furthermore, in yeast another mechanism involving mitochondrial ROS and the oxidation of ribosomal subunits and translation factors leads to the reduction of cytosolic translation^58^. Thus, the cell activates other pathways in addition to the ISR-ATF4 pathway to inhibit translation and thereby affect cell survival. In the present work, we found a previously unknown adaptive response that upregulates PSMB9 via the translation factor EEF1A2 to reestablish cellular proteostasis impaired by mitochondrial complex I deficiency. In mammals, EEF1A1 is ubiquitously expressed, while EEF1A2 is predominantly expressed only in heart, muscle and brain^47,48^ but EEF1A2 occasionally appears in some cancers^59,60^. In line with our finding, recent proteomics data from the study on the regulation of mitochondrial ROS release by cytosolic peroxiredoxins PRDX1 and PRDX2 showed that EEF1A2 is upregulated in *PRDX2* KO and *PRDX1/2* double KO HEK293 cells^61^, suggesting the association of EEF1A2 with mitochondrial stress. Herein, we revealed that mitochondrial stress-induced EEF1A2 is involved in PSMB9 regulation upon mitochondrial dysfunction. We showed the opposite expression patterns of EEF1A1 and EEF1A2 under mitochondrial stress, and their dual localization in the nucleus and cytoplasm in line with previous findings^50^. However, the interplay and exact functions of these two proteins remain to be studied.

Metastable mitochondrial precursor proteins aggregate in the cytosol and trigger the induction of aggregate-related chaperones upon mitochondrial dysfunction in yeast^13^. In agreement with these findings, we observed the upregulation of the specific molecular chaperones HSPB1 and HSPH1 under mitochondrial stress in human cells. HSPB1 and HSPH1 act as holdases preventing protein aggregation under stress conditions^26,29,62,63^. In addition, we found significantly enriched HSPB1 and 20 S proteasome subunits in aggregates isolated from *NDUFA11* KO and *NDUFA13* KO, and in purified proteasome fractions of *NDUFA11* KO compared to those of WT HEK293T cells, reflecting its physical interaction with the proteasome. It was previously reported that HSPB1 directly interacts with the proteasome and promotes proteasomal degradation under stress conditions^64^. Based on the previous study, our data suggest that HSPB1 may assist proteasomes in efficient protein degradation upon mitochondrial dysfunction. In *C. elegans*, enriched small HSPs and proteasome subunits were observed in insoluble protein fractions isolated from of long-lived mutant^31^, indicating that age-related stress responses could be associated with a decline of mitochondrial function. Collectively, our study suggests that HSPB1 is required not only to prevent protein aggregation, but also to facilitate the transfer of aggregation-prone proteins to the proteasome for degradation under mitochondrial stress conditions.

Consistent with previous findings in yeast and *C. elegans*^21,22^, our study showed that mitochondrial deficiency enhances proteasome activity in human cells. Importantly, in this study we define the mechanism by which the activity of the proteasome is increased upon mitochondrial dysfunction. The mechanism involves a more efficient assembly of the 20 S proteasomes, with the inclusion of the immunoproteasome-specific subunit PSMB9 in the 20 S and 26 S proteasomes, followed by recruitment of the PSMB9-containing proteasome complexes to mitochondria. However, this mitochondrial recruitment of PSMB9 does not exclude other localizations of PSMB9-containing proteasomes. The 20 S proteasome preferentially binds to the hydrophobic regions of oxidized and damaged proteins and degrade them in an ATP-independent manner^19,41,42^. Therefore, higher levels of the 20 S proteasome could be beneficial to cope with proteotoxic stress under ATP-depleting conditions such as mitochondrial dysfunction. The main function of the immunoproteasome was initially attributed to generating peptides for major histocompatibility complex class I antigen presentation^39,65^. However, growing evidence shows that the immunoproteasome preferentially degrades oxidized proteins with an activity and selectivity equal to, or greater than, those of the standard proteasome^42,46^. In addition, structural differences in the constitutive proteasome and the immunoproteasome were reported to aid the understanding of various cleavage specificities of β subunits. For instance, PSMB9 preferentially cleaves after hydrophobic residues, while PSMB6 preferentially cleaves after acidic residues^66^. Thus, PSMB9 induction and spatial regulation of the proteasome complexes containing PSMB9 enable more efficient clearance of unfolded or misfolded proteins under mitochondrial stress, thus preventing proteotoxicity.

In conclusion, we identified a novel mechanism that promotes efficient local protein degradation by enhancing proteasome activity through the change in its composition and its potential spatial regulation. The described proteasome remodeling is accompanied with an increase in proteasome interaction with stress-induced HSPB1 which further increases the capacity to avoid aggregation upon mitochondrial dysfunction to keep cellular proteostasis in balance. Our findings contribute to a better understanding of the processes underlying the maintenance of proteostasis upon mitochondrial stress, and thus will help to develop therapies to prevent or delay the onset of age-related neurodegenerative diseases associated with protein homeostasis imbalance.

## Methods

### Cell lines and culture conditions

HEK293T WT and two complex I-deficient cell lines *NDUFA11*−2 KO and *NDUFA13*−2 KO (*NDUFA11* KO and *NDUFA13* KO, respectively), reported in Stroud et al.^23^, were kindly gifted from Dr. Michael T. Ryan from Monash Biomedicine Discovery Institute, Monash University, 3800, Melbourne, Australia. The cells were cultured in Dulbecco’s modified Eagle’s medium (DMEM) with high-glucose content (4500 mg/L) supplemented with 10% (v/v) fetal bovine serum (FBS), 2 mM L-glutamine, 1% (v/v) penicillin-streptomycin and 50 µg/ml uridine at 37 °C in a 5% CO_2_ incubator. The medium was changed every other day for the maintenance of cell lines in culture and 1 day before harvest. To assess ubiquitin accumulation, cells were treated with 500 nM MG132 (Enzo Life Sciences, cat. no. BML-PI102-0005) for 24 h. To induce mitochondrial stress, cells were treated with 100 nM rotenone (Sigma, cat. no. R8875), 10uM menadione (Sigma, cat. no. M5625) and 10 uM CCCP (Sigma, cat. no. C2759) for 2 and 24 h. Cells added with equal volumes of DMSO were set up as control. 1 mM or 5mM N-Acetylcysteine (NAC) (Sigma, cat. no. A9165) was used to prevent ROS production. Immortalized skin fibroblasts from a patient (mt8987i) with a missense homozygous c.221 G > A mutation in exon 2 of the *COX6B1* gene that causes substitution of highly conserved arginine with histidine at position 19 of the mature protein (R19H), and a healthy donor were a kind gift from Prof. Massimo Zeviani laboratory, Mitochondrial Biology Unit, MRC, Cambridge. All the fibroblasts were received with acceptance of BioBank of Telethon Italy located at the Fondazione Istituto Neurologico Carlo Besta, Milan, Italy (original source bank of cells). Immortalized skin fibroblasts were cultured at 37 °C with 5% CO_2_ in DMEM with high-glucose content (4500 mg/L) supplemented with 10% (v/v) fetal bovine serum (FBS), 2 mM L-glutamine, 1% (v/v) penicillin-streptomycin, 1 mM sodium pyruvate and 50 µg/ml uridine. For the experiment, cells were seeded on 100 mm plates at density of 27,000/cm^2^ in DMEM that contained low glucose (1100 mg/L) for 24 h and after this time the medium was changed for DMEM that contained galactose (1800 mg/L) and cells were grown in this condition for the next 48 h.

### Quantitative real-time PCR

Total RNA was isolated with RNeasy Plus Mini Kit (Qiagen, cat. no. 74134) according to the manufacturer’s instructions. 1 ug total RNA was used to generate cDNA using SuperScript™ IV First-Strand Synthesis System (Thermo Fisher Scientific, cat. no. 18091050). To measure mRNA stability, cells were treated with 5 µg/ml Actinomycin D (Sigma, cat. no. A9415) to inhibit transcription. Cells added with equal volumes of DMSO were set up as control. After 0, 4, 8, 16 and 24 h of Actinomycin D treatment, 1 ug total RNA was extracted and cDNA was synthesized using Maxima First Strand cDNA Synthesis Kit for RT-qPCR, with dsDNase (Thermo Fisher Scientific, cat. no. K1671). RT-qPCR was performed using SensiFAST™ SYBR® Hi-ROX Kit (Bioline, cat. no. BIO-92020) in a 96-well white plate (Roche, cat. no. 4729692001) using a LightCycler480 (Roche) with two technical repeats per experimental condition. Fold changes in mRNA expression of the target genes were calculated using the ΔΔCt method. The expression levels of ACTB were used as internal controls. At least two biological replicates were used in the experiment. The exact number of biological replicates of each experiment is stated in the corresponding figure legend. The primers that were used in this study are shown in Supplementary Table 1.

### RNA-seq sample preperation

Total RNA was isolated with RNeasy Plus Mini Kit (Qiagen, cat. no. 74134) followed by DNase I (Sigma, cat. no. AMPD1) treatment. Twelve total RNA samples were subjected to mRNA enrichment using mRNA NEBNext® Poly(A) mRNA Magnetic Isolation Module (New England Biolabs, cat. no. E7490L), from which libraries were derived. Libraries were constructed with KAPA RNA HyperPrep Kit (Kapa Biosciences, cat. no. 08098107702) and KAPA Dual-Indexed adapter kit (Kapa Biosciences, cat. no. KK8722), following manufacturer’s standard protocols, with 2 μg RNA as input, 5 minutes (min) fragmentation in 94 °C and 11 cycles of amplification. The quality of obtained libraries were analyzed using Agilent Bioanalyzer 2100 and High Sensitivity DNA kit (Agilent, cat. no. 5067-4626). Finally, the quantity was measured by qPCR with Kapa Library Quantification kit (Kapa Biosciences, cat. no. KK4824), according to the manufacturer’s instructions. Sequencing was performed on Illumina NovaSeq 6000 using NovaSeq 6000 S1 Reagent Kit (200 cycles) (Illumina, cat. no. 20012864), with pair-end mode of 2 × 100 cycles and standard operating procedure. Four biological replicates were used in the experiment.

### RNA-seq analysis

Raw sequences were trimmed according to quality using Trimmomatic^67^ (version 0.39) using default parameters, except MINLEN, which was set to 50. Trimmed sequences were mapped to human reference genome provided by ENSEMBL (version grch38_snp_tran) using Hisat2^68^ with default parameters. Optical duplicates were removed using MarkDuplicates tool from GATK^69^ (version 4.1.2.0) with default parameters except OPTICAL_DUPLICATE_PIXEL_DISTANCE set to 12000. Reads that failed to map to the reference were extracted using Samtools^70^ and mapped to Silva meta-database of rRNA sequences^71^ (version 119) with Sortmerna^72^ (version 2.1b) using “–best 1” option. Mapped reads were associated with transcripts from GRCh38 database^73^ (Ensembl, version 77) using HTSeq-count^74^ (version 0.9.1) with default parameters except –stranded set to “reverse”. Differentially expressed genes were selected using DESeq2 package^75^ (version 1.16.1). Fold change was corrected using apeglm^76^. Only genes with a maximum Benjamini-Hochberg corrected *p* value (*q*-value) of 0.05 with at least combined mean of 32 reads were deemed to be significantly differentially expressed. RNA sequencing data have been deposited in GEO under accession codes GSE196068. For lists of genes identified and quantified in *NDUFA11* KO versus WT and *NDUFA13* KO versus WT experiments, see Supplementary Data 1.

### Reactome pathway enrichment analysis

Reactome pathways enrichment analysis was performed using R Biocunductor package ReactomePA version 1.36^77^. The calculations were done with enrichedPathway() function using default argument values - minimal size of genes annotated by Ontology term for testing was set to 10, *p*-value cutoff was set to 0.05 and *q*-value cutoff for 0.2. *p*-values were adjusted for multiple testing using FDR controlling method of Benjamini and Hochberg^78^.

### siRNA-mediated knockdown

To silence endogenous *PSMB6*, *PSMB9* or *HSPB1* expression, ON-TARGETplus SMARTpool siRNAs targeting PSMB6 (Dharmacon, cat. no. L-006020-00-0020), *PSMB9* (Dharmacon, cat. no. L-006023-00-0050), *HSPB1* (Dharmacon, cat.no. L-005269-00-0050) were used; ON-TARGETplus Non-targeting siRNA (Dharmacon, cat. no. D-001810-10-50) was used as a negative control. To silence endogenous *EEF1A1*, *EEF1A2* or *HSF1* expression, Silencer™ Pre-Designed siRNAs targeting EEF1A1 (Ambion, ID: 2991), EEF1A2 (Ambion, ID: 10789) or *HSF1* (sense strand: 5’- CGGAUUCAGGGAAGCAGCUGGUGCA-3’, Sigma) were used; Silencer™ Negative Control No. 1 siRNA (Ambion, cat. no.AM4635) was used as a negative control. Cells were reverse-transfected with 20 nM of siRNA using Lipofectamine™ RNAiMAX Transfection Reagent (Thermo Fisher Scientific, cat. no. 13778150) diluted in Opti-MEM™ I Reduced Serum Medium (Thermo Fisher Scientific, cat. no. 11058021) according to the manufacturer’s instructions. Three days after transfection, cells were collected for subsequent analysis.

### Plasmids and transfection

In order to obtain the mitoRUSH plasmid sequences for fusion reporter proteins were generated by sequential PCRs of segments encoding for N-terminal (1-29 aa) presequence of human *COX8* (pre*COX8*), enhanced YFP (EYFP) and streptavidin binding peptide (SBP) using pEYFP-Mito (Clontech) and Ii-streptavidin encoding RUSH plasmid as templates^34^. Sequences were cloned into Ii-streptavidin encoding RUSH plasmid downstream from the IRES sequence using EcoRI and XbaI restriction sites. To generate an expression vector for C-terminal FLAG-tagged *PSMA5*, cDNA fragment containing the entire protein encoding ORF of PSMA5 was amplified with a forward primer containing a EcoRI site (5’-TTTT GAATTC ATG TTT CTT ACC CGG TCT GAG TAC GAC-3’, Sigma) and a reverse primer containing sequences specifying an FLAG tag, a stop codon, and an EcoRI site (5′-TTTT GAATTC TTA CTT ATC GTC GTC ATC CTT GTA ATC AAT GTC CTT GAT AAC CTC TTC AAG-3’, Sigma). The PCR products were digested with EcoRI restriction enzyme and cloned into the EcoRI-digested pcDNA3.1/Zeo(+) vector. Cloned construct was sequenced for insert verification. Cells were transfected with GeneJuice® Transfection Reagent (Merck Millipore, cat. no. 70967) according to the manufacturer’s instructions. Control cells were transfected with empty pcDNA3.1/Zeo(+). Three days after transfection, cells were collected for subsequent analysis. To generate an expression vector for *PSMB9*, cDNA fragment containing the entire protein encoding ORF of *PSMB9* was amplified with a forward primer containing a EcoRI site (5’-TTTT GAATTC ATG CTG CGG GCG GGA GCA CCA AC-3’, Sigma) and a reverse primer containing sequences specifying a stop codon, and an XbaI site (5′-TTTT TCTAGA TCA CTC ATC ATA GAA TTT TGG CA-3’, Sigma). The PCR products were digested with EcoRI and Xba1 restriction enzymes and cloned into the EcoRI and Xba1-digested pcDNA3.1/Zeo(+) vector. Cloned construct was sequenced for insert verification. cells were transfected with GeneJuice® Transfection Reagent (Merck Millipore, cat. no.70967) according to the manufacturer’s instructions. Control cells were transfected with empty pcDNA3.1/Zeo(+). Two days after transfection, cells were collected for subsequent analysis. Expression plasmid containing Myc-DDK-tagged *NDUFA11* cDNA was purchased from Origene (cat. no. RC208966).

### *In organello* import

The coding region of precursors was cloned into a pTNT vector under the SP6 promotor. The recombinant plasmid was used to synthesize S^35^ radiolabelled precursors in TNT SP6 Quick Coupled Transcription/Translation system (Promega). Freshly isolated mitochondria were used for import experiments. Mitochondria were mixed with import buffer (250 mM sucrose, 80 mM potassium acetate, 5 mM magnesium acetate, 5 mM methionine, 10 mM sodium succinate, 20 mM HEPES-KOH pH 7.4) supplemented with fresh 5 mM ATP and heated for 2 min at 24 °C. After incubation, the import was instigated by adding radiolabeled precursor to import buffer containing mitochondria (120 µg), and 30 µg mitochondria were aliquoted at specified time points. A combination of 0.1 mM Valinomycin, 1 mM Oligomycin and 0.8 mM Antimycin A to dissipate membrane potential. After import, proteinase K was administered for 10 min at 4 °C followed by addition PMSF (2 mM) to inactivate the Proteinase K. Further, the samples were centrifuged (20,000 × g, 10 min, 4 °C). The mitochondrial pellet was centrifuged again after being rinsed with high sucrose solution (500 mM sucrose, 20 mM HEPES/KOH, pH 7.4) containing 2 mM PMSF. Finally, the mitochondria pellet containing imported radioactive precursor proteins was solubilized in 2x Laemmli sample buffer (containing 2 mM PMSF and 50 mM DTT). Densitometry of autoradiography images obtained by Typhoon FLA 9500 was used to calculate protein import using the ImageQuant TL application. Samples were analyzed by reducing SDS-PAGE and autoradiography. Three biological replicates were used in the experiment.

### mitoRUSH

WT, *NDUFA11* KO, *NDUFA13* KO HEK293T cells were co-transfected with the mitoRUSH plasmid and a plasmid encoding Ii-streptavidin in proportion (1: 2, w/w) 24 h prior to harvesting. Transfection was performed using GeneJuice Transfection Reagent according to manufacturer protocol. In order to release the reporter 40 μM biotin was added to the medium and cells were incubated at 37 °C with 5% CO_2_ for the indicated time before harvesting. Where indicated 10 μM CCCP was added to the medium together with the biotin.

### Western blot analysis

Cells were lysed in RIPA buffer (65 mM Tris base pH 7.4, 150 mM NaCl, 1% (v/v) NP-40, 0.25% sodium deoxycholate, 1 mM EDTA and 2 mM phenylmethylsulfonyl fluoride (PMSF) for 30 min at 4 °C. The lysate was clarified by centrifugation at 14,000 × g for 10 min at 4 °C. The supernatant was collected and the protein concentration was measured by the Bradford protein assay. The supernatant was diluted in Laemmli buffer that contained 50 mM dithiothreitol (DTT) and denatured at 65 °C for 15 min. Total protein extracts were resolved on 8% or 15% SDS-PAGE gels, transferred to PVDF membranes (Sigma, cat. no. GE10600021) blocked with 5% skim milk (1% BSA for detection of ubiquitination) and probed with specific primary and secondary antibodies, and developed by standard techniques (chemiluminescence) using OPTIMAX2010 or Amersham Imager 600 RGB. The primary antibodies in 5% skim milk (1% BSA for anti-ubiquitin antibody) used for immunoblotting were as follows: GFP (Roche, cat. no. 11814460001, 1:500), HSPB1 (Abcam, cat. no. ab2790, 1:500), HSPA1A/HSP1AB (Enzo Life Sciences, cat. no. ADI-SPA-812-F, 1:2,000), HSP90 (Abcam, cat. no. ab13495, 1:1,000), HSPH1 (Abcam, cat. no. ab109624, 1:500), ACTB (Sigma, cat. no. A1978, 1:2,000), NDUFA11 (Abcam, cat. no. ab183707, 1:500), NDUFA13 (Abcam, cat. no. ab110240, 1:500), PSMB5 (Enzo Life Sciences, cat. no. BML-PW8895-0100, 1:500), PSMB6 (Abcam, cat. no. ab150392, 1:500), PSMB8 (Abcam, cat. no. ab3329, 1:500), PSMB9 (Abcam, cat. no. ab3328, 1:500), PSMA1 (Abcam, cat. no. ab3325, 1:500), PSMD1 (Abcam, cat. no. ab2941, 1:4,000), Proteasome 20 S alpha 1 + 2 + 3 + 5 + 6 + 7 antibody (Abcam, cat. no. ab22674, 1:1,000), Ubiquitin (Santa cruz, cat. no. sc-8017, 1:500), EEF1A1 (Proteintech, cat. no. 11402-1-AP, 1:1,000), EEF1A2 (Thermo Fisher Scientific, cat. no. PA5-27677, 1:500), FLAG (Sigma, cat. no. F1804, 1:500), UQCRC1 (Sigma, cat. no. HPA002815, 1:500), COX5B (Santa scuz, cat. no. sc-374417, 1:500), GAPDH (Santa Cruz, cat. no. sc-47724, 1:500), NDUFS1 (Santa cruz, cat. no. sc-50132, 1:1000), TUBA1C (Santa cruz, cat. no. sc-134239, 1:1000). The secondary horseradish peroxidase-conjugated anti-mouse (Sigma, cat. no. A4416, 1:5000 or 1:10,000), anti-rabbit (Sigma, cat. no. A6154, 1:5000 or 1:10,000), and anti-goat (Sigma, cat. no. A5420, 1:10,000) were used. At least two biological replicates were used in the experiment. The exact number of biological replicates of each experiment is stated in the corresponding figure legend. Uncropped western blot images included in Figures and Supplementary Figures are provided in the Source Data file and the Supplementary Information, respectively.

### Isolation of protein aggregates

Cells were resuspended in lysis buffer (30 mM Tris-HCl pH 7.4, 20 mM KCl, 150 mM NaCl, 5 mM EDTA, 1% Triton X-100 and 2 mM PMSF). After incubation on ice for 30 min (with vortexing every 10 min for 10 sec), samples were shortly centrifuged at a low speed (4 °C, 1000 × g, 1 min) to remove cellular debris. The supernatant was collected and the protein concentration was determined using a Bradford protein assay. 500 ug protein were further centrifuged at a high speed (4 °C, 125,000 × g, 1 h) and separated in soluble and pellet fractions.

### Western blot analysis for detection of ubiquitination and PSMA1 in protein aggregates

After isolation of protein aggregates, pellets were resuspended in 1x urea buffer with 50 mM DTT and heated at 37 °C until they were fully resuspended. Total and soluble fractions were precipitated with TCA (1/10 volume of 100% TCA to the sample), incubated on ice 30 min and centrifuged 20,000 × g for 15 min at 40 °C. The precipitated proteins were washed with ice-cold acetone and centrifuged 20,000 × g for 15 min at 4 °C. The precipitated proteins from total and soluble fractions were resuspended in fresh 1x urea buffer with 50 mM DTT and incubated at 37 °C for 15 min. Equal amount of proteins from each total cell extracts, and an equivalent volume of soluble fractions and/or protein aggregates with each total cell extracts resuspended in 1x urea buffer were used for subsequent electrophoresis. At least two biological replicates were used in the experiment. The exact number of biological replicates of each experiment is stated in the corresponding figure legend.

### Proteasome in-solution activity assay and native gel electrophoresis of proteasome complexes

Cells were lysed in proteasome lysis buffer (50 mM Tris–HCl, pH 7.4, 10 mM MgCl_2_, 250 mM sucrose, 0.5 mM EDTA, 2 mM ATP, 1 mM DTT and 2 mM PMSF) and homogenized in a dounce glass homogenizer. The homogenate was clarified by centrifugation at 10,000 × g for 15 min at 4 °C, and protein concentration was quantified using the Bradford protein assay. 4 ug protein were incubated with 50 uM Suc–Leu–Leu–Val–Tyr–AMC peptide substrate (chymotrypsin-like activity; Bachem, cat. no. I-1395) or 50 uM Ac–NIe–Pro–NIe–Asp–AMC (caspase-like activity; Bachem, cat. no. I-1850) and 10 ug protein were incubated with 50 uM Ac-Pro-Ala-Leu-AMC (Cayman Chemical, cat. no. 26592) to measure the activity of PSMB9 in a final volume of 200 ul of lysis buffer in a 96-well plate. Fluorescence (excitation wavelength 380 nm, emission wavelength 460 nm) was measured every 5 min for 2 h at 25 °C using Synergy H1 Hybrid Multi-Mode Microplate Reader (BioTek, cat. no. H1MFDG). The rate of kinetic reaction (slope) of proteasome activity was calculated and data are represented in a fold change compared to wild-type cells. For native gel electrophoresis, 50 ug of proteins were separated on 4.5% native polyacrylamide gel that was supplemented with 5 mM MgCl_2_, 2.5% sucrose and 1 mM ATP. Electrophoresis was performed at 4 °C for 3.5 h at 30 min in running buffer (90 mM Tris base, 90 mM boric acid, 0.5 mM EDTA pH 8.0, 5 mM MgCl_2_ and 1 mM ATP) according to the well-established protocol^79^ with minor modifications. At least two biological replicates were used in the experiment. The exact number of biological replicates of each experiment is stated in the corresponding figure legend.

### Immunopurification of proteasomes

Cells transfected with C-terminal FLAG-tagged PSMA5 expression plasmid or empty vector were lysed in proteasome lysis buffer and protein concentration was determined as described above. 8 mg protein were incubated with ANTI-FLAG® M2 Affinity Gel (Sigma, cat.no. A2220) for 2 h on a rotator at 4 °C. Immunoprecipitates were washed three times with wash buffer (20 mM Tris-HCl pH 7.4, 150 mM NaCl, 1 mM EDTA and 2 mM PMSF) and eluted with 1 mg/ml FLAG® Peptide (Sigma, cat. no. F3290) in wash buffer overnight on a rotator at 4 °C for analysis by mass spectrometry.

### ROS measurements

The cells were incubated on 6 cm dishes for 20 min at 37 °C in a CO_2_ incubator in a culture medium that contained 10 μM general oxidative stress indicator CM-H2DCFDA dye (Thermo Fisher Scientific, cat. no. C6827). Cells were then collected, washed twice, and resuspended in cold PBS at 4 °C. Fluorescent signals were corrected for autofluorescence. Fluorescence was measured at an excitation wavelength of 495 nm and emission wavelength of 527 nm using a fluorescence microplate reader (Synergy H1 Hybrid Multi-Mode Microplate Reader, BioTek). Three biological replicates were used in the experiment.

### Subcellular fractionation

The cells were harvested, washed twice in ice-cold PBS and resuspended in homogenizing buffer (20 mM HEPES-KOH, pH 7.5, 10 mM KCl, 1.5 mM MgCl_2_, 1 mM sodium EDTA, 1 mM sodium EGTA and 1 mM DTT) containing 250 mM sucrose and 1 mM phenylmethylsulfonyl fluoride (PMSF). After incubation for 30 min on ice, the cells were homogenized using a syringe and needle (10 strokes), and centrifuged at 500 g for 5 min at 4 °C. The pellet was homogenized in buffer C (20 mM HEPES, pH 7.9, 25% glycerol, 0,42 M NaCl, 1,5 mM MgCl_2_, 0,2 mM EDTA and freshly added 0,5 mM PMSF and 0,5 mM DTT) and centrifuged at 20,000 g for 30 min at 4 °C to obtain the nuclear fraction. On the other hand, the supernatant was centrifuged at 10,000 g for 20 min at 4 °C. The resulting pellet was washed three times with homogenizing buffer and solubilized in TNC buffer (10 mM Tris-acetate, pH 8.0, 0.5% NP40, and 5 mM CaCl_2_) to obtain the mitochondrial fraction. The resulting supernatant was centrifuged at 100,000 g for 1 h at 4 °C to obtain the cytoplasmic fraction. Equivalent volumes of the fractions were subjected to western blotting.

### Preparation of aggregate samples for proteomic analysis

Cells were resuspended in lysis buffer (30 mM Tris-HCl pH 7.4, 20 mM KCl, 150 mM NaCl, 5 mM EDTA, 1% Triton X-100 and 2 mM PMSF). After incubation on ice for 30 min (with vortexing every 10 min for 10 sec), samples were shortly centrifuged at a low speed (4 °C, 1000 × g, 1 min) to remove cellular debris. The supernatant was collected and the protein concentration was determined using a Bradford protein assay. 500 ug protein were further centrifuged at a high speed (4 °C, 125,000 × g, 1 h) and separated in soluble and pellet fractions. Isolated protein aggregate fraction was solubilized for 1 h at room temperature with the use of 10 µl 0.1 % ProteaseMAX™ surfactant (Promega, cat. no. V2072) in 50 mM NH_3_HCO_3_. Then, 50 µl of digestion mixture (50 mM Tris-Cl pH 8.2, 5 mM TCEP, 10 mM iodoacetamide, 0.5 µg of sequencing grade modified trypsin and 0.02 % ProteaseMAX™ surfactant (both from Promega) were added and the sample incubated overnight at 37 °C with shaking (850 rpm). Next, the sample was acidified with trifluoroacetic acid (TFA) to a final concentration of 1 % and centrifuged at 12,000 × g for 3 min at room temperature. The peptides in the supernatant were desalted with the use of AttractSPE™ Disks Bio – C18 (Affinisep, cat. no. SPE-Disks-Bio-C18-100.T1.47.20), and dried using a Savant SpeedVac concentrator. Prior to LC-MS measurement, the samples were resuspended in 0.1 % TFA, 2% acetonitrile in water. Three biological replicates were used in the experiment.

### LC-MS/MS analysis of peptide samples derived from protein aggregates

Chromatographic separation was performed on an Easy-Spray Acclaim PepMap column 15 cm long × 75 µm inner diameter (Thermo Fisher Scientific) at 35 °C by applying a 90 min acetonitrile gradients in 0.1% aqueous formic acid at a flow rate of 300 nl/min. An UltiMate 3000 nano-LC system was coupled to a Q Exactive HF-X mass spectrometer via an easy-spray source (all Thermo Fisher Scientific). The Q Exactive HF-X was operated in data-dependent mode with survey scans acquired at a resolution of 60,000 at m/z 200. Up to 12 of the most abundant isotope patterns with charges 2-6 from the survey scan were selected with an isolation window of 1.3 m/z and fragmented by higher-energy collision dissociation (HCD) with normalized collision energies of 27, while the dynamic exclusion was set to 30 s. The maximum ion injection times for the survey scan and the MS/MS scans (acquired with a resolution of 15,000 at m/z 200) were 45 and 22 ms, respectively. The ion target value for MS was set to 3e6 and for MS/MS to 10e5, and the minimum AGC target was set to 8e2.

### Proteomics data processing of protein aggregates

The data were processed with MaxQuant v. 1.6.7.0^80^, and the peptides were identified from the MS/MS spectra searched against Uniprot KB Human Proteome using the build-in Andromeda search engine. Cysteine carbamidomethylation was set as a fixed modification and methionine oxidation as well as protein N-terminal acetylation were set as variable modifications. For in silico digests of the reference proteome, cleavages of arginine or lysine followed by any amino acid were allowed (trypsin/P), and up to two missed cleavages were allowed. The FDR was set to 0.01 for peptides, proteins and sites. Match between runs was enabled. Other parameters were used as pre-set in the software. Unique and razor peptides were used for quantification enabling protein grouping (razor peptides are the peptides uniquely assigned to protein groups and not to individual proteins).

### LFQ-based differential analysis of aggregated protein levels

LFQ values for protein groups were loaded into Perseus v. 1.6.6.0^81^. Standard filtering steps were applied to clean up the dataset: reverse (matched to decoy database), only identified by site, and potential contaminant (from a list of commonly occurring contaminants included in MaxQuant) protein groups were removed. LFQ intensities were log2 transformed and protein groups with log2 LFQ values for at least 2 samples in at least one experimental group were kept. Protein groups with log2 LFQ values for at least 2 samples in both experimental groups (*NDUFA11* KO HEK293T and WT HEK293T) were kept, while protein groups with log2 LFQ values for at least 2 samples in one experimental group and log2 LFQ value for exactly 1 sample in the other group were discarded. For protein groups with log2 LFQ values for at least 2 samples in one experimental group and no log2 LFQ values in the other, data imputation from normal distribution (width = 0.3, down shift = 1.8 × standard deviation) was performed within the experimental group with 0 values. Gaussian distribution of log2 LFQ intensities were confirmed by histogram analysis preventing the unbiased introduction of small values. Student’s *t*-test (two-sided, permutation-based FDR = 0.05, S0 = 0.1) was performed to return proteins which levels were statistically significantly changed in response to *NDUFA11* KO. For the list of proteins identified and quantified in aggregates isolated from *NDUFA11* KO versus WT, see Supplementary Data 2. The mass spectrometry data for protein aggregates have been deposited to the ProteomeXchange Consortium^82^ via the PRIDE^83^ partner repository with the dataset identifier PXD031374.

### Preparation of total cell extracts for proteomic analysis

Cells were resuspended in lysis buffer (30 mM Tris-HCl pH 7.4, 20 mM KCl, 150 mM NaCl, 5 mM EDTA, 1% Triton X-100 and 2 mM PMSF). After incubation on ice for 30 min (with vortexing every 10 min for 10 sec), samples were shortly centrifuged at a low speed (4 °C, 1000 × g, 1 min) to remove cellular debris. The supernatant was collected and the protein concentration was determined using a Bradford protein assay. 100 µg protein per sample was precipitated with chloroform/methanol, resuspended in 100 mM HEPES pH 8.0 containing 5 mM TCEP and 10 mM chloroacetamide, and subjected to overnight digestion at 37 °C with sequencing grade modified trypsin (1 µg). Next, the samples were acidified with trifluoroacetic acid to the final concentration of 1 % and centrifuged at 12,000 × g for 3 min at RT. Tryptic peptides (10 µg per sample) were then desalted with the use of AttractSPE™ Disks Bio C18 (Affinisep), TMT-labeled on the solid support^84^, compiled into a single TMT sample and concentrated using a SpeedVac concentrator. Note: into the same cumulative TMT sample were compiled additional six samples (labelled with orthogonal TMT channels), these were sub-fractions of these very same total cell extracts that are however not discussed in this paper. Peptides in the compiled sample were fractionated (8 fractions) using the Pierce High pH Reversed-Phase Peptide Fractionation Kit (Thermo Fisher Scientific) according to the manufacturer’s instructions. Prior to LC-MS measurement, the peptide fractions were resuspended in 0.1% TFA, 2% acetonitrile in water. Three biological replicates were used in the experiment.

### LC-MS/MS analysis of peptide samples derived from total cell extracts

Chromatographic separation was performed on an Easy-Spray Acclaim PepMap column 50 cm long × 75 µm inner diameter (Thermo Fisher Scientific) at 55 °C by applying a 120 min acetonitrile gradients in 0.1% aqueous formic acid at a flow rate of 300 nl/min. An UltiMate 3000 nano-LC system was coupled to a Q Exactive HF-X mass spectrometer via an easy-spray source (all Thermo Fisher Scientific). The Q Exactive HF-X was operated in TMT mode with survey scans acquired at a resolution of 60,000 at m/z 200. Up to 15 of the most abundant isotope patterns with charges 2-5 from the survey scan were selected with an isolation window of 0.7 m/z and fragmented by higher-energy collision dissociation (HCD) with normalized collision energies of 32, while the dynamic exclusion was set to 35 s. The maximum ion injection times for the survey scan and the MS/MS scans (acquired with a resolution of 45,000 at m/z 200) were 50 and 96 ms, respectively. The ion target value for MS was set to 3e6 and for MS/MS to 1e5, and the minimum AGC target was set to 1e3.

### Proteomics data processing of total cell extracts

The data were processed with MaxQuant v. 1.6.17.0^80^, and the peptides were identified from the MS/MS spectra searched against Uniprot Human Reference Proteome (UP000005640) using the build-in Andromeda search engine. Raw files obtained from the LC-MS/MS measurements of 8 tryptic peptide fractions were analyzed together. Reporter ion MS2-based quantification was applied with reporter mass tolerance = 0.003 Da and min. reporter PIF = 0.75. Cysteine carbamidomethylation was set as a fixed modification and methionine oxidation, glutamine/asparagine deamination, as well as protein N-terminal acetylation were set as variable modifications. For in silico digests of the reference proteome, cleavages of arginine or lysine followed by any amino acid were allowed (trypsin/P), and up to two missed cleavages were allowed. The FDR was set to 0.01 for peptides, proteins and sites. Match between runs was enabled. Other parameters were used as pre-set in the software. Unique and razor peptides were used for quantification enabling protein grouping (razor peptides are the peptides uniquely assigned to protein groups and not to individual proteins).

### TMT-based differential analysis of protein levels in total cell extracts

Reporter intensity corrected values for protein groups were loaded into Perseus v. 1.6.10.0^81^. Standard filtering steps were applied to clean up the dataset: reverse (matched to decoy database), only identified by site, and potential contaminant (from a list of commonly occurring contaminants included in MaxQuant) protein groups were removed. Reporter intensity corrected values were log2 transformed and protein groups with all values were kept. Reporter intensity values were then normalized by median subtraction within TMT channels. Student’s *t*-test (two-sided, permutation-based FDR = 0.01, S0 = 0.1) was performed on the dataset to return protein groups, which levels were statistically significantly changed in NDUFA11 KO vs WT samples. For lists of proteins identified and quantified in *NDUFA11* KO versus WT total cell extracts, see Supplementary Data 2. This dataset has been deposited to the ProteomeXchange Consortium^82^ via the PRIDE^83^ partner repository with the dataset identifier PXD038397.

### Mitochondria Isolation

8 × 10^6^ cells were seeded on 150 mm dishes containing DMEM – high glucose medium and grown for 24 h. After harvesting the cells using a scrapper, the cells were washed with 1X PBS and pelleted by centrifugation (1000 × g, 5 min, 4 °C). The cell pellet was resuspended in an ice-cold isotonic buffer (75 mM mannitol, 225 mM sucrose, 1 mM EGTA 10 mM MOPS-KOH, pH 7.2) containing 2 mM PMSF and centrifuged (1000 × g, 5 min, 4 °C). Afterwards, the cell pellet was resuspended in hypotonic buffer (100 mM sucrose, 1 mM EGTA 10 mM MOPS-KOH, pH 7.2; 5 ml per 1 g of the pellet) containing BSA (2 mg/ml) and 2 mM PMSF and incubated on ice for 5–7 min. The cell suspension was homogenized 15 times using a Dounce glass homogenizer (Sartorius, Catalogue no. BBI-8540705). Cold hypertonic buffer (1.25 M sucrose, 10 mM MOPS-KOH, pH 7.2) was added to the cell homogenate suspension (1.1 ml/1 g cells), and the volume was doubled with isotonic buffer containing BSA (2 mg/ml) and PMSF (2 mM). Then the homogenate suspension was centrifuged (1000 × g, 10 min, 4 °C). The supernatant containing mitochondria was transferred to a fresh falcon tube and centrifugated again to pellet the debris. The supernatant was subjected to high-speed centrifugation (10,000 × g, 10 min, 4 °C) to pellet mitochondria. This method of mitochondria isolation is adopted from Panov^85^.

### Segregation of soluble fraction (post aggregate pelleting) and aggregates

After pelleting mitochondria, the supernatant were subjected to lysis buffer (30 mM Tris-HCl pH 7.4, 20 mM KCl, 150 mM NaCl, 5 mM EDTA, 1% Triton X-100 and 2 mM PMSF as final concentration) to solubilize the samples. Then, the samples were incubated for 30 mins with occasional vortexing. The soluble samples were centrifuged (10,000 × g, 10 min, 4 °C) to remove the debris. Ice cold isotonic buffer without BSA and PMSF was used to wash the pellet and centrifuged again. Then, the pellet was resuspended in isotonic buffer (without BSA and PMSF) and the soluble samples were subjected to Bradford assay for measuring protein concentration. Finally, 500 µg of soluble sample was subjected high speed centrifugation (125,000 × g, 60 min, 4 °C) to segregate aggregates (pellet) and soluble fraction (post aggregate pelleting).

### Preparation of mitochondria for proteomic analysis

50 µg protein per sample of isolated mitochondria were dissolved in neat trifluoroacetic acid. Protein solutions were neutralized with 10 volumes of 2 M Tris base, supplemented with TCEP (8 mM) and chloroacetamide (32 mM), heated to 95 °C for 5 min, diluted with water 1:5, and subjected to overnight enzymatic digestion (0.5 µg, Sequencing Grade Modified Trypsin, Promega) at 37 °C. Tryptic peptides were then desalted with the use of AttractSPE™ Disks Bio C18 (Affinisep), TMT-labeled on the solid support^84^, compiled into a single TMT sample and concentrated using a SpeedVac concentrator. Peptides in the compiled sample were fractionated (into 8 fractions) using the Pierce High pH Reversed-Phase Peptide Fractionation Kit (Thermo Fisher Scientific) according to the manufacturer’s instructions (fractions 1, 2, and 3 were pulled). Prior to LC-MS measurement, the peptide fractions were resuspended in 0.1% TFA, 2% acetonitrile in water. Three biological replicates were used in the experiment.

### LC-MS/MS analysis of peptide samples derived from mitochondria

Chromatographic separation was performed on an Easy-Spray Acclaim PepMap column 50 cm long × 75 µm inner diameter (Thermo Fisher Scientific) at 55 °C by applying a 120 min acetonitrile gradients in 0.1% aqueous formic acid at a flow rate of 300 nl/min. An UltiMate 3000 nano-LC system was coupled to a Q Exactive HF-X mass spectrometer via an easy-spray source (all Thermo Fisher Scientific). The Q Exactive HF-X was operated in TMT mode with survey scans acquired at a resolution of 60,000 at m/z 200. Up to 18 of the most abundant isotope patterns with charges 2–5 from the survey scan were selected with an isolation window of 0.7 m/z and fragmented by higher-energy collision dissociation (HCD) with normalized collision energies of 32, while the dynamic exclusion was set to 35 s. The maximum ion injection times for the survey scan and the MS/MS scans (acquired with a resolution of 30,000 at m/z 200) were 50 and 96 ms, respectively. The ion target value for MS was set to 3e6 and for MS/MS to 1e5, and the minimum AGC target was set to 1e3.

### Proteomics data processing of mitochondria

The data were processed with MaxQuant v. 1.6.17.0^80^, and the peptides were identified from the MS/MS spectra searched against Uniprot Human Reference Proteome (UP000005640) using the build-in Andromeda search engine. Raw files obtained from the LC-MS/MS measurements of 6 tryptic peptide fractions were analyzed together. Reporter ion MS2-based quantification was applied with reporter mass tolerance = 0.003 Da and min. reporter PIF = 0.75. Cysteine carbamidomethylation was set as a fixed modification and methionine oxidation, glutamine/asparagine deamination, as well as protein N-terminal acetylation were set as variable modifications. For in silico digests of the reference proteome, cleavages of arginine or lysine followed by any amino acid were allowed (trypsin/P), and up to two missed cleavages were allowed. The FDR was set to 0.01 for peptides, proteins and sites. Match between runs was enabled. Other parameters were used as pre-set in the software. Unique and razor peptides were used for quantification enabling protein grouping (razor peptides are the peptides uniquely assigned to protein groups and not to individual proteins).

### TMT-based differential analysis of protein levels in mitochondria

Reporter intensity corrected values for protein groups were loaded into Perseus v. 1.6.10.0^81^. Standard filtering steps were applied to clean up the dataset: reverse (matched to decoy database), only identified by site, and potential contaminant (from a list of commonly occurring contaminants included in MaxQuant) protein groups were removed. Protein groups matched to the list of high-confidence mitochondrial proteins were kept^86^. Reporter intensity corrected values were log2 transformed and protein groups with all values were kept. Reporter intensity values were then normalized by median subtraction within TMT channels. Student’s *t*-test (two-sided, permutation-based FDR = 0.01, S0 = 0.6) was performed on the dataset to return protein groups, which levels were statistically significantly changed in *NDUFA11* KO vs WT samples. For lists of proteins identified and quantified in the mitochondrial fractions isolated from *NDUFA11* KO versus WT cells, see Supplementary Data 3. This dataset has been deposited to the ProteomeXchange Consortium^82^ via the PRIDE^83^ partner repository with the dataset identifier PXD038004.

### Preparation of the cytoplasmic soluble fractions (post aggregate pelleting) for proteomic analysis

100 µg protein per sample was precipitated with chloroform/methanol, resuspended in 100 mM HEPES pH 8.0 containing 5 mM TCEP and 10 mM chloroacetamide, and subjected to overnight digestion at 37 °C with sequencing grade modified trypsin (1 µg). Next, the samples were acidified with trifluoroacetic acid to the final concentration of 1 % and centrifuged at 12,000 × g for 3 min at RT. Tryptic peptides (10 µg per sample) were then desalted with the use of AttractSPE™ Disks Bio C18 (Affinisep), TMT-labeled on the solid support^84^, compiled into a single TMT sample and concentrated using a SpeedVac concentrator. Peptides in the compiled sample were fractionated (8 fractions) using the Pierce High pH Reversed-Phase Peptide Fractionation Kit (Thermo Fisher Scientific) according to the manufacturer’s instructions. Prior to LC-MS measurement, the peptide fractions were resuspended in 0.1% TFA, 2% acetonitrile in water. Three biological replicates were used in the experiment.

### LC-MS/MS analysis of peptide samples derived from cytoplasmic soluble fractions (post aggregate pelleting)

Chromatographic separation was performed on an Easy-Spray Acclaim PepMap column 50 cm long × 75 µm inner diameter (Thermo Fisher Scientific) at 55 °C by applying a 120 min acetonitrile gradients in 0.1% aqueous formic acid at a flow rate of 300 nl/min. An UltiMate 3000 nano-LC system was coupled to a Q Exactive HF-X mass spectrometer via an easy-spray source (all Thermo Fisher Scientific). The Q Exactive HF-X was operated in TMT mode with survey scans acquired at a resolution of 60,000 at m/z 200. Up to 18 of the most abundant isotope patterns with charges 2-5 from the survey scan were selected with an isolation window of 0.7 m/z and fragmented by higher-energy collision dissociation (HCD) with normalized collision energies of 32, while the dynamic exclusion was set to 35 s. The maximum ion injection times for the survey scan and the MS/MS scans (acquired with a resolution of 30,000 at m/z 200) were 50 and 96 ms, respectively. The ion target value for MS was set to 3e6 and for MS/MS to 1e5, and the minimum AGC target was set to 1e3.

### Proteomics data processing of cytoplasmic soluble fractions (post aggregate pelleting)

The data were processed with MaxQuant v. 1.6.17.0^80^, and the peptides were identified from the MS/MS spectra searched against Uniprot Human Reference Proteome (UP000005640) using the build-in Andromeda search engine. Raw files obtained from the LC-MS/MS measurements of 8 tryptic peptide fractions were analyzed together. Reporter ion MS2-based quantification was applied with reporter mass tolerance = 0.003 Da and min. reporter PIF = 0.75. Cysteine carbamidomethylation was set as a fixed modification and methionine oxidation, glutamine/asparagine deamination, as well as protein N-terminal acetylation were set as variable modifications. For in silico digests of the reference proteome, cleavages of arginine or lysine followed by any amino acid were allowed (trypsin/P), and up to two missed cleavages were allowed. The FDR was set to 0.01 for peptides, proteins and sites. Match between runs was enabled. Other parameters were used as pre-set in the software. Unique and razor peptides were used for quantification enabling protein grouping (razor peptides are the peptides uniquely assigned to protein groups and not to individual proteins).

### TMT-based differential analysis of protein levels in cytoplasmic soluble fractions

Reporter intensity corrected values for protein groups were loaded into Perseus v. 1.6.10.0^81^. Standard filtering steps were applied to clean up the dataset: reverse (matched to decoy database), only identified by site, and potential contaminant (from a list of commonly occurring contaminants included in MaxQuant) protein groups were removed. Protein groups matched to the list of high confidence mitochondrial proteins were kept^86^. Reporter intensity corrected values were log2 transformed and protein groups with all values were kept. Reporter intensity values were then normalized by median subtraction within TMT channels. Student’s *t*-test (two-sided, permutation-based FDR = 0.01, S0 = 0.2) was performed on the dataset to return protein groups, which levels were statistically significantly changed in NDUFA11 KO vs WT samples. For lists of proteins identified and quantified in the soluble fractions isolated from *NDUFA11* KO versus WT cells, see Supplementary Data 3. This dataset has been deposited to the ProteomeXchange Consortium^82^ via the PRIDE^83^ partner repository with the dataset identifier PXD038004.

### Preperation of cytoplasmic protein aggregates for proteomic analysis

Isolated cytoplasmic protein aggregates were solubilized for 1 h in 10 µl of 0.1 % ProteaseMAX™ surfactant (Promega, catalogue no. V2072) in 50 mM ammonium bicarbonate at RT. Then, 50 µl of digestion mixture (100 mM HEPES pH 8.0, 5 mM TCEP, 10 mM iodoacetamide, 0.5 µg of sequencing grade modified trypsin (Promega, catalogue no. V5111) and 0.02 % ProteaseMAX™ surfactant) was added and the samples were digested overnight at 37 °C. Next, the samples were acidified with trifluoroacetic acid to the final concentration of 1 % and centrifuged at 12,000 × g for 3 min at RT. Tryptic peptides (the entire sample) were then desalted with the use of AttractSPE™ Disks Bio C18 (Affinisep), TMT-labeled on the solid support^84^, compiled into a single TMT sample and concentrated using a SpeedVac concentrator. Peptides in the compiled sample were fractionated (8 fractions) using 3-layers of AttractSPE™ Disks Bio C18 (Affinisep) and a gradient (60 µl per fraction) described for the High pH Reversed-Phase Peptide Fractionation Kit (Thermo Fisher Scientific). Fractions were then pulled: Fr1 + 2 + 3; Fr4 + 5 + 6; Fr7 + 8. Prior to LC-MS measurement, the peptide fractions were resuspended in 0.1% TFA, 2% acetonitrile in water. Three biological replicates were used in the experiment.

### LC-MS/MS analysis of peptide samples derived from cytoplasmic protein aggregates

Chromatographic separation was performed on an Easy-Spray Acclaim PepMap column 50 cm long × 75 µm inner diameter (Thermo Fisher Scientific) at 55 °C by applying a 150 min acetonitrile gradients in 0.1% aqueous formic acid at a flow rate of 300 nl/min. An UltiMate 3000 nano-LC system was coupled to a Q Exactive HF-X mass spectrometer via an easy-spray source (all Thermo Fisher Scientific). The Q Exactive HF-X was operated in TMT mode with survey scans acquired at a resolution of 60,000 at m/z 200. Up to 18 of the most abundant isotope patterns with charges 2-5 from the survey scan were selected with an isolation window of 0.7 m/z and fragmented by higher-energy collision dissociation (HCD) with normalized collision energies of 32, while the dynamic exclusion was set to 35 s. The maximum ion injection times for the survey scan and the MS/MS scans (acquired with a resolution of 30,000 at m/z 200) were 50 and 96 ms, respectively. The ion target value for MS was set to 3e6 and for MS/MS to 1e5, and the minimum AGC target was set to 1e3.

### Proteomics data processing of cytoplasmic protein aggregates

The data were processed with MaxQuant v. 1.6.17.0^80^, and the peptides were identified from the MS/MS spectra searched against Uniprot Human Reference Proteome (UP000005640) using the build-in Andromeda search engine. Raw files obtained from the LC-MS/MS measurements of 3 tryptic peptide fractions were analyzed together. Reporter ion MS2-based quantification was applied with reporter mass tolerance = 0.003 Da and min. reporter PIF = 0.75. Cysteine carbamidomethylation was set as a fixed modification and methionine oxidation, glutamine/asparagine deamination, as well as protein N-terminal acetylation were set as variable modifications. For in silico digests of the reference proteome, cleavages of arginine or lysine followed by any amino acid were allowed (trypsin/P), and up to two missed cleavages were allowed. The FDR was set to 0.01 for peptides, proteins and sites. Match between runs was enabled. Other parameters were used as pre-set in the software. Unique and razor peptides were used for quantification enabling protein grouping (razor peptides are the peptides uniquely assigned to protein groups and not to individual proteins).

### TMT-based differential analysis of protein levels in cytoplasmic protein aggregates

Reporter intensity corrected values for protein groups were loaded into Perseus v. 1.6.10.0^81^. Standard filtering steps were applied to clean up the dataset: reverse (matched to decoy database), only identified by site, and potential contaminant (from a list of commonly occurring contaminants included in MaxQuant) protein groups were removed. Protein groups matched to the list of high confidence mitochondrial proteins were kept^86^. Reporter intensity corrected values were log2 transformed and protein groups with all values were kept. Reporter intensity values were then normalized by median subtraction within TMT channels. Student’s *t*-test (two-sided, permutation-based FDR = 0.01, S0 = 0.2) was performed on the dataset to return protein groups, which levels were statistically significantly changed in NDUFA11 KO vs WT samples. For lists of proteins identified and quantified in the aggregates isolated from *NDUFA11* KO versus WT cells, see Supplementary Data 3. This dataset has been deposited to the ProteomeXchange Consortium^82^ via the PRIDE^83^ partner repository with the dataset identifier PXD038004.

### Quantitative MS analysis of proteasome complexes

Proteins of proteasome complexes affinity-purified via FLAG-tagged PSMA5 from *NDUFA11* KO, *NDUFA13* KO or WT (control) cells were acetone-precipitated and resuspended in 6 M urea/50 mM NH_4_HCO_3_, followed by reduction and alkylation of cysteine residues and tryptic in solution digestion as described before^87^. Peptides were differentially labeled by stable isotope dimethyl labeling as described previously^88^ using ‘light’ formaldehyde and sodium cyanoborohydride (CH2O/NaBH3CN) for peptides originating from WT cells and the corresponding deuterated ‘heavy’ versions (CD2O/NaBD3CN) for peptides from *NDUFA11* KO and *NDUFA13 KO* cells. Labeling efficiencies were assessed by liquid chromatography-mass spectrometry (LC-MS). Equal amounts of light and heavy labeled peptides were mixed, desalted on StageTips, and analyzed by LC-MS (*n* = 3 per experiment, i.e. *NDUFA11* KO versus WT and *NDUFA13 KO* versus WT) using either an Orbitrap Elite or a Q Exective Plus mass spectrometer, each connected to an UltiMate 3000 RSLCnano HPLC system. Mass spectrometric raw data were searched against the Uniprot human proteome set including isoforms, to which the sequence for PSMA5-FLAG was added, and a list of common contaminants using MaxQuant/Andromeda (version 1.6.0.1^80,89^). MaxQuant was operated with default settings, except that protein identification and quantification were based on ≥1 unique peptide and ≥ 1 ratio count, respectively. DimethylLys0/dimethylNter0 and dimethylLys6/dimethylNter6 were selected as light and heavy labels, carbamidomethlyation of cysteine residues was set as fixed modification, N-terminal acetylation and oxidation of methionine were considered variable modifications, and ‘match between runs’ and ‘requantify’ were enabled. Protein abundance ratios (i.e. PSMA5 complexes affinity-purified from *NDUFA11* KO or *NDUFA13* KO versus WT cells) determined by MaxQuant were normalized replicate-wise to the respective median value and log_2_ transformed. To evaluate effects of *NDUFA11* KO or *NDUFA13* KO on the composition of the proteasome, the rank product method^90^ as implemented in the R package ‘RankProd'^91^ was applied. Only proteins quantified in all three replicates per experiment were considered for this analysis. Using the ‘RankProd’ package, *p*-values are determined according to Heskes et al.^92^. For lists of proteins identified and quantified in isolated proteasomes from *NDUFA11* KO versus WT and *NDUFA13* KO versus WT experiments, see Supplementary Data 4. The mass spectrometry data for proteasome complexes have been deposited to the ProteomeXchange Consortium^82^ via the PRIDE^83^ partner repository with the dataset identifier PXD031282.

### Immunofluorescence

Cells were seeded on glass coverslips coated with 50 µg/ml poly-D-lysine (Sigma, cat. no. P7280). After 24 h, cells were fixed with 3.7% formaldehyde for 20 min and permeabilized with PBS containing 0.1% Triton X-100 for 10 min. Proximity ligation assay (PLA) was subsequently performed using Duolink® In Situ Detection Reagents Orange (Sigma, DUO92007) according to the manufacturer’s instructions using primary antibodies against PSMB9 (Abcam, cat. no. ab3328, 1:500), PSMB6 (Abcam, cat. no. ab150392, 1:500) and SDHA (Santa Cruz, cat. no. sc-166947, 1:100). For TOMM20 and SDHA co-staining, primary antibodies against TOMM20 (Santa Cruz, cat. no. sc-11415, 1:100) and SDHA (Santa Cruz, cat. no. sc-166947, 1:100) were used followed by Alexa Fluor™ 647 (Invitrogen, cat. no. A-21245, 1:200) and Alexa Fluor™ 488 (Invitrogen, cat. no. A-11029, 1:200), respectively. For mitochondrial co-staining, 500 nM MitoTracker Deep Red (Invitrogen, cat. no. M22426) was added to live cells and the cells were incubated for 30 min at 37 °C. After washing with PBS two times, cells were fixed with 3.7% formaldehyde at 37 °C followed by permeabilization and PLA staining or PROTEOSTAT® staining. To visualize protein aggregates, PROTEOSTAT® Aggresome detection kit (Enzo Life Sciences, cat. no. ENZ-51035) was used following the manufacturer’s instructions. After mounting of the coverslips with ProLong Diamond antifade mountant with DAPI (Thermo Fisher Scientific, P36962) on a microscope slide, images were acquired by a Zeiss LSM700 confocal microscope using 405 nm laser for DAPI, 488 nm laser for protein aggregates, 555 nm laser for PLA signals, and 639 nm laser for MitoTracker Deep Red using a 20× objective up to 80× with a digital zoom. To quantify protein aggregates, the number of cells in the images taken at 20× magnification was automatically counted by ImageJ, and cells with protein aggregates in the same images were manually counted. To quantify PLA signals, the number of cells and PLA signals in the images taken at 80× magnification were manually and automatically counted by ImageJ, respectively. For colocalization analysis, the number of cells in the images taken at 80× magnification was manually counted by ImageJ. Pearson’s correlation and Manders’ coefficients were calculated using the JACoP plugin on ImageJ. Three biological replicates were used in the experiment.

### AHA labeling

WT, *NDUFA11* KO and *NDUFA13* KO HEK293T cells treated with scramble siRNA or siRNA targeting *EEF1A1*/*EEF1A2* were grown on 100 mm cell culture dishes. 3 h before labeling, medium was changed to fresh medium. Before addition of azidehomoalanine (AHA) to a final concentration of 2 mM the dishes were washed two times with PBS to get rid of the residual methionine and incubated for 10 min in methionine free medium. Newly synthesized proteins were labeled for 1 h 50 min with AHA. Following the labeling the cells were washed two times with hot PBS and transferred immediately to ice and detached by gentle pipetting in 1 mL of ice cold PBS. Cell pellets were centrifuged × 1000 g, PBS was aspirated and cell pellets were frozen in liquid nitrogen.

### Staining the AHA labeled nascent proteins with click chemistry compatible fluorescent probe

Frozen cell pellets containing AHA labeled nascent proteins were lysed in 500 µL of click chemistry compatible PBS buffer containing 1.5% SDS, 1% Triton and cOmplete™, Mini, EDTA-free Protease Inhibitor Cocktail. Chromatin was fragmented and cell pellets were dissolved by quick sonic pulses. The almost transparent lysates were centrifuged > 15,000 g for 5 min. Supernatant were concentrated on 4 kDa ultrafiltration columns (amicon ultra – MERCK- Millipore) to decrease free AHA and SDS concentration after diluting the concentrated lysate with 160 µL of PBS buffer containing cOmplete™, Mini, EDTA-free Protease Inhibitor Cocktail. Click reaction of AHA labeled nascent proteins with 5-Carboxytetramethylrhodamine-Alkyne (TAMRA) was performed in 600 µL volume using the following concentrations of reagents: protein lysate ~8 ug/µL; PBS pH 7.4 60%; Tris((1-hydroxy-propyl-1H-1,2,3-triazol-4-yl)methyl)amine (THPTA) 400 uM; CuSO4 1 mM, TAMRA alkyne 400 uM, freshly made Tris (2-carboxyethyl) phosphine hydrochloride (TCEP) 2 mM. The reaction was performed with gentle rotation in room temperature for 1 h 15 min and stopped by addition of EDTA to the final concentration of 5 mM. The fluorescently labeled nascent proteins were methanol/chlorophorm precipitated and dissolved directly in bromophenol free Laemli buffer prior to SDS-PAGE.

### Fluorescent SDS-PAGE quantification

The fluorescent SDS-PAGE signal coming from TAMRA labeled nascent proteins was scanned by Typhoon FLA 9500 prior to staining the total protein in the gels with Coomasie. Both TAMRA and Coomasie raw signals were normalized to signal present in the lane with lowest intensity for each gel individually to enable appropriate statistical analysis of different gels. The individual sample signal was described as ratio between nascent (TAMRA) and total (Coomasie) staining signal and the final signal as ratio of normalized TAMRA/Coomasie signal for control divided by normalized TAMRA/Coomasie signal for treatment. At least two biological replicates were used in the experiment. The exact number of biological replicates of each experiment is stated in the corresponding figure legend.

### Data visualization

All the the visualizations of highthroupout data were produced using programming scripts written in R. The following packages were used to produce volcano plots: ‘ggplot2‘, ‘ggrepel‘. For coding heatmaps ‘pheatmap‘ package was used. PDF figures exported from R studio were assembled in adobe illustrator to form final publication ready figures.

### Statistical analysis

For the statistical analysis, two-sided, unpaired *t*-tests assuming equal or unequal variance using Microsoft Excel 2010 and GraphPad Prism 7.0 were used unless stated otherwise. Values of *p*-value < 0.05 were considered statistically significant.

### Reporting summary

Further information on research design is available in the Nature Portfolio Reporting Summary linked to this article.

## Supplementary information

Supplementary Information

Description of Additional Supplementary Files

Supplementary Data 1

Supplementary Data 2

Supplementary Data 3

Supplementary Data 4

Reporting Summary

## Data Availability

RNA sequencing data have been deposited in GEO under accession codes GSE196068. The mass spectrometry data for protein aggregates have been deposited to the ProteomeXchange Consortium via the PRIDE partner repository with the dataset identifier PXD031374. The mass spectrometry data for total cell extracts have been deposited to the ProteomeXchange Consortium via the PRIDE partner repository with the dataset identifier PXD038397. The mass spectrometry data for mitochondria, cytoplasmic soluble and aggregate fractions have been deposited to the ProteomeXchange Consortium via the PRIDE partner repository with the dataset identifier PXD038004. The mass spectrometry data for proteasome complexes have been deposited to the ProteomeXchange Consortium via the PRIDE partner repository with the dataset identifier PXD031282. Source data are provided with this paper.

## Source data

Source Data
