# Supplementary material for: Immunoproteasome-specific subunit PSMB9 induction is required to regulate cellular proteostasis upon mitochondrial dysfunction
Source: Nat Commun. 2023 Jul 11;14:4092. doi: 10.1038/s41467-023-39642-8 (PMC10336106; doi:10.1038/s41467-023-39642-8)

Supplementary Information

Immunoproteasome-specific subunit PSMB9 induction is required to regulate cellular proteostasis upon mitochondrial dysfunction

Minji Kim[1], Remigiusz A. Serwa[1,2], Lukasz Samluk[3], Ida Suppanz[4,5], Agata Kodroń[1,2], Tomasz M. Stępkowski[1,2], Praveenraj Elancheliyan[1], Biniyam Tsegaye[1], Silke Oeljeklaus[6], Michal Wasilewski[1], Bettina Warscheid[4,6], Agnieszka Chacinska[1,2,*]

[1]IMol Polish Academy of Sciences, Warsaw, Poland

[2]ReMedy International Research Agenda Unit, IMol Polish Academy of Sciences, Warsaw, Poland

[3]Centre of New Technologies, University of Warsaw, Warsaw, Poland

[4]CIBSS Centre for Integrative Biological Signalling Studies, University of Freiburg, Freiburg, Germany

[5]Present address: Max Planck Institute of Immunobiology and Epigenetics, Freiburg, Germany

[6]Department of Biochemistry, Theodor Boveri-Institute, Biocenter, University of Würzburg, Würzburg, Germany

*Correspondence should be directed to: a.chacinska@imol.institute

Contents:

Supplementary Figures 1-10

Supplementary Table 1

Uncropped western blot images included in Supplementary Figures

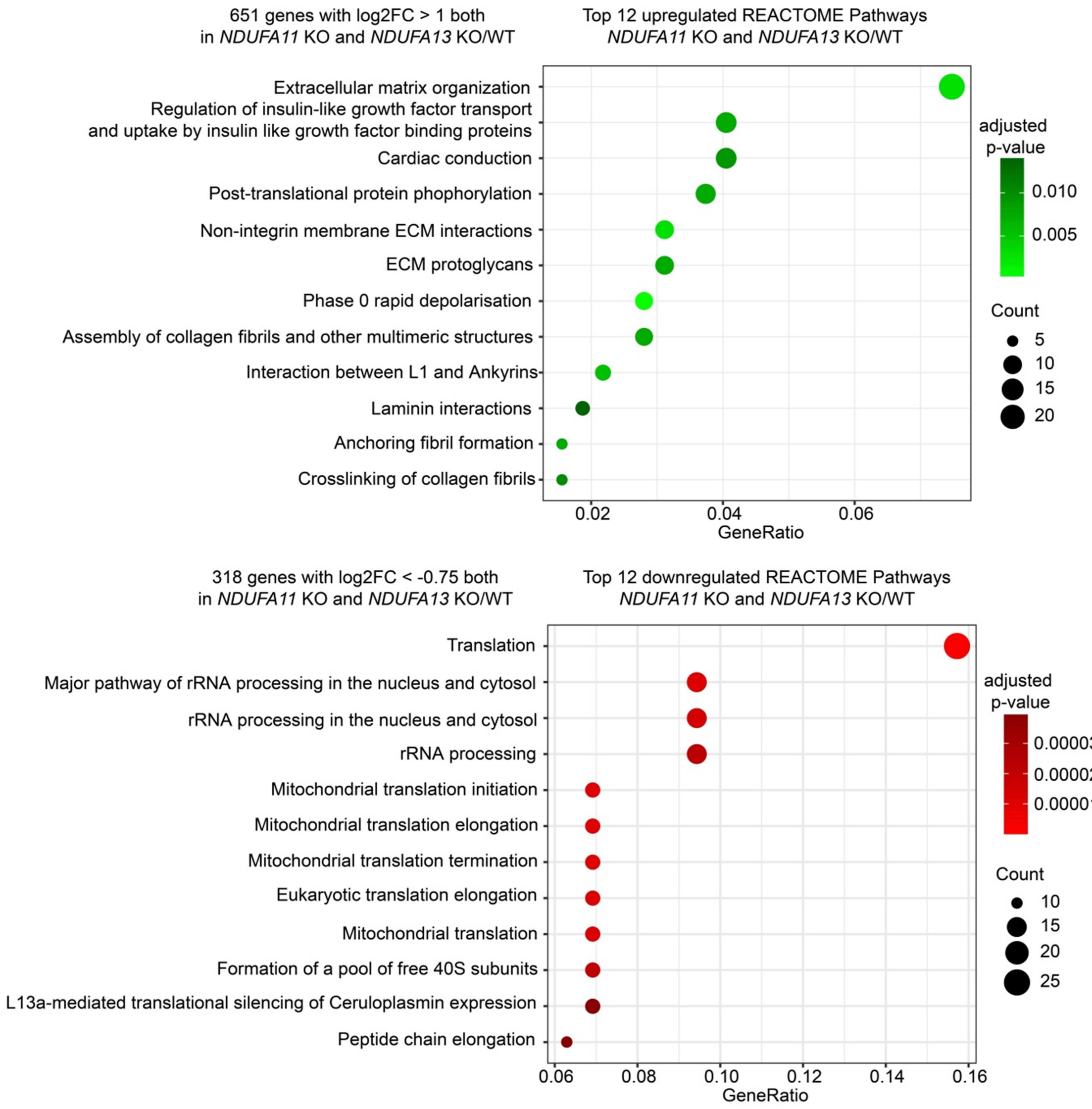

Supplementary Figure 1. Mitochondrial complex I deficiency causes transcriptome changes. Top 12 pathways revealed by Reactome pathway enrichment analysis for genes upregulated with the log2 fold change (log2FC) > 1 and adjusted *p*-value < 0.05 (upper panel, green) and downregulated with the log2 fold change (log2FC) < -0.75 and q-value < 0.05 (lower panel, red) in both *NDUFA11* KO and *NDUFA13* KO compared to WT HEK293T cells (n=4). The R function used to calculate *p*-values is based on hypergeometric test which correspond to one sided Fisher exact test. The obtained *p*-values were adjusted for mutlitple comparisons with Benjamini Hochberg method.

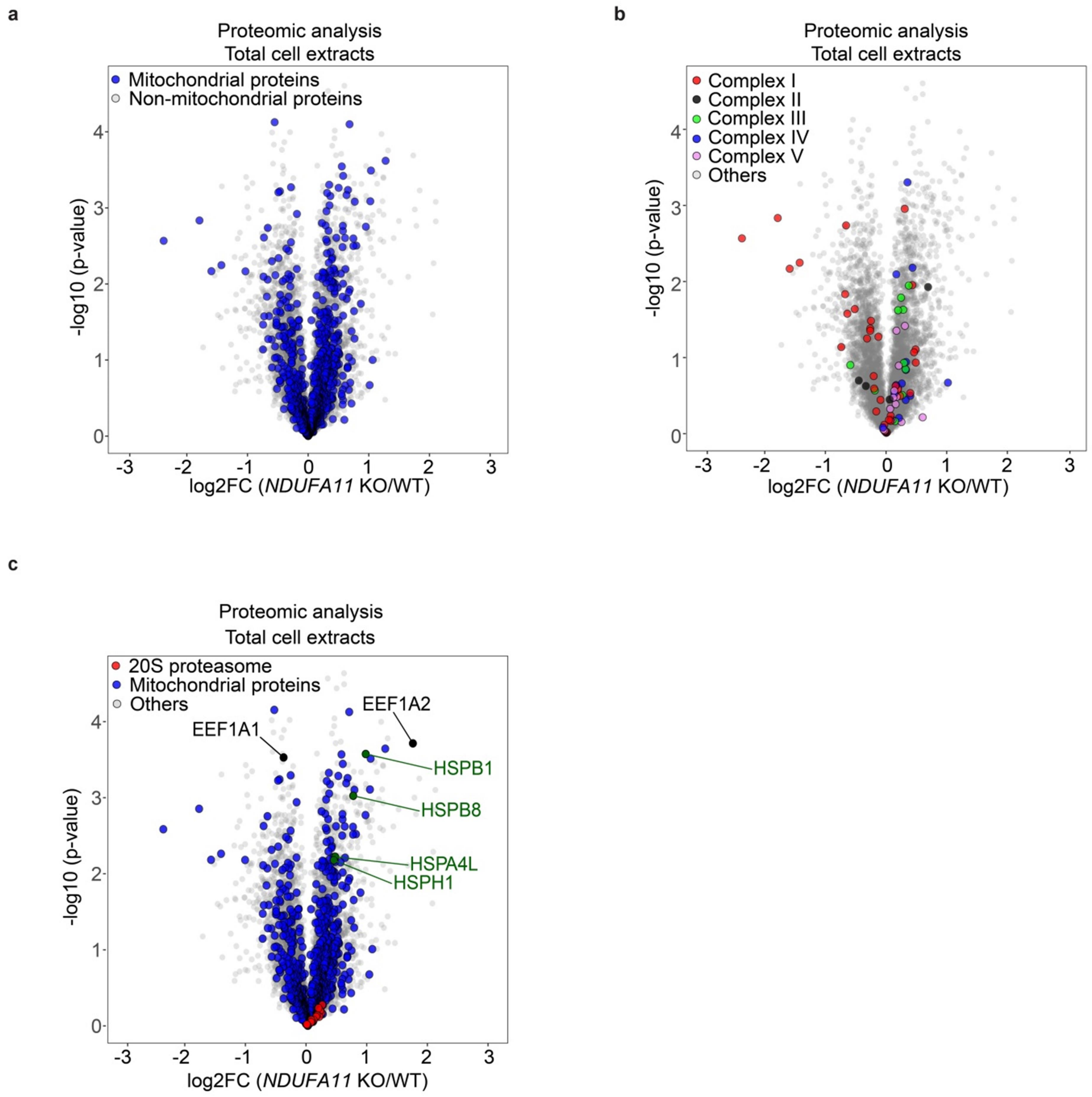

Supplementary Figure 2. Mitochondrial complex I deficiency does not lead to significant changes in mitochondrial protein abundance. (**a-c**) Volcano plot displaying the log2 fold change (log2FC, x axis) against the *t* test-derived −log10 statistical *p*-value (y axis) for all protein groups detected in total cell extracts of *NDUFA11* KO and WT HEK293T cells by LC-MS/MS analysis (n=3). Student's *t*-test (two-sided, unpaired) was performed for the statistical analysis. (**a**) Mitochondrial proteins and non-mitochondrial proteins are indicated as blue and gray dots, respectively. (**b**) Subunits of mitochondrial complex I, II, III, IV, V and others are indicated as red, black, green, blue, pink, and gray dots, respectively. (**c**) 20S proteasome subunits, mitochondrial proteins, and others are indicated as red, blue, and

gray dots, respectively. HSPB1, HSPB8, HSPH1 and HSPA4L are indicated as green dots, EEF1A1 and EEF1A2 are indicated as black dots.

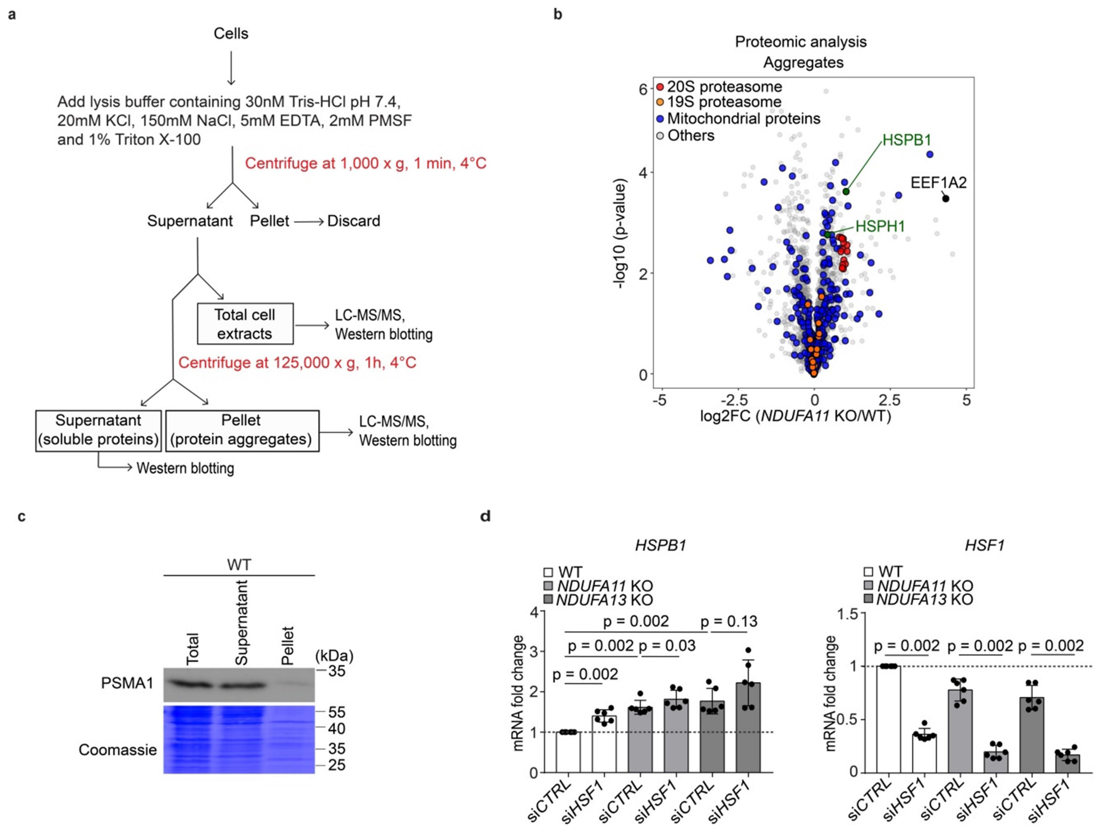

Supplementary Figure 3. **HSPB1, HSPH1 and 20S proteasome subunits are enriched in protein aggregates isolated from *NDUFA11* KO HEK293T cells. (a)** Workflow of protein aggregates isolation for LC-MS/MS analysis. (**b**) Volcano plots displaying the log2 fold change (log2FC, x axis) against the *t* test-derived −log10 statistical *p*-value (y axis) for all protein groups detected in aggregate fractions of *NDUFA11* KO and WT HEK293T cells by LC-MS/MS analysis (n=3). Student's *t*-test (two-sided, unpaired) was performed for the statistical analysis. 20S proteasome subunits, 19S proteasome subunits, mitochondrial proteins, and others are indicated as red, orange, blue, and gray dots, respectively. HSPB1 and HSPH1 are indicated as green dots, EEF1A2 is indicated as a black dot. (**c**) Western blot analysis performed in total cell extracts, supernatants and pellets of WT HEK293T cells. Coomassie Blue staining was used as a loading control. Data shown are representative of three independent experiments. (**d**) mRNA expression levels of *HSPB1* and *HSF1* examined

by RT-qPCR analysis in mitochondrial complex I-deficient and WT HEK293T cells transfected with *HSF1* (si*HSF1*) or control (siCTRL) siRNAs for 72 h. Data shown are mean ± SD (n=3 biological replicates with two technical replicates). *p*-value from two-sided, unpaired Mann Whitney test using GraphPad Prism. Source data are provided as a Source Data file.

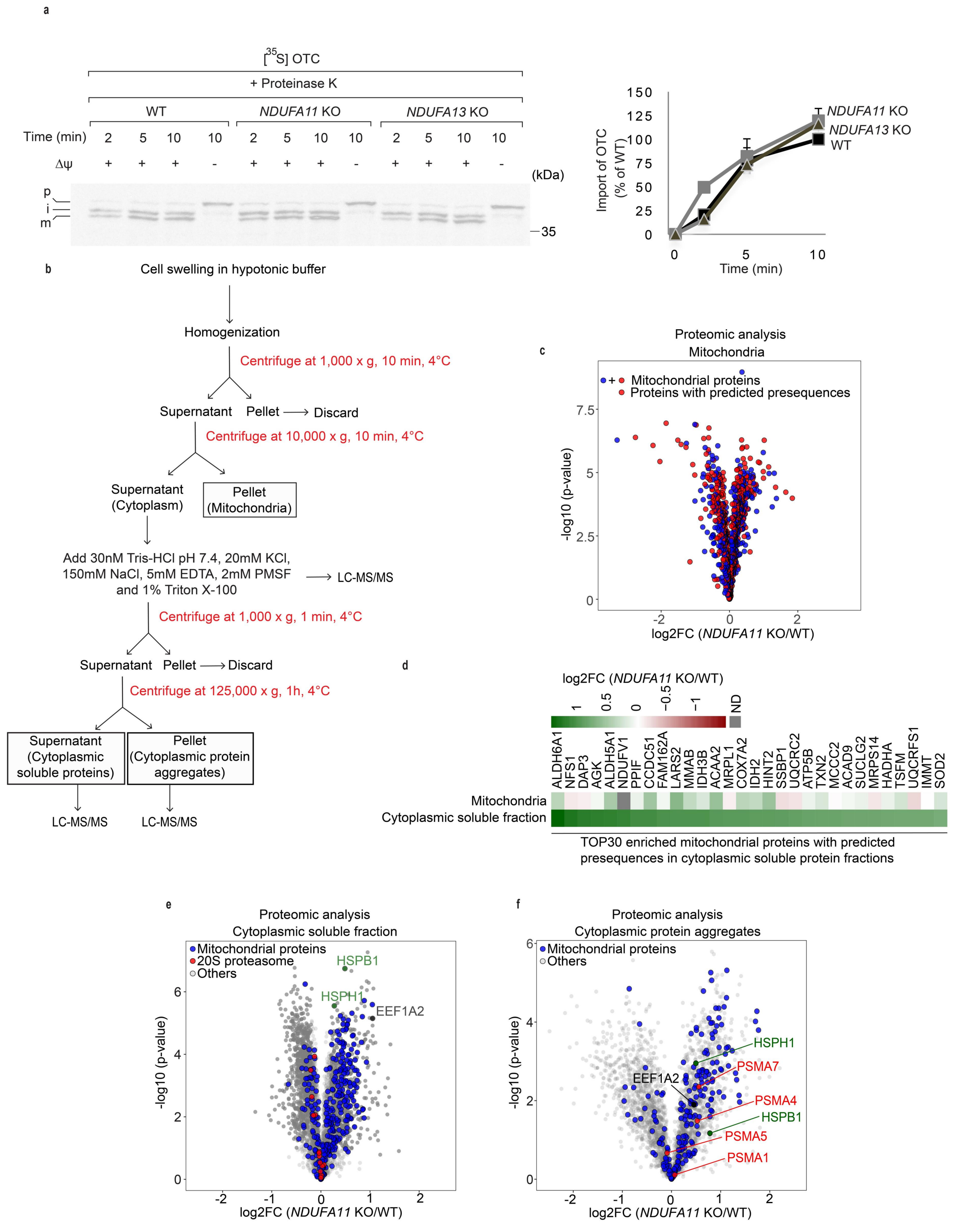

Supplementary Figure 4. Mitochondrial complex I-deficiency leads to accumulation of non-imported mitochondrial proteins in the cytosol where they are aggregated. (**a**) Mitochondrial protein import assay using [^{35}S]-labeled OTC precursors in mitochondrial complex I-deficient cells and WT HEK293T cells. Data shown are representative of three

independent experiments (left panel). Quantified data shown are mean ± SEM (right panel, n=3). p, precursor; i, intermediate; m, mature. (**b**) Workflow of subcellular fractionation and preparation of samples for LC-MS/MS analysis. (**c**) Volcano plot displaying the log2 fold change (log2FC, x axis) against the *t* test-derived −log10 statistical *p*-value (y axis) for high-confidence mitochondrial proteins detected in isolated mitochondria of *NDUFA11* KO and WT HEK293T cells by LC-MS/MS analysis (n=3). Student's *t*-test (two-sided, unpaired) was performed for the statistical analysis. Mitochondrial proteins with or without predicted presequences are indicated as red and blue dots, respectively. (**d**) Heatmap of 30 most highly enriched mitochondrial proteins with predicted presequences in cytoplasmic soluble fractions of *NDUFA11* KO vs WT HEK293T cells compared to their relative abundance in mitochondria isolated from these cells (n=3). The intensity of the color shades depends on the level of expression change. ND, not detected. (**e**) Volcano plot displaying the log2 fold change (log2FC, x axis) against the *t* test-derived −log10 statistical *p*-value (y axis) for all protein groups detected in cytoplasmic soluble fractions of *NDUFA11* KO and WT HEK293T cells by LC-MS/MS analysis (n=3). Student's *t*-test (two-sided, unpaired) was performed for the statistical analysis. Mitochondrial proteins, 20S proteasome subunits, HSPs (HSPB1, HSPH1) and others are indicated as blue, red, green, and gray dots, respectively. (**f**) Volcano plot displaying the log2 fold change (log2FC, x axis) against the *t* test-derived −log10 statistical *p*-value (y axis) for all protein groups detected in cytoplasmic protein aggregates fractions of *NDUFA11* KO and WT HEK293T cells by LC-MS/MS analysis (n=3). Student's *t*-test (two-sided, unpaired) was performed for the statistical analysis. Mitochondrial proteins, 20S proteasome subunits, HPSs (HSPB1, HSPH1) and others are indicated as blue, red, green, and gray dots, respectively. Source data are provided as a Source Data file.

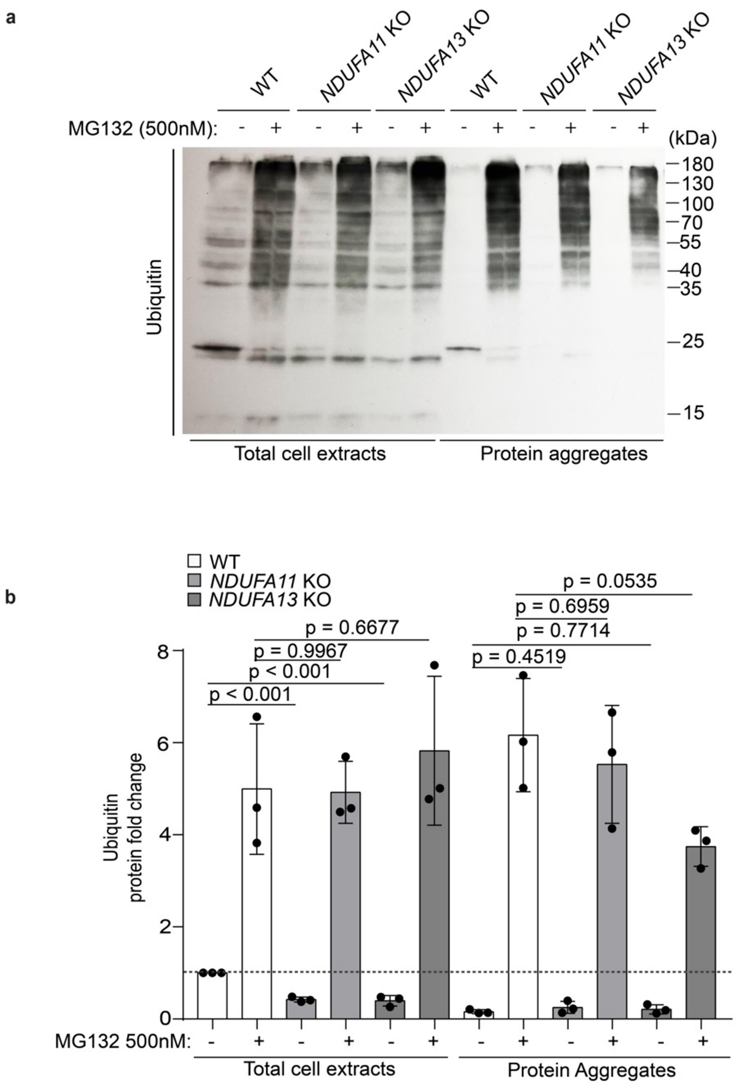

Supplementary Figure 5. The proteasome of mitochondrial complex I-deficient cells has higher capacity to degrade polyubiquitinated proteins than that of WT HEK293T cells. (**a**) Western blot analysis performed in total cell extracts and aggregates of WT and mitochondrial complex I-deficient HEK293T cells after 24 h of MG132 treatment. Data shown are representative of three independent experiments. (**b**) Quantification of ubiquitination in (**a**) using ImageJ. The densitometry of proteins from 25 kDa to 180 kDa for each group was used for quantification. The protein levels are presented as fold changes relative to WT. Data shown are mean ± SD (n=3). *p*-value from an ordinary one-way ANOVA with Dunnett's multiple comparisons test using GraphPad Prism. Source data are provided as a Source Data file.

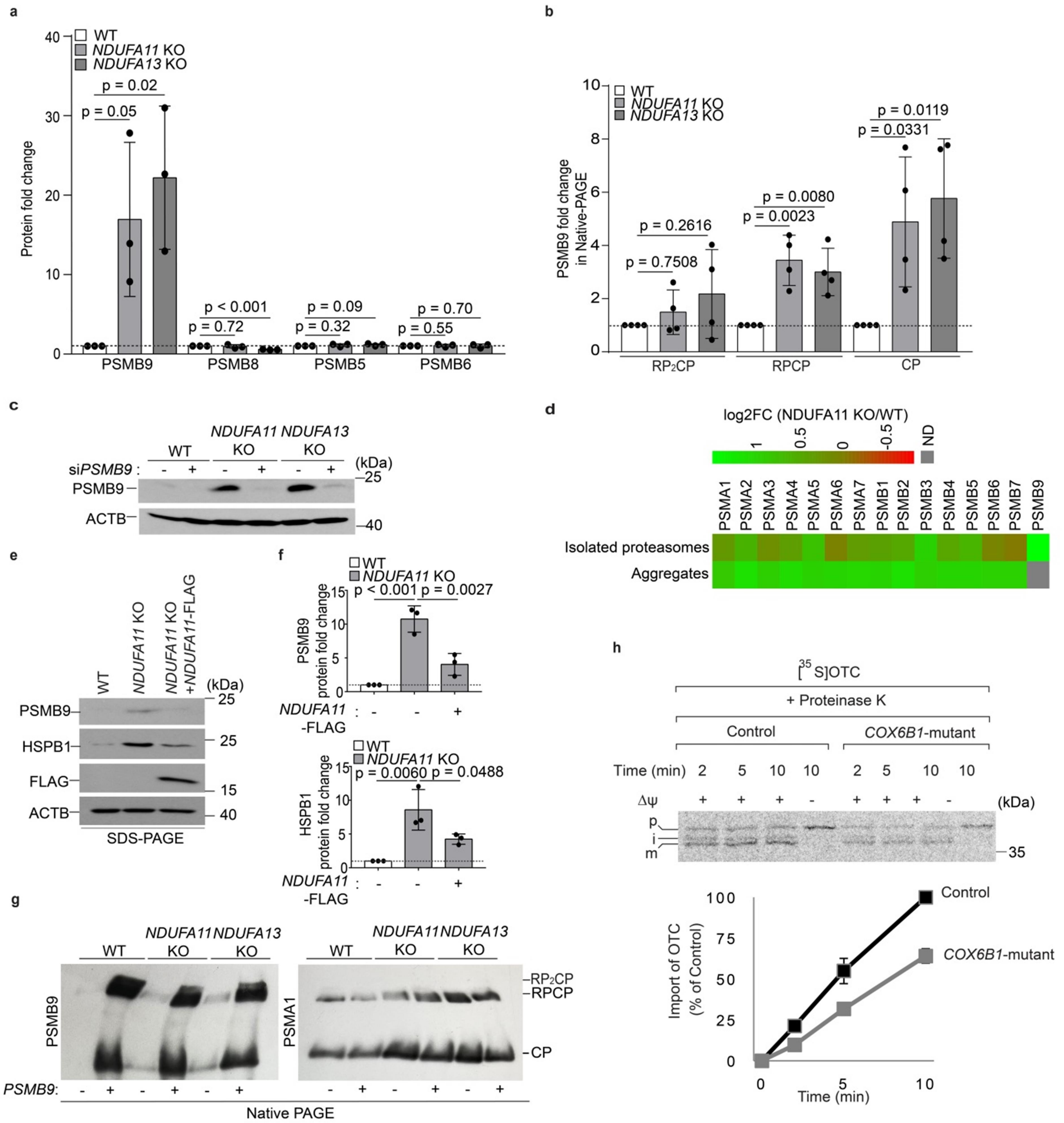

i

Transcriptomic analysis (HeLa), log2FC

	Control	Dox	Acti	FCCP	MB
PSMB9	0	0.10	-0.46	-0.37	1.15
PSMB10	0	0.13	-0.41	-0.29	0.86
PSMB8	0	0.24	0.01	-0.28	0.22
PSMB5	0	-0.33	-0.21	-0.17	-0.09
PSMB6	0	-0.20	0.12	-0.15	-0.04
PSMB7	0	-0.26	0.23	-0.01	0.36
HSPB1	0	-0.34	-1.11	-0.71	-0.13

Proteomic analysis (HeLa), log2FC

	Control	Dox	Acti	FCCP	MB
PSMB9	0	0.34	0.33	0.18	0.56
PSMB10	0	0.21	-0.05	0.01	0.46
PSMB8	0	0.25	0.14	0.04	0.07
PSMB5	0	0.13	0.09	0.07	0.08
PSMB6	0	0.16	0.05	0.06	0.04
PSMB7	0	0.04	0.04	0.06	0.01
HSPB1	0	0.04	-0.03	-0.15	-0.11

0.5 < log2FC

Supplementary Figure 6. **PSMB9 is induced under mitochondrial stress. (a, b)** The protein levels are presented as fold changes relative to WT. *p*-value from an ordinary one-way ANOVA with Dunnett's multiple comparisons test using GraphPad Prism. Data shown are mean ± SD. (**a**) Quantification of β subunits in Fig. 4c normalized to ACTB using ImageJ

(n=3). (**b**) Quantification of PSMB9 in proteasomes in Fig. 4d using ImageJ (n=4). (**c**) Western blotting validation of *PSMB9* knockdown. Data shown are representative of three independent experiments. (**d**) Heatmap of proteasome subunits in isolated proteasomes and in aggregates (n=3). The intensity of the color shades depends on the level of expression change. ND, not detected. (**e**) Western blot analysis performed in whole cell lysates of *NDUFA11* KO and WT HEK293T treated with transfection reagents, and *NDUFA11* KO HEK293T cells transfected with FLAG-tagged *NDUFA11* expression plasmid. Data shown are representative of three independent experiments. (**f**) Quantification of PSMB9 and HSPB1 in (**e**) normalized to ACTB using ImageJ. The protein levels are presented as fold changes relative to WT. Data shown are mean ± SD (n=3). *p*-value from an ordinary one-way ANOVA with Holm-Sidak's multiple comparisons test using GraphPad Prism. (**g**) Proteasome species in *NDUFA11* KO, *NDUFA13* KO and WT HEK293T cell extracts transfected with *PSMB9* expression plasmids resolved in 4.5% native gel followed by western blot analysis detecting PSMB9 and PSMA1 to characterize 26S (RP_2CP, RP_1CP) and 20S (CP) proteasomes. Data shown are representative of two independent experiments. (**h**) Mitochondrial protein import assay using [^{35}S]-labeled OTC precursors in *COX6B1*-mutant and control fibroblasts. Data shown are representative of three independent experiments (upper). Quantified data shown are mean ± SEM (lower, n=3). p, precursor; i, intermediate; m, mature. (**i**) Analysis of published transcriptomic and proteomic data of HeLa cells after treatment of doxycycline (dox), actinonin (acti), fccp or MitoBloCK-6 (mb) (n=2). The log2 fold change (log2FC) of normalized counts from RNA sequencing and normalized TMT signal-to-noise from proteomics data provided in Quiros et al. (2017) were used for the analysis. Upregulated genes and proteins with the log2 fold change (log2FC) > 0.5 are shown in green. Source data are provided as a Source Data file.

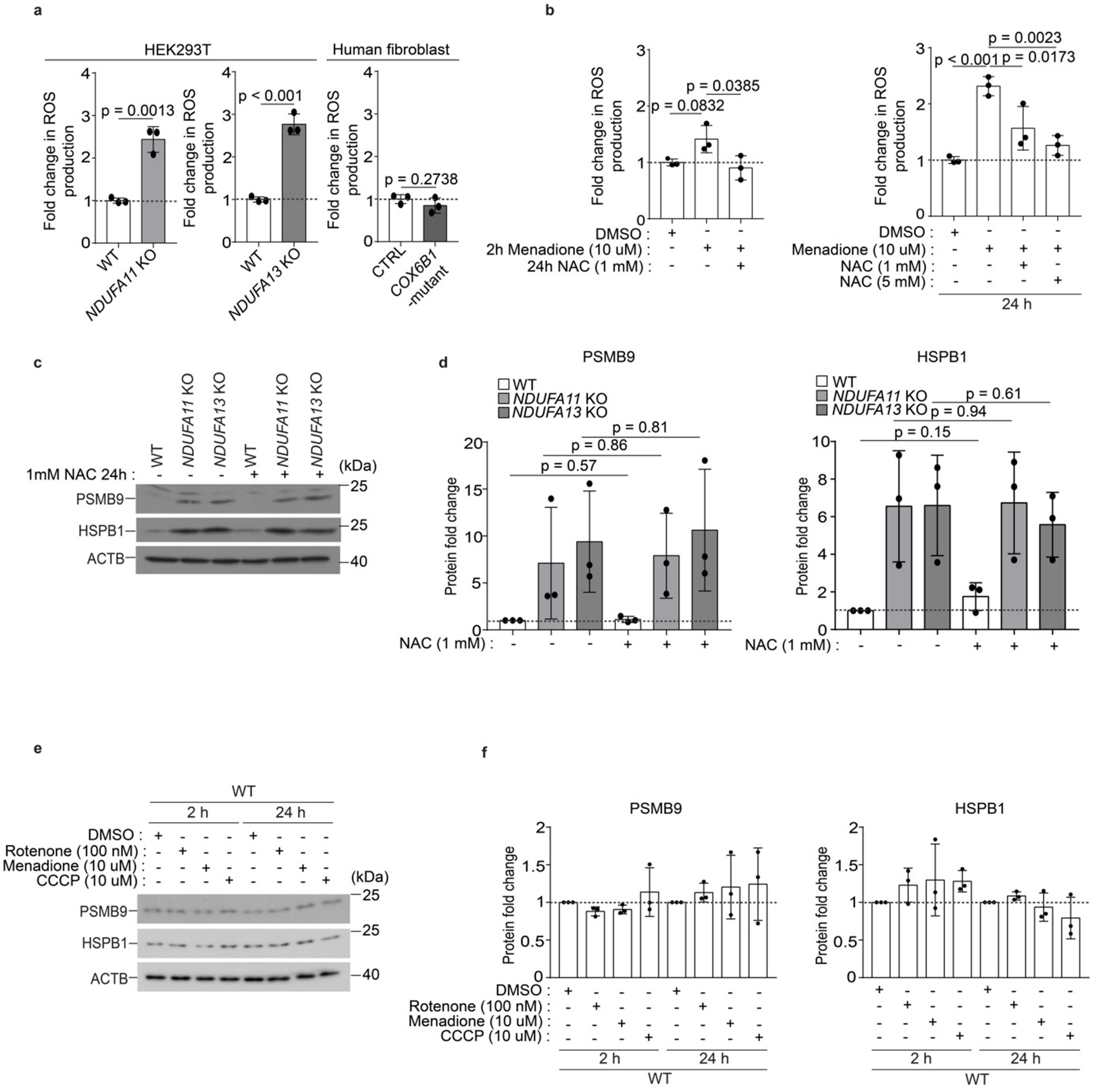

Supplementary Figure 7. Induction of PSMB9 and HSPB1 in *NDUFA11* KO and *NDUFA13* KO is ROS-independent. (**a**) ROS production in mitochondrial complex I-deficient and WT HEK293T cells, *COX6B1*-mutant and control fibroblasts (CTRL). Data shown are mean ± SD (n=3). *p*-value from two-sided, unpaired *t*-test using GraphPad Prism. (**b**) ROS production in HEK293T cells under menadione and/or N-acetyl-L-cysteine (NAC) treatments as indicated. Data shown are mean ± SD (n=3). *p*-value from an ordinary one-way ANOVA with Tukey's multiple comparisons test using GraphPad Prism. (**c**) Western blot analysis performed in whole cell lysates of mitochondrial complex I-deficient and WT HEK293T treated with NAC for 24 h. ACTB was used as a loading control. Data shown are

representative of three independent experiments. (**d**) Quantification of PSMB9 and HSPB1 in (**c**) normalized to ACTB using ImageJ. The protein levels are presented as fold changes relative to WT. Data shown are mean ± SD (n=3). *p*-value from two-sided, unpaired *t*-test using GraphPad Prism. (**e**) Western blot analysis performed in whole cell lysates of WT treated with DMSO, rotenone, menadione or CCCP for 2 and 24 h. ACTB was used as a loading control. Data shown are representative of three independent experiments. (**f**) Quantification of PSMB9 and HSPB1 in (**e**) normalized to ACTB using ImageJ. The protein levels are presented as fold changes relative to WT. Data shown are mean ± SD (n=3). None of them was significantly changed compared to those of WT from two-sided, unpaired *t*-test using GraphPad Prism. Source data are provided as a Source Data file.

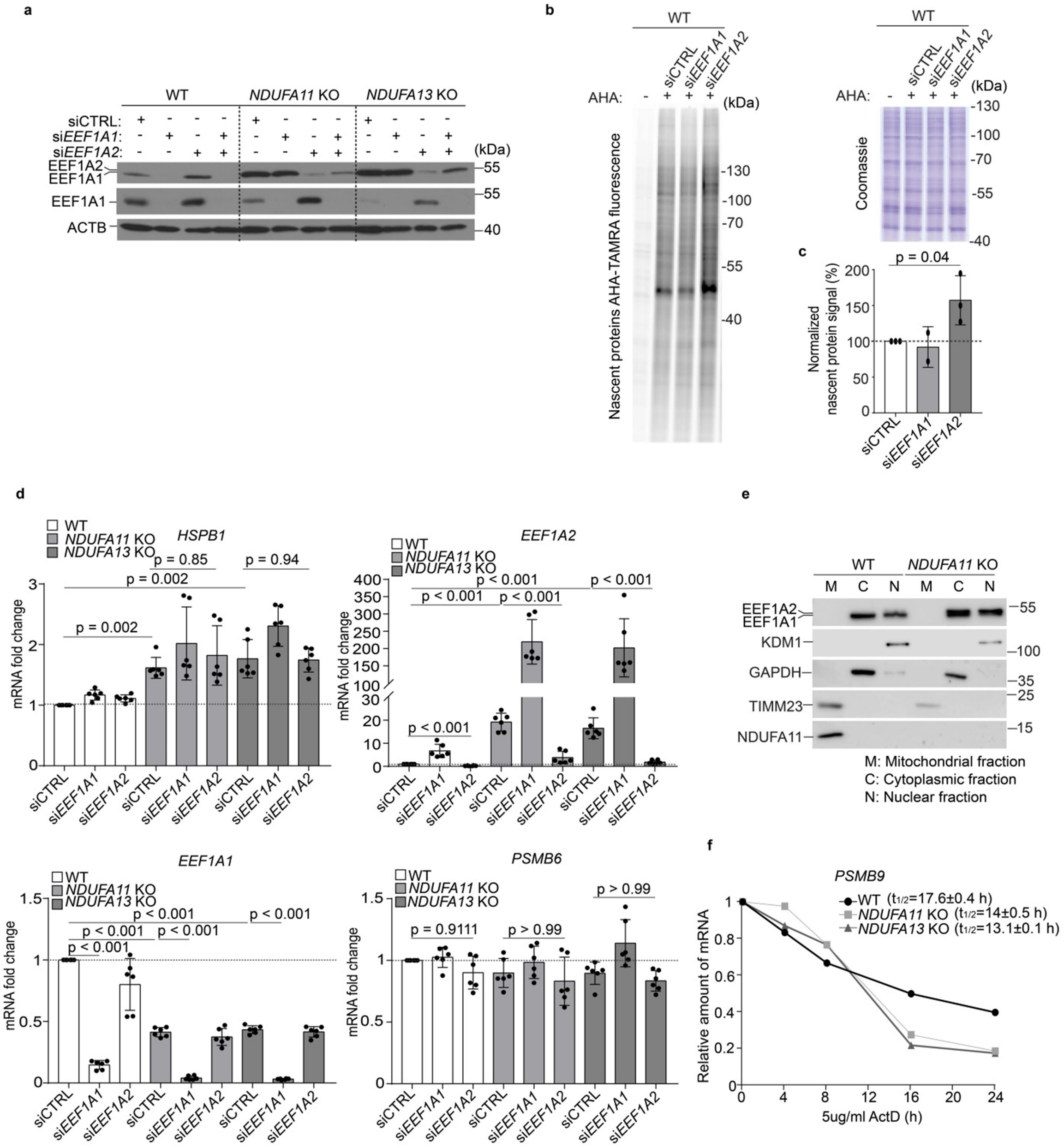

Supplementary Figure 8. EEF1A2 has an inhibitory effect on translation in WT HEK293T cells. (**a**) Western blot analysis of mitochondrial complex I-deficient and WT HEK293T cells treated with siRNA against *EEF1A1* (si*EEF1A1*), *EEF1A2* (si*EEF1A2*) and control siRNA (siCTRL). ACTB was used as a loading control. Anti-EEF1A2 antibody was used in the first blot, anti-EEF1A1 antibody was used in the second blot from the top. Data shown are representative of two independent experiments. (**b**) Fluorescent SDS-PAGE of AHA-TAMRA labeled nascent proteins in WT, NDUFA11KO and NDUFA13KO cells treated

with siRNA against *EEF1A1* (si*EEF1A1*) (n=2), *EEF1A2* (*siEEF1A2) and* control siRNA (siCTRL) (n=3). Coomassie Blue staining was used as a loading control. Data shown are representative of two independent experiments. (**c**) Quantification of fluorescence in (**b**). The nascent protein signals are presented as fold changes relative to WT value. Data shown are mean ± SD. *p*-value from two-sided, unpaired *t*-test using GraphPad Prism. (**d**) *HSPB1*, *EEF1A2*, *EEF1A1* and *PSMB6* mRNA expression patterns examined by RT-qPCR in mitochondrial complex I-deficient and WT HEK293T cells transfected with *EEF1A1* (si*EEF1A1*), *EEF1A2* (si*EEF1A2*) or control (siCTRL) siRNA for 72 h. The mRNA levels are presented as fold changes relative to WT transfected with control siRNA. Data shown are mean ± SD (n=3 biological replicates with two technical replicates). *p*-value from two-sided, unpaired *t*-test or Mann Whitney test using GraphPad Prism. (**e**) Western blot analysis of subcellular fractionation. Anti-EEF1A2 antibody was used in the blot on the top. KDM1, GAPDH and TIMM23 were used as controls of the nuclear, cytoplasmic, and mitochondrial fraction, respectively. Data shown are representative of five independent experiments. (**f**) PSMB9 mRNA stability assay performed by RT-qPCR after 0, 4, 8, 16 and 24h Actinomycin D treatment in *NDUFA11* KO and WT HEK293T cells. Data are presented as fold changes of mRNA levels relative to DMSO-treated control at each time point and are mean ± SD (n=2). $t_{1/2}$, half-life. Source data are provided as a Source Data file.

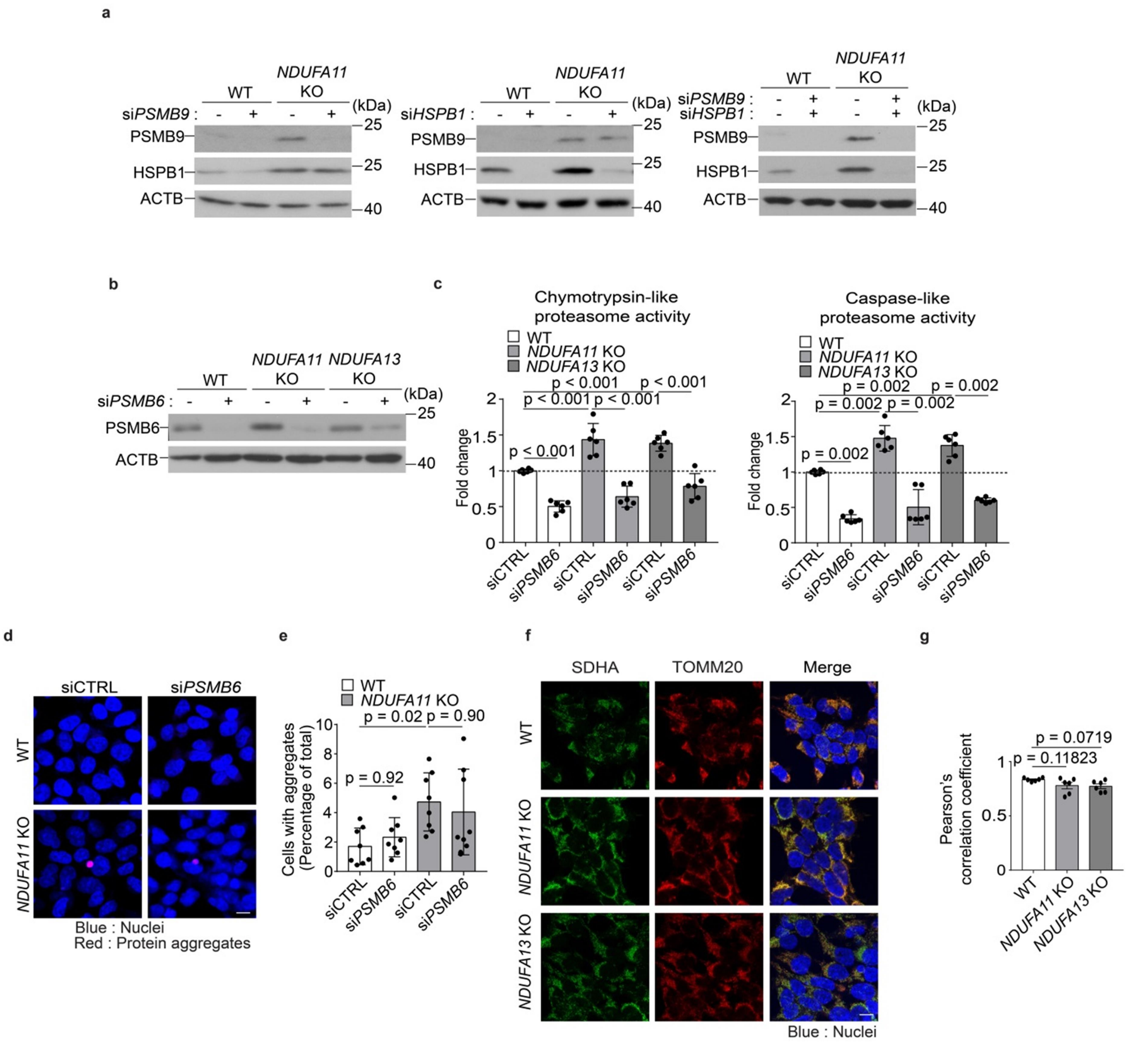

Supplementary Figure 9. **PSMB6 is not responsible to prevent protein aggregates in *NDUFA11* KO HEK293T cells.** (**a**) Western blotting validation of knockdown in *NDUFA11* KO and WT HEK293T cells transfected with *PSMB9* (si*PSMB9*), *HSPB1* (si*HSPB1*) or control (siCTRL) siRNA for 72 h. Data shown are representative of three independent experiments. (**b**-**e**) Mitochondrial complex I-deficient and WT HEK293T cells were transfected with *PSMB6* (siPSMB6) or control (siCTRL) siRNA for 72 h. (**b**) Western blotting validation of *PSMB6* knockdown. Data shown are representative of three independent experiments. (**c**) Chymotrypsin-like and caspase-like proteasome activities in cell lysates presented as fold changes relative to WT. Data shown are mean ± SD (n=3 biological replicates with two technical replicates). *p*-value from an ordinary one-way ANOVA with Tukey's multiple comparisons test or Kruskal-Wallis test with Dunn's multiple comparisons test using

GraphPad Prism. (**d**) Images of protein aggregates stained with PROTEOSTAT®. Data shown are representative of three independent experiments. The scale bar represents 10 μm. (**e**) Quantification of the percentage of cells containing aggregates in (**d**). Counted cell numbers of WT are 1594, 2154 from; *NDUFA11 KO* are 2263, 2730 from left to right. The analysis was based on eight sight fields from three independent experiments. Data shown are mean ± SD. *p*-value from an ordinary one-way ANOVA with Tukey's multiple comparisons test using GraphPad Prism. (**f**) Images of SDHA and TOMM20 co-staining in WT and mitochondrial complex I-deficient cells. Data shown are representative of three independent experiments. The scale bar represents 10 μm. (**g**) Pearson's correlation coefficient in (**f**) of WT (n=88), *NDUFA11 KO* (n=94) and *NDUFA13 KO* (n=116) HEK293T cells. The analysis was based on six sight fields from three independent experiments. Data shown are mean ± SD. *p*-value from an ordinary one-way ANOVA with Dunnett's multiple comparisons test using GraphPad Prism. Source data are provided as a Source Data file.

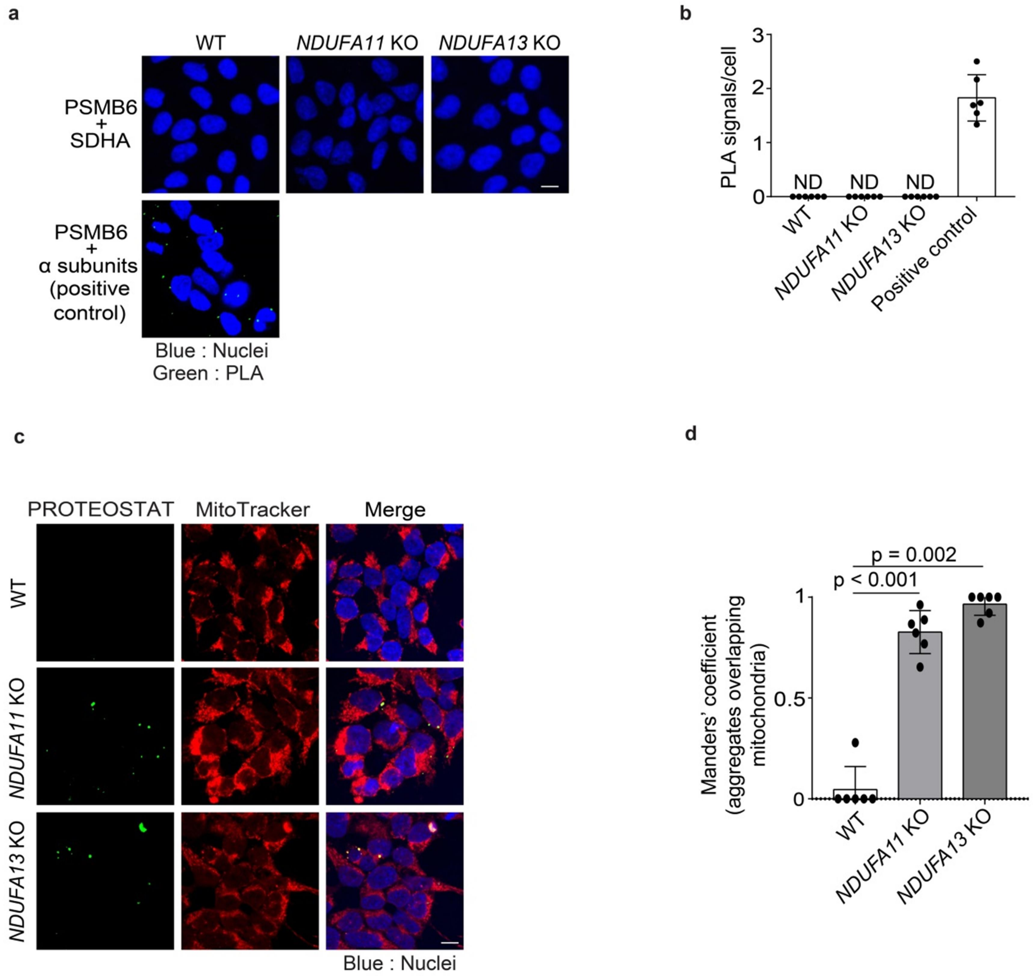

Supplementary Figure 10. Mitochondrial association with protein aggregation is PSMB6-independent. (**a**) Images of PLA performed between SDHA and PSMB6 in WT and mitochondrial complex I-deficient HEK293T cells and PLA performed between α-subunits and PSMB6 as a positive control in WT HEK293T cells. Data shown are representative of three independent experiments. The scale bar represents 10 μm. (**b**) Quantification of PLA signals per cell in (**a**) of WT (n=146), *NDUFA11 KO* (n=144), *NDUFA13 KO* (n=131) and a positive control (n=80) HEK293T cells. The analysis was based on nine sight fields in upper panel, six sight fields in lower panel from three independent experiments. Data shown are mean ± SD. ND, not detected. (**c**) Images of protein aggregates by PROTEOSTAT® co-stained with mitochondria by MitoTracker Deep Red in WT and mitochondrial complex I-deficient HEK293T cells. Data shown are representative of three independent experiments. The scale bar represents 10 μm. (**d**) Quantification of the percentage of cells containing

aggregates in (**c**) of WT (n=85), *NDUFA11 KO* (n=106) and *NDUFA13 KO* (n=104) HEK293T cells. The analysis was based on six sight fields from three independent experiments. Data shown are mean ± SD. *p*-value from two-sided, unpaired *t*-test or Mann Whitney test using GraphPad Prism. Source data are provided as a Source Data file.

Supplementary Table 1. The primers used in this study

Gene name	Forward primer (5’3’)	Reverse primer (3’5’)
HSPB1	AAGCTAGCCACGCAGTCCAA	CGACTCGAAGGTGACTGGGA
HSPA1A	ACCATTGAGGAGGTAGATTAGG	GCAAACACAGGAAATTGAGAAC
HSPA1B	ACTGTTGGGACTCAAGGAC	ATGAAGCCAGCTAATTACCATC
HSP90AA1	CTTGGGTCTGGGTTTCCTC	GGGCAACACCTCTACAAGGA
HSP90AB1	TGGCAGTCAAGCACTTTTCTGT	GCCCGACGAGGAATAAATAGC
HSPH1	ACCATGCTGCTCCTTTCTCC	CTGGGTTTTCTGGTGGTCTC
PSMB5	GGCAATGTCGAATCTATGAGC	GTTCCCTTCACTGTCCACGTA
PSMB6	CAAGCTGACACCTATTCACGAC	CGGTATCGGTAACACATCTCCT
PSMB7	ATCGCTGGGGTGGTCTATAAG	AAGAAATGAGCTGGGTTGTCAT
PSMB8	CACGGGTAGTGGGAACACTTA	ACTTTCACCCAACCATCTTCC
PSMB9	CAACGTGAAGGAGGTCAGGTA	AGAGCAATAGCGTCTGTGGTG
PSMB10	AATGTGGACGCATGTGTGAT	CATAGCCTGCACAGTTTCCTC
HSF1	AAGTGGTCCACATCGAGCAG	TCCTGGCGGATCTTTATGTCT
EEF1A1	TGTCGTCATTGGACACGTAGA	ACGCTCAGCTTTCAGTTTATCC
EEF1A2	TGCACCACGAGGCTCTGA	TGCTGTCCCCACACACGTT
ACTB	GCCGGGACCTGACTGACTAC	TTCTCCTTAATGTCACGCACGAT

Uncropped western blot images

Supplemental Figure 3c

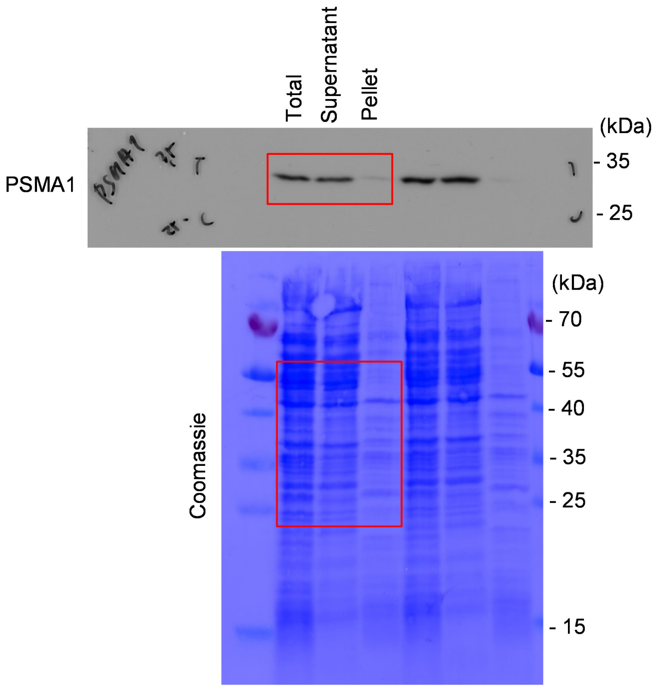

Supplementary Figure 4a

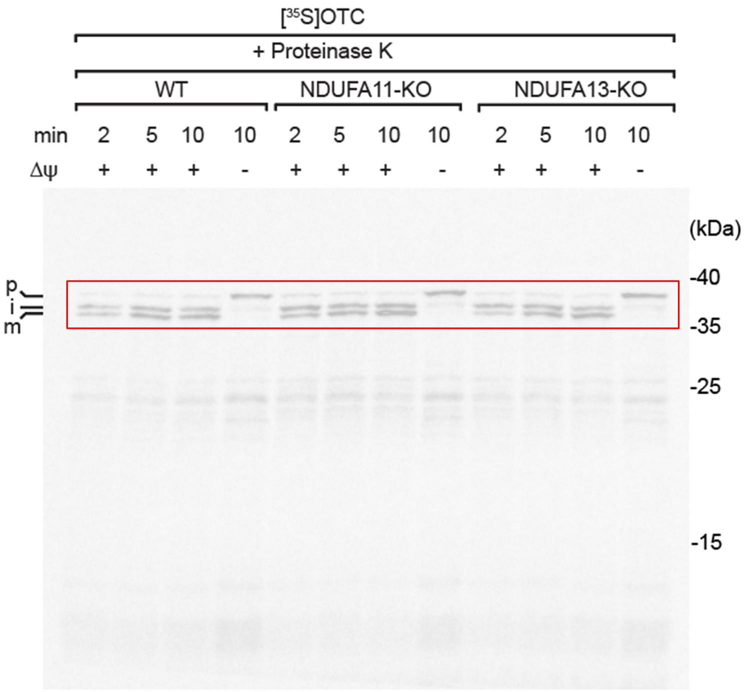

Supplementary Figure 5a

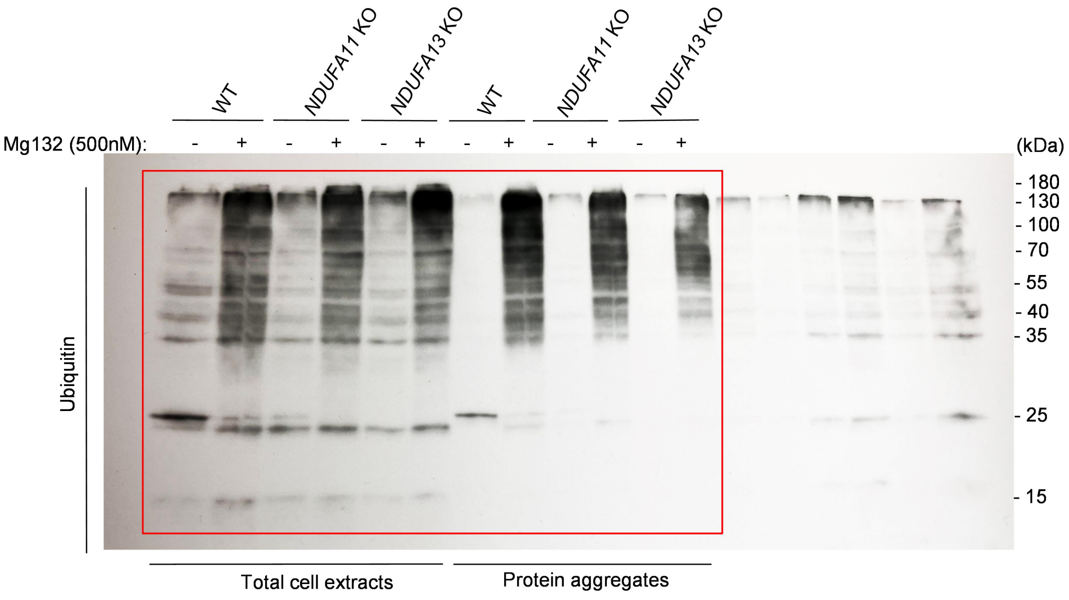

Supplementary Figure 6c

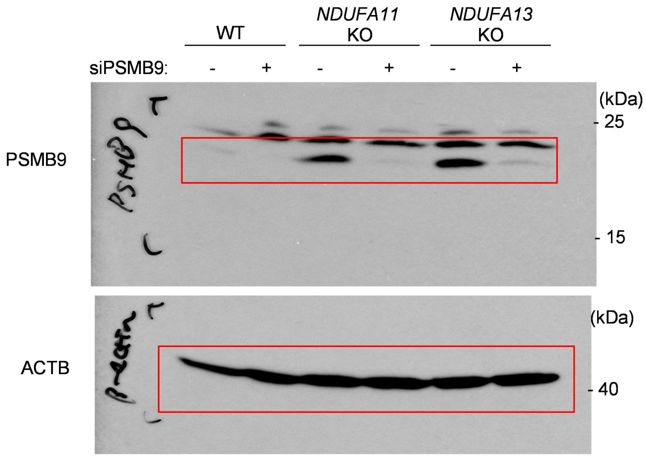

Supplementary Figure 6e

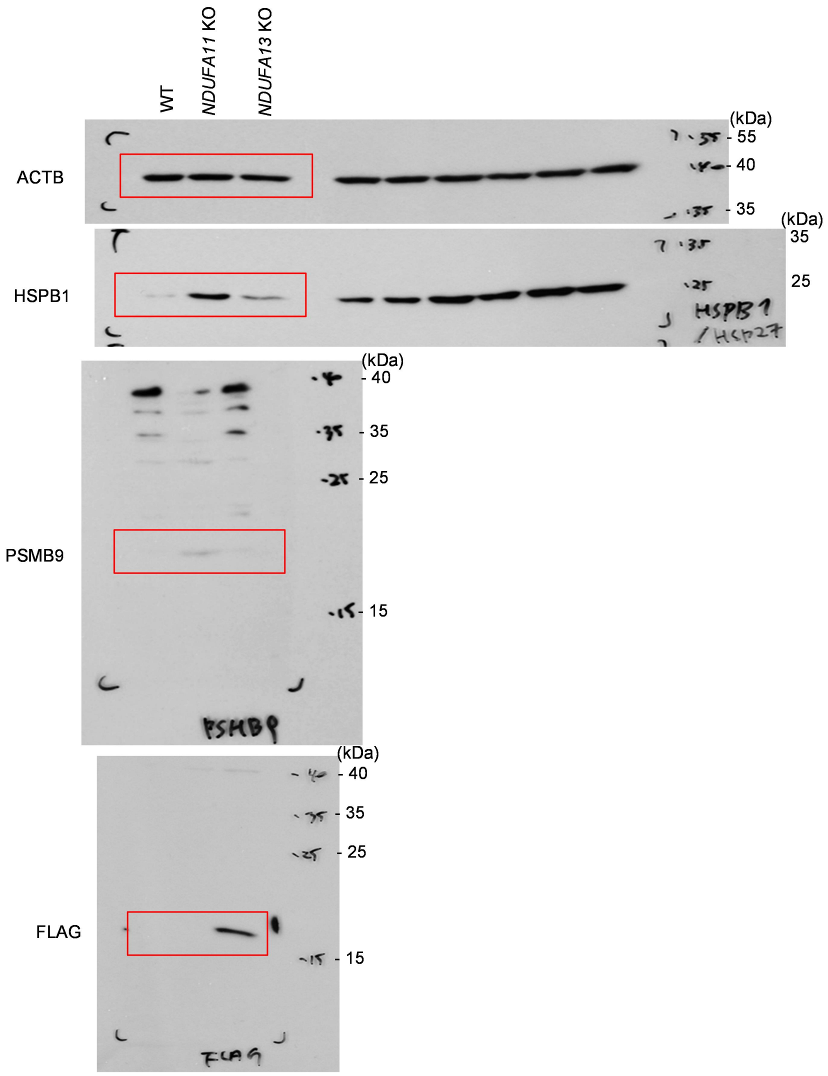

Supplementary Figure 6g

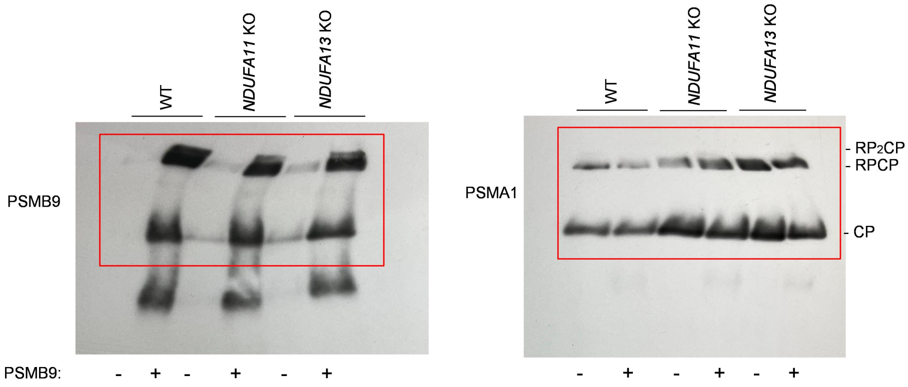

Supplementary Figure 6h

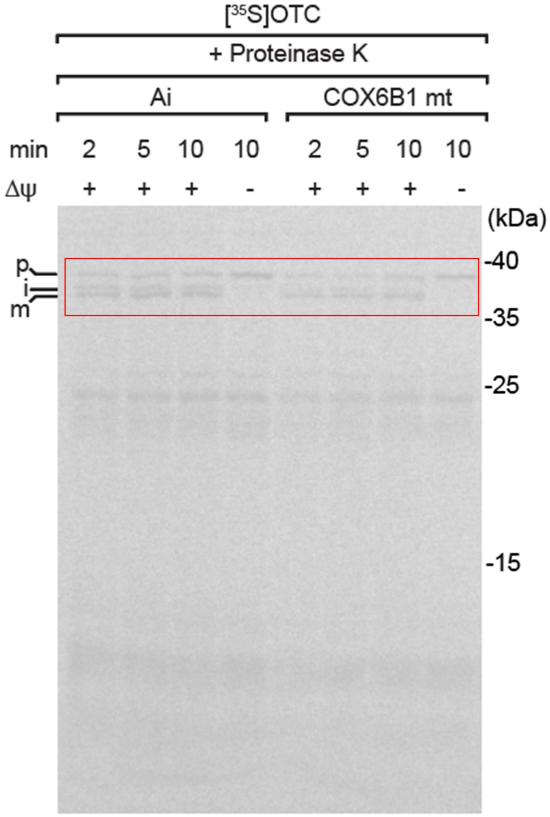

Supplementary Figure 7c

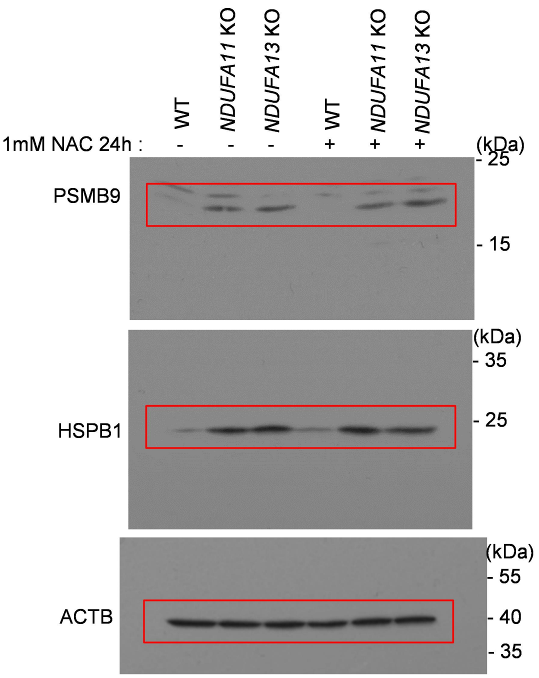

Supplementary Figure 7e

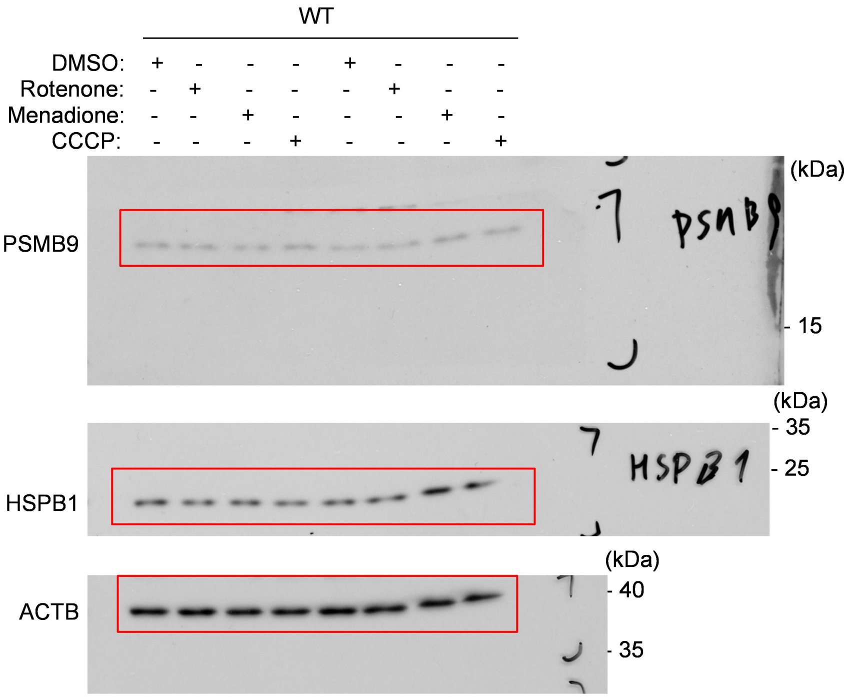

Supplementary Figure 8a

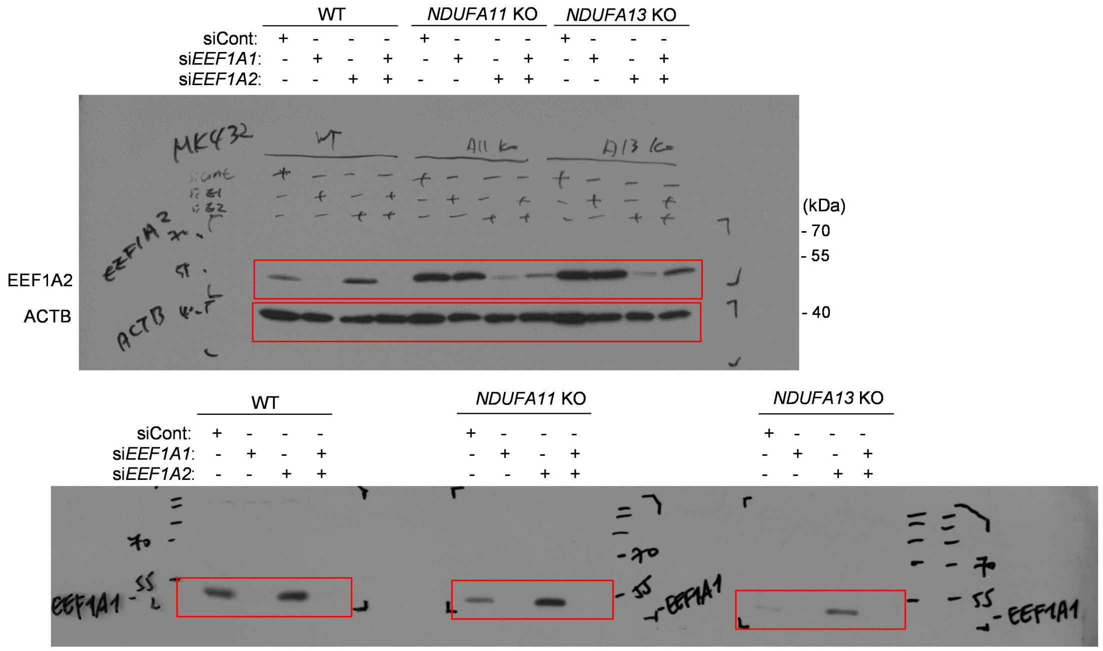

Supplementary Figure 8b

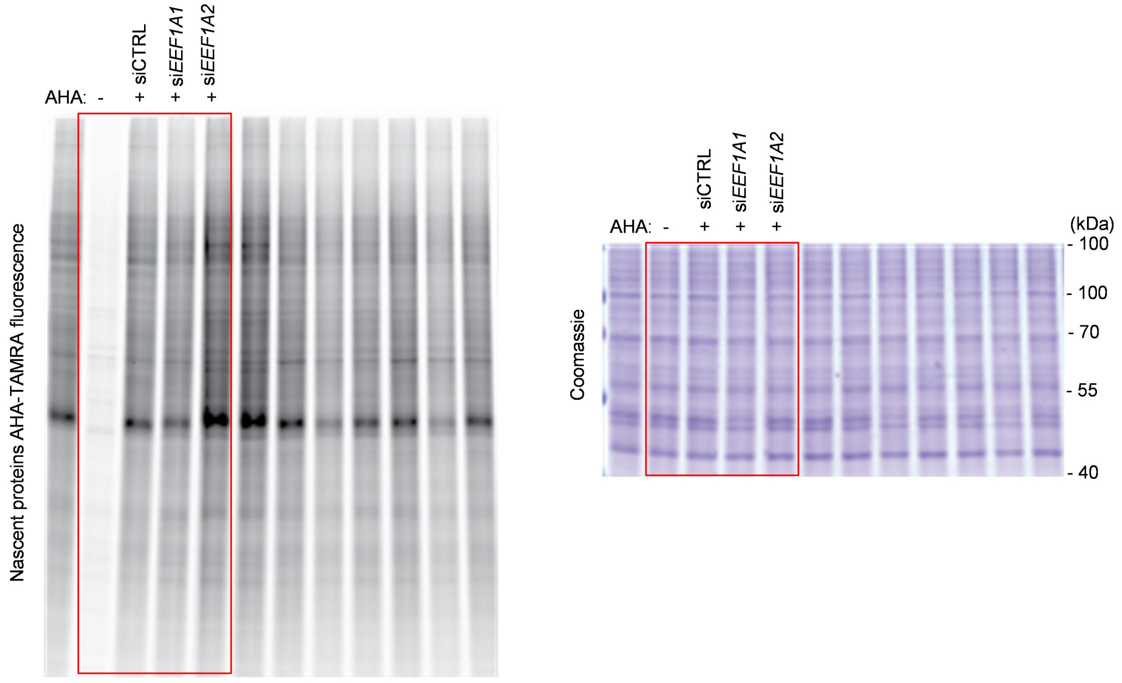

Supplementary Figure 8e

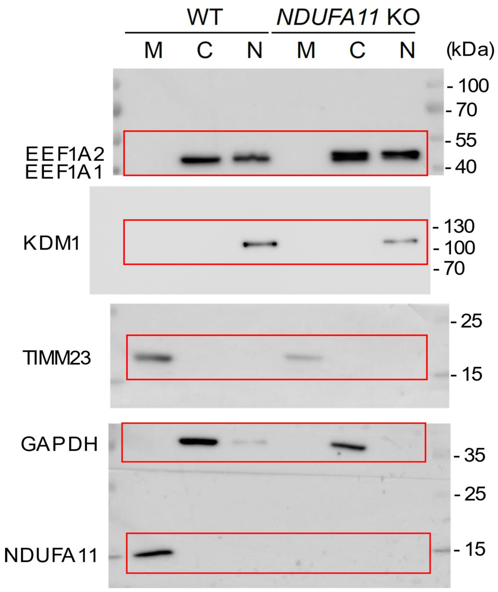

Supplementary Figure 9a

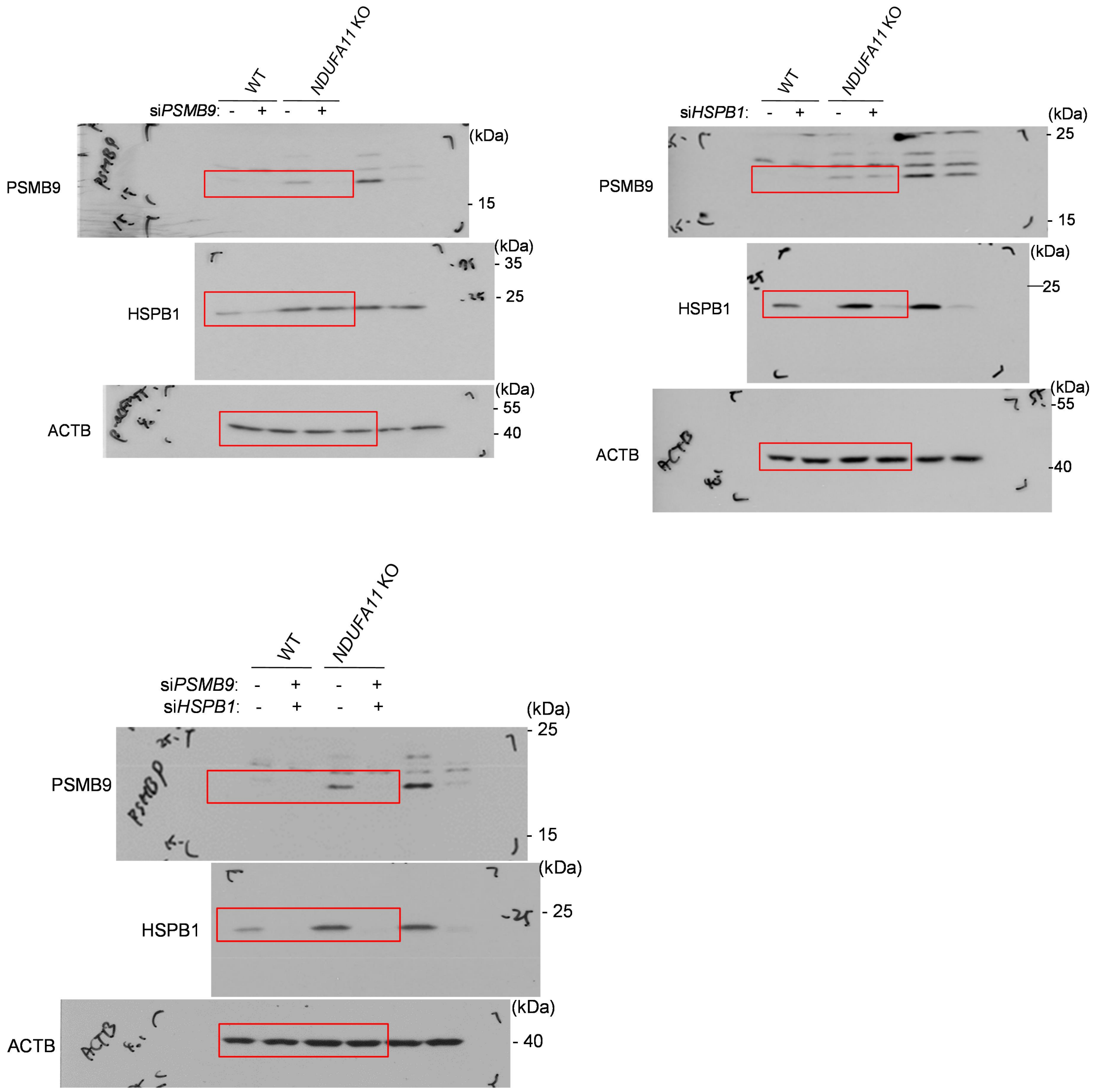

Supplementary Figure 9b

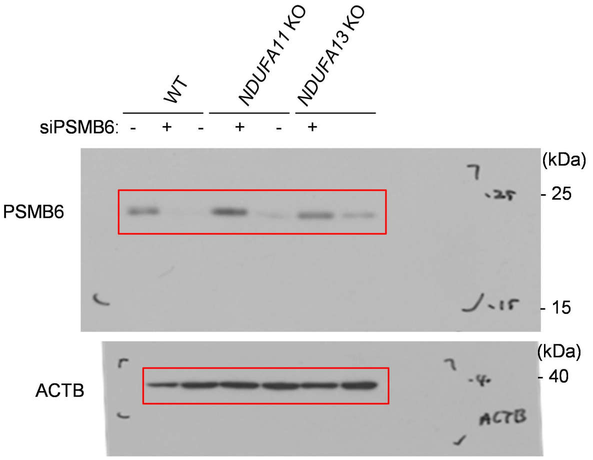

Supplement: Supplementary file 1 — Supplementary Information [file 41467_2023_39642_MOESM1_ESM.pdf]
