# Supplementary material for: Immunoproteasome-specific subunit PSMB9 induction is required to regulate cellular proteostasis upon mitochondrial dysfunction
Source: Nat Commun. 2023 Jul 11;14:4092. doi: 10.1038/s41467-023-39642-8 (PMC10336106; doi:10.1038/s41467-023-39642-8)
Supplement: Supplementary file 2 — Description of Additional Supplementary Files [file 41467_2023_39642_MOESM2_ESM.pdf]

Description of Additional Supplementary Files

File name: Supplementary Data 1.
Lists of genes identified and quantified in *NDUFA11* KO versus WT and *NDUFA13* KO versus WT experiments by RNA sequencing analysis.

File name: Supplementary Data 2.
Lists of proteins identified and quantified in total cell extracts and aggregates isolated from *NDUFA11* KO versus WT HEK 293T cells by LC-MS/MS analysis.

File name: Supplementary Data 3.
Lists of proteins identified and quantified in soluble, aggregates fractions isolated from *NDUFA11* KO versus WT HEK 293T cells, and mitochondrial proteins (High confidence mitochiondrial by MitoCoP) identified and quantified in mitochondrial fractions isolated from *NDUFA11 KO* versus WT HEK 293T cells by LC-MS/MS analysis.

File name: Supplementary Data 4.
Lists of proteins identified and quantified in isolated proteasomes from *NDUFA11* KO versus WT and NDUFA13 KO versus WT HEK 293T cells by LC-MS/MS analysis.
