# Supplementary material for: Immunoproteasome-specific subunit PSMB9 induction is required to regulate cellular proteostasis upon mitochondrial dysfunction
Source: Nat Commun. 2023 Jul 11;14:4092. doi: 10.1038/s41467-023-39642-8 (PMC10336106; doi:10.1038/s41467-023-39642-8)
Supplement: Supplementary file 7 — Reporting Summary [file 41467_2023_39642_MOESM7_ESM.pdf]

nature portfolio

Corresponding author(s): Agnieszka Chacinska

Last updated by author(s): Jun 5, 2023

Reporting Summary

Nature Portfolio wishes to improve the reproducibility of the work that we publish. This form provides structure for consistency and transparency in reporting. For further information on Nature Portfolio policies, see our Editorial Policies and the Editorial Policy Checklist.

Statistics

For all statistical analyses, confirm that the following items are present in the figure legend, table legend, main text, or Methods section.

n/a	Confirmed	
☐	☒	The exact sample size (*n*) for each experimental group/condition, given as a discrete number and unit of measurement
☐	☒	A statement on whether measurements were taken from distinct samples or whether the same sample was measured repeatedly
☐	☒	The statistical test(s) used AND whether they are one- or two-sided<br>*Only common tests should be described solely by name; describe more complex techniques in the Methods section.*
☒	☐	A description of all covariates tested
☐	☒	A description of any assumptions or corrections, such as tests of normality and adjustment for multiple comparisons
☐	☒	A full description of the statistical parameters including central tendency (e.g. means) or other basic estimates (e.g. regression coefficient) AND variation (e.g. standard deviation) or associated estimates of uncertainty (e.g. confidence intervals)
☐	☒	For null hypothesis testing, the test statistic (e.g. *F*, *t*, *r*) with confidence intervals, effect sizes, degrees of freedom and *P* value noted<br>*Give P values as exact values whenever suitable.*
☒	☐	For Bayesian analysis, information on the choice of priors and Markov chain Monte Carlo settings
☒	☐	For hierarchical and complex designs, identification of the appropriate level for tests and full reporting of outcomes
☐	☒	Estimates of effect sizes (e.g. Cohen's *d*, Pearson's *r*), indicating how they were calculated

Our web collection on statistics for biologists contains articles on many of the points above.

Software and code

Policy information about availability of computer code

Data collection	RNA sequencing data was collected by NovaSeq 6000 (Illumina), qRT-PCR data was collected by LightCycler480 (Roche), proteasome activity and ROS data were collected by Synergy H1 Hybrid Multi-Mode Microplate Reader (BioTek), mass spectrometry data was collected by Q Exactive HF-X mass spectrometer, Q Exective Plus mass spectrometer or Orbitrap Elite (Thermo Fisher), autoradiography images and fluorescent SDS-PAGE signals were collected by Typhoon FLA9500 (GE), western blotting data was collected by OPTIMAX2010 (Protec) or Amersham Imager 600 RGB (GE), and microscopy data was collected by LSM700 (Zeiss).
Data analysis	Microsoft Excel 2010, GraphPad Prism 7.0, Trimmomatic (version 0.39), Hisat2, GATK (version 4.1.2.0), HTSeq-count (version 0.9.1) , DESeq2 package (version 1.16.1), R Biocunductor package ReactomePA (version 1.36), RankProd, Perseus (version 1.6.6.0, 1.6.10.0), MaxQuant (version 1.6.0.1, 1.6.7.0, 1.6.17.0), ImageQuant TL and Image) 1.52a software, ggplot2, ggrepel, pheatmap, R studio. Detailed information of data analysis is provided in the Methods section.

For manuscripts utilizing custom algorithms or software that are central to the research but not yet described in published literature, software must be made available to editors and reviewers. We strongly encourage code deposition in a community repository (e.g. GitHub). See the Nature Portfolio guidelines for submitting code & software for further information.

Data

Policy information about availability of data

All manuscripts must include a data availability statement. This statement should provide the following information, where applicable:

- Accession codes, unique identifiers, or web links for publicly available datasets
- A description of any restrictions on data availability
- For clinical datasets or third party data, please ensure that the statement adheres to our policy

RNA sequencing data have been deposited in GEO under accession codes GSE196068. Mapped reads were associated with transcripts from GRCh38 database (Ensembl, version 77). The mass spectrometry data for protein aggregates have been deposited to the ProteomeXchange Consortium via the PRIDE partner repository with the dataset identifier PXD031374. The mass spectrometry data for total cell extracts have been deposited to the ProteomeXchange Consortium via the PRIDE partner repository with the dataset identifier PXD038397. The mass spectrometry data for mitochondria, cytoplasmic soluble and aggregate fractions have been deposited to the ProteomeXchange Consortium via the PRIDE partner repository with the dataset identifier PXD038004. The mass spectrometry data for proteasome complexes have been deposited to the ProteomeXchange Consortium via the PRIDE partner repository with the dataset identifier PXD031282. All other data are available in the article, supplementary information and supplementary data files.

Research involving human participants, their data, or biological material

Policy information about studies with human participants or human data. See also policy information about sex, gender (identity/presentation), and sexual orientation and race, ethnicity and racism.

Reporting on sex and gender	Not applicable
Reporting on race, ethnicity, or other socially relevant groupings	Not applicable
Population characteristics	Not applicable
Recruitment	Not applicable
Ethics oversight	Not applicable

Note that full information on the approval of the study protocol must also be provided in the manuscript.

Field-specific reporting

Please select the one below that is the best fit for your research. If you are not sure, read the appropriate sections before making your selection.

☒ Life sciences ☐ Behavioural & social sciences ☐ Ecological, evolutionary & environmental sciences

For a reference copy of the document with all sections, see nature.com/documents/nr-reporting-summary-flat.pdf

Life sciences study design

All studies must disclose on these points even when the disclosure is negative.

Sample size	No sample size calculation was performed as this study does not involve animal or human subjects. Sample sizes were chosen based on previous experience and common practice in the field. Thousands to million cells were used per one sample. Cells Data displayed is three independent biological experiments unless stated otherwise in the corresponding figure legends.
Data exclusions	No data related to experiments in the manuscript were excluded.
Replication	All experiments performed in this study were reliably reproducible. All experiments performed three times independently unless stated otherwise in the corresponding figure legends.
Randomization	Randomization was not performed for our experiments. All tests were in-vitro, and cells were identified by their genotype and/or treatment. Cells were cultured and harvested under identical conditions.
Blinding	Blinding was not possible as knockout cells have an obvious phenotype (slow growing).

Reporting for specific materials, systems and methods

We require information from authors about some types of materials, experimental systems and methods used in many studies. Here, indicate whether each material, system or method listed is relevant to your study. If you are not sure if a list item applies to your research, read the appropriate section before selecting a response.

Materials & experimental systems

n/a	Involved in the study
☐	☒ Antibodies
☐	☒ Eukaryotic cell lines
☒	☐ Palaeontology and archaeology
☒	☐ Animals and other organisms
☒	☐ Clinical data
☒	☐ Dual use research of concern
☒	☐ Plants

Methods

n/a	Involved in the study
☒	☐ ChIP-seq
☒	☐ Flow cytometry
☒	☐ MRI-based neuroimaging

Antibodies

Antibodies used

The primary antibodies used in western blot analysis were as follows: GFP (Roche, cat. no. 11814460001, 1:500 or 1:1000), HSPB1 (Abcam, cat. no. ab2790, 1:500), HSPA1A/HSP1AB (Enzo Life Sciences, cat. no. ADI-SPA-812-F, 1:2,000), HSP90 (Abcam, cat. no. ab13495, 1:1,000), HSPH1 (Abcam, cat. no. ab109624, 1:500), ACTB (Sigma, cat. no. A1978, 1:2,000), NDUFA11 (Abcam, cat. no. ab183707, 1:500), NDUFA13 (Abcam, cat. no. ab110240, 1:500), PSMB5 (Enzo Life Sciences, cat. no. BML-PW8895-0100, 1:500), PSMB6 (Abcam, cat. no. ab150392, 1:500), PSMB8 (Abcam, cat. no. ab3329, 1:500), PSMB9 (Abcam, cat. no. ab3328, 1:500), PSMA1 (Abcam, cat. no. ab3325, 1:500), PSMD1 (Abcam, cat. no. ab2941, 1:4,000), Proteasome 20S alpha 1+2+3+5+6+7 antibody (Abcam, cat. no. ab22674, 1:1,000), Ubiquitin (Santa cruz, cat. no. sc-8017, 1:500), EEF1A1 (Proteintech, cat. no. 11402-1-AP, 1:1,000), EEF1A2 (Thermo Fisher Scientific, cat. no. PAS-27677, 1:500), FLAG (Sigma, cat. no. F1804, 1:500), UQCRC1 (Sigma, cat. no. HPA002815, 1:500), COX5B (Santa scuz, cat. no. sc-374417, 1:500), GAPDH (Santa Cruz, cat. no. sc-47724, 1:500), NDUFS1 (Santa cruz, cat. no. sc-50132, 1:1,000), TUBA1C (Santa cruz, cat. no. sc-134239, 1:1,000). The secondary horseradish peroxidase-conjugated anti-mouse (Sigma, cat. no. A4416, 1:5,000 or 1:10,000), and anti-rabbit (Sigma, cat. no. A6154, 1:5,000 or 1:10,000), and anti-goat (Sigma, cat. no. A5420, 1:10,000) were used. The secondary horseradish peroxidase-conjugated anti-mouse (Sigma, cat. no. A4416, 1:5,000 or 1:10,000), anti-rabbit (Sigma, cat. no. A6154, 1:5,000 or 1:10,000)), and anti-goat (Sigma, cat. no. A5420, 1:10,000) were used.
The primary antibodies used in immunofluorescence were TOMM20 (Santa Cruz, cat. no. sc-11415, 1:100) and SDHA (Santa Cruz, cat. no. sc-166947, 1:100), and the second antibodies were Alexa Fluor" 647 (Invitrogen, cat. no. A-21245, 1:200) and Alexa Fluor" 488 (Invitrogen, cat. no. A-11029, 1:200).

Validation

All antibodies used in this study are commercially available and react with human samples and applicable to western blotting according to manufacturer's website. HSPB1 (Abcam, cat. no. ab2790, 1:500), NDUFA11 (Abcam, cat. no. ab183707, 1:500), NDUFA13 (Abcam, cat. no. ab110240, 1:500), PSMB6 (Abcam, cat. no. ab150392, 1:500), PSMB9 (Abcam, cat. no. ab3328, 1:500), PSMD1 (Abcam, cat. no. ab2941, 1:4,000), NDUFS1 (Santa cruz, cat. no. sc-50132, 1:1,000), EEF1A1 (Proteintech, cat. no. 11402-1-AP, 1:1,000) and EEF1A2 (Thermo Fisher Scientific, cat. no. PAS-27677, 1:500) were validated in our laboratory (knockout or knockdown validation).

Eukaryotic cell lines

Policy information about cell lines and Sex and Gender in Research

Cell line source(s)

HEK293T cells (wild-type, NDUFA11 KO, NDUFA13 KO) and human fibroblasts (control, COX6B1 mutant) were kind gifts from the laboratory of Prof. Michael T. Ryan (Monash University, Australia) and the laboratory of Prof. Massimo Zeviani (University of Cambridge, UK), respectively.

Authentication

Cell lines were not authenticated by ourselves.

Mycoplasma contamination

All cell lines were tested to be Mycoplasma negative.

Commonly misidentified lines
(See ICLAC register)

No commonly misidentified cell lines were used in this study.
